# Identification and Validation of Two Heterogeneous Molecular Subtypes and a Prognosis Predictive Model for Hepatocellular Carcinoma Based on Pyroptosis

**DOI:** 10.1155/2022/8346816

**Published:** 2022-08-28

**Authors:** Minshan Lai, Qiang Liu, Wenbo Chen, Xuxin Qi, Jianfeng Yang, Li Jiang, Meng Yuan, Zhichun Liu, Qiaojun He, Ji Cao, Bo Yang

**Affiliations:** ^1^Institute of Pharmacology & Toxicology, Zhejiang Province Key Laboratory of Anti-Cancer Drug Research, College of Pharmaceutical Sciences, Zhejiang University, Hangzhou 310058, China; ^2^Polytechnic Institute, Zhejiang University, Hangzhou 310058, China; ^3^Department of Gastroenterology, Affiliated Hangzhou First People's Hospital, Zhejiang University School of Medicine, Hangzhou 310006, China; ^4^Innovation Institute for Artificial Intelligence in Medicine, Zhejiang University, Hangzhou 310058, China; ^5^Cancer Center of Zhejiang University, Hangzhou 310058, China

## Abstract

Hepatocellular carcinoma (HCC) is a worldwide malignant cancer with high incidence and mortality. Considering the high heterogeneity of HCC, clarifying molecular characteristics associated with HCC development could help improve patients' outcomes. Pyroptosis is a novel form of cell death and is noted to be implicated in HCC pathogenesis whereas its molecular feature in HCC is unclear. Thus, we intended to clarify the molecular characteristic as well as the clinical significance of pyroptosis for HCC. A systematic bioinformatics analysis was conducted among 40 pyroptosis-related genes based on The Cancer Genome Atlas, the International Cancer Genome Consortium, and the Gene Expression Omnibus databases. A total of 12 HCC-associated pyroptosis-related genes (HPRGs) were identified to be overexpressed in HCC tissues and significantly connected to patients' poor survival. Through consensus clustering based on the HPRGs' expression, we found patients could be stratified into two distinctive pyroptosis subtypes, PyLow and PyHigh. The PyHigh group owned a notable lower survival rate and a higher high-grade proportion compared with the PyLow subtype. Besides, patients' sensitivities to chemotherapeutic drugs also presented distinctive differences between the two subtypes. Indicated by pathway enrichment analysis and immune characteristic difference analysis, the distinctions between the pyroptosis subtypes may be related to tumor immunity. Further, a five-gene risk model composed of *BAK1*, *CHMP4A*, *CHMP4B*, *DHX9*, and *GSDME* was established. Subsequent analyses demonstrated that the model could credibly classify patients as low or high risk and was an independent prognostic indicator for HCC. Abnormal expressions of the five genes were validated by biological experiments and new bioinformatics analysis. In conclusion, this study recognized and verified two heterogeneous pyroptosis subtypes and a predictable prognosis model for HCC. Our work may help facilitate the clinical management and treatment of HCC and understand the functions of pyroptosis in oncology.

## 1. Introduction

Liver cancer is a worldwide malignant cancer with high incidence and mortality, representing the fourth contributing cause of cancer deaths [1]. Hepatocellular carcinoma (HCC), accounting for 75%-85% of liver cancer cases, is the dominant type of liver cancer [2]. Although the progress of early diagnosis and comprehensive treatment has been achieved in recent years, HCC patients' prognosis remains poor [3]. One of the major reasons accounting for challenges in clinical management and treatment is the high molecular heterogeneity of HCC [4–6]. Therefore, clarifying molecular characteristics associated with HCC development may help improve patients' clinical outcomes.

Pyroptosis is a new type of proinflammatory programmed cell death, featured by cell swelling, lysis, and release of cellular proinflammatory contents [7]. Pyroptosis is mainly induced by two inflammasome pathways, caspase-1 dependent or caspase-1 independent (depending on caspase-4/5 in human or caspase-11 in mouse) [8]. Recently, pyroptosis was noted to play an essential role in tumor pathogenesis and exhibit potential clinical significance for many cancers [9]. For instance, pyroptosis-based signature models were established to possibly serve as prognostic indicators for gastric cancer [10], colorectal cancer [11], and ovarian cancer [12]. In colorectal cancer [13], gastric cancer [14], and bladder cancer [15], patients could be stratified into two or three heterogeneous pyroptosis molecular subtypes. In HCC, pyroptosis was reportedly engaged in tumor pathogenesis [16], but its molecular characterization remains largely explored.

Herein, we aimed to systematically investigate the molecular classification potential as well as prognostic and therapeutic values of pyroptosis for HCC patients. A comprehensive bioinformatics analysis was conducted based on The Cancer Genome Atlas (TCGA) and the International Cancer Genome Consortium (ICGC) databases utilized as training and validation cohorts, respectively. A sum of 40 pyroptosis-related genes (PRGs) gathered from the Molecular Signatures Database (MSigDB) were analyzed [17]. Based upon the expression data of PRGs and the survival profiles of HCC patients, we identified two distinctive pyroptosis subtypes and constructed a prognosis predictive model.

## 2. Materials and Methods

### 2.1. Data Downloading

mRNA expression and clinical profiles of HCC cases were extracted from TCGA database through the UCSC Xena (including 50 adjacent and 374 HCC specimens) (http://xena.ucsc.edu/) and the ICGC database (including 260 HCC samples) (https://dcc.icgc.org/). After removing samples replicated, expression data lost, overall survival (OS) data lost, or survival time less than 30 days, only 343 HCC samples in TCGA and 228 HCC samples in ICGC were employed. Before analyzing, gene expression was converted to transcripts per million (TPM) [18]. Genes related to pyroptosis were extracted from MSigDB (https://www.gsea-msigdb.org/gsea/msigdb/) [17]. There were 18 and 27 genes in GOBP_PYROPTOSIS and REACTOME_PYROPTOSIS, respectively. After removing duplicated genes from the two gene sets, a sum of 40 genes were left and defined as PRGs.

### 2.2. Identifying HCC-Associated PRGs

The Wilcoxon test was adopted to measure the expression difference of PRGs between normal and HCC samples. The log-rank test was conducted to estimate the prognostic effects of PRGs employing the “survival” package [19]. Kaplan-Meier (KM) curves were plotted by the “survminer” package [20]. Only PRGs with *P* values in the above analyses less than 0.05 were defined as HCC-associated PRGs (HRPGs).

### 2.3. Consensus Clustering

On the basis of the HPRGs' expression, HCC patients were classified into *k* (2 to 9) clusters utilizing the “ConsensusClusterPlus” package [21]. The optimal clustering number of samples was determined according to the slow growth rate of cumulative distribution function (CDF) value. Using the HPRGs as a gene list, the integrated pyroptosis score for each patient was computed by the single sample gene set enrichment analysis (ssGSEA) algorithm in the “GSVA” package [22]. Differences of integrated pyroptosis score and HRPG expression between the subtypes were analyzed by the Wilcoxon test. To inspect pyroptosis pattern difference between the subtypes, t-distributed stochastic neighbor embedding (t-SNE) was performed using the “Rtsne” package [23]. t-SNE is a data dimensionality reduction algorithm that can separate or condense samples into various disparate groups based upon the provided signatures or hallmarks [24].

### 2.4. Clinical Values of the Molecular Classification

Survival rate differences between the pyroptosis subtypes were estimated by the log-rank test. The chi-square test was applied to determine the associations between the pyroptosis subtypes and clinical characteristics. In this part, patients who lost information of age, gender, grade, or stage were removed. Finally, 317 and 209 samples were, respectively, left in TCGA and ICGC cohort. Drug sensitivity of chemotherapeutic drugs for HCC patients was assessed based on the Genomics of Drug Sensitivity in Cancer (GDSC) database (https://www.cancerrxgene.org/) using the half-maximal inhibitory concentration (IC_50_) predicted by the “pRRophetic” algorithm as an indicator [25]. The Wilcoxon test was adopted to assess drug sensitivity differences between the risk groups. *P* < 0.05 was considered meaningful.

### 2.5. Pathway Enrichment Analysis

Expression differences between the subtypes were evaluated with log_2_ (meanPyHigh–meanPyLow) (logFC) and *P* values calculated by the Wilcoxon test. *P* values were adjusted by the false discovery rate (FDR) method [26]. Genes with |logFC| > 1.5 and FDR < 0.001 were considered as differently expressed genes (DEGs). Kyoto Encylopedia of Genes and Genomes (KEGG) was carried out to explore pathways enriched among DEGs employing the “clusterProfiler” package [27]. *P* < 0.05 was considered meaningful.

### 2.6. Immune Characteristic Analysis

Tumor microenvironment (TME) components including StromalScore, ImmuneScore, and ESTIMATEScore were obtained by the ESTIMATE algorithm [28]. A list of 28 tumor-infiltrating lymphocytes (TILs) was downloaded from the TISIDB database (http://cis.hku.hk/TISIDB/index.php) [29]. Cytokine-related genes' (CRGs) list was gathered from the ImmPort database (https://www.immport.org/home) [30]. Patients' TIL abundance was estimated by the ssGSEA algorithm. Differences of TME components, TIL abundance, and CRG expression level between the subtypes were evaluated by the Wilcoxon test. *P* < 0.05 was regarded meaningful.

### 2.7. Predictive Risk Model Construction

The predictive risk model based on the HPRG expression was constructed by the least absolute shrinkage and selection operator (LASSO) penalized regression analysis employing the “glmnet” package [31, 32]. The risk score of each sample was computed based upon the model genes' TPM expression value and coefficient obtained by LASSO regression analysis. KM curve tested by log-rank test and ROC curve were drawn to evaluate the model's prognostic value for patients. Other HCC datasets with survival information, GSE14520 (*n* = 221), GSE76427 (*n* = 95), and GSE10143 (*n* = 80), were also downloaded from the Gene Expression Omnibus (GEO) database (http://www.ncbi.nlm.nih.gov/geo/) to test the model genes' performance. Univariate and multivariate Cox proportional hazards regression analyses of the risk score and conventional clinical features were carried out to test whether the risk score was an independent prognostic factor for HCC patients. Only variables with *P* < 0.05 in the univariate Cox analysis in both the databases were included in the multivariate Cox analysis.

### 2.8. Expression Differences' Validation

Expression differences of the model genes were validated through expression difference analysis based on GEO database, quantitative real-time PCR (qRT-PCR) analysis, and immunohistochemistry analysis. In expression difference analysis, mRNA expression profiles were extracted from three GEO datasets (GSE25097, GSE36376, and GSE45436, http://www.ncbi.nlm.nih.gov/geo/) and then tested by the Wilcoxon test. The number of HCC samples and normal samples is 268 and 243 in GSE25097, 240 and 193 in GSE36376, and 95 and 39 in GSE45436. In qRT-PCR analysis, total RNA was extracted using RNAiso Plus (TaKaRa) and reverse transcribed into cDNA with TransScript One-Step gDNA Removal and cDNA Synthesis SuperMix (TransGen Biotech) followed by qPCR detection using the SYBR Green qPCR Master Mix (Bio-Rad). Primers are listed in Table S1. Genes' expression levels were calculated by the 2 − ΔΔCt method and then analyzed by the Welch's *t*-test. Protein expression differences were investigated by comparing their staining level differences based on the immunohistochemistry analysis results from the Human Protein Atlas database (HPA, https://www.proteinatlas.org/) [33, 34].

### 2.9. Statistical Analysis

All analyses were carried out in the R 4.0.4 software except qRT-PCR data which was analyzed in the GraphPad Prism 8.0.1 software. All packages and parameters applied in the ICGC cohort were the same as those in TCGA cohort.

## 3. Results

### 3.1. Identification of HCC-Associated PRGs

**Figure 1:**
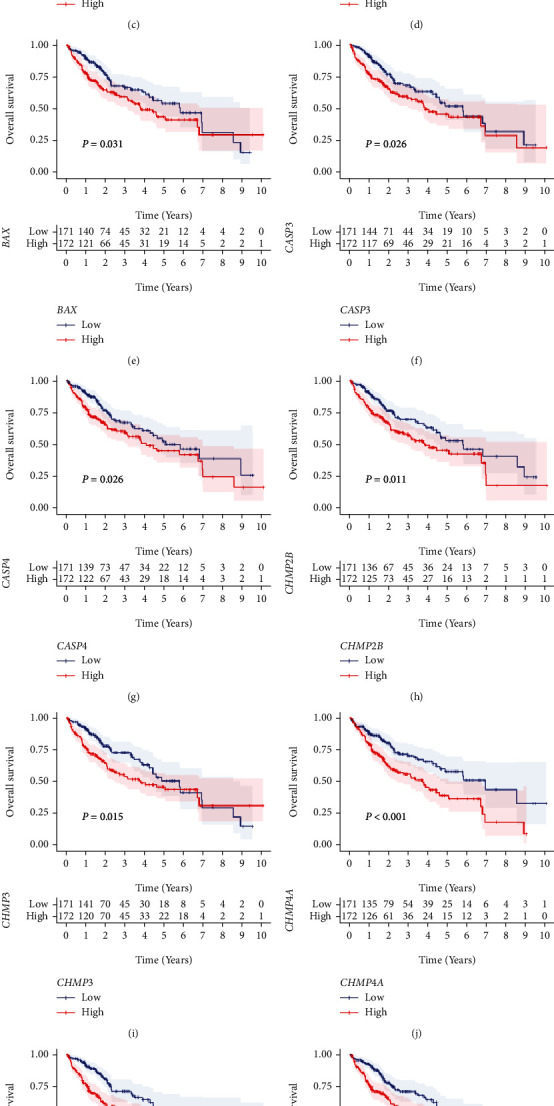
Identifying HPRGs. (a) The intention of this study. (b) Boxplot showing the expression alterations of the 40 PRGs between adjacent and HCC specimens in TCGA cohort. Wilcoxon test: ns: *P* > 0.05, ∗: *P* < 0.05, ∗∗: *P* < 0.01, ∗∗∗: *P* < 0.001, and ∗∗∗∗: *P* < 0.0001. (c–n) KM curves of the 12 HPRGs in TCGA cohort.

The intention of this study is shown in Figure 1(a). We aimed to explore the clinical significance including the molecular classification potential as well as the prognostic and therapeutic significances of pyroptosis for HCC patients. Firstly, differential expression analysis and log-rank test were conducted in TCGA cohort to identify PRGs involved in HCC pathogenesis. Results revealed that 31/40 PRGs expressed aberrantly in HCC samples (Wilcoxon test, *P* < 0.05, Figure 1(b) and Table S2), most of which were overexpressed in HCC tissues except AIM2, ELANE, and IL1B. According to KM curves, upregulation of 12/31 diagnostic PRGs, including APIP, BAK1, BAX, CASP3, CASP4, CHMP2B, CHMP3, CHMP4A, CHMP4B, GSDME, DHX9, and IL1A, exhibited a significant connection with a bad prognosis of patients (log-rank test, *P* < 0.05, Figures 1(c)–1(n) and Table S2). These 12 PRGs might be potential prognostic factors for HCC samples and were defined as HPRGs.

### 3.2. Identification of Pyroptosis Subtypes for HCC Patients

**Figure 2:**
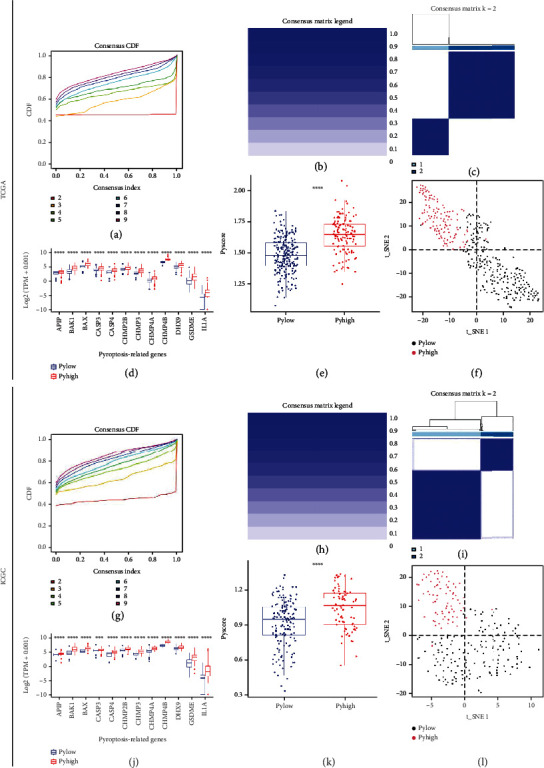
Consensus clustering based on the HPRGs. (a–c) Consensus clustering results in TCGA cohort. (a) CDF value of consensus index. (b) Consensus matrix legend. (c) Consensus matrix for *k* = 2. (d–f) Validation pyroptosis heterogeneity between the groups in TCGA cohort. (d) Expression variations of the 12 HPRGs between the two subtypes. Wilcoxon test, ^∗∗∗∗^*P* < 0.0001. (e) Pyroptosis score difference between the two subtypes. Wilcoxon test, ^∗∗∗∗^*P* < 0.0001. (f) Two-dimensional dot plot of t-SNE analysis based upon the 12 HPRGs' expression. (g–i) Consensus clustering results in the ICGC cohort. (g) CDF value of consensus index. (h) Consensus matrix legend. (i) Consensus matrix for *k* = 2. (j–l) Validation pyroptosis heterogeneity between the subtypes in the ICGC cohort. (j) Expression variations of the 12 HPRGs between the two subtypes. Wilcoxon test, ^∗∗∗∗^*P* < 0.0001. (k) Pyroptosis score difference between the two subtypes. Wilcoxon test, ^∗∗∗∗^*P* < 0.0001. (l) Two-dimensional dot plot of t-SNE analysis based upon the 12 HPRGs' expression.

Consensus clustering was performed in TCGA cohort based on the 12 HPRG expression level. According to the consensus CDF curve, HCC patients could be stratified into two pyroptosis subtypes for which *k* = 2 there was the flattest middle segment of the CDF curve (Figure 2(a)). The consensus matrix also confirmed that when *k* = 2 the consensus of intragroup was high and that of intergroup was low (Figures 2(b) and 2(c)). Since the expression level of each HRPG was notably higher in subtype 1 (Wilcoxon test, *P* < 0.0001, Figure 2(d)), we defined subtype 1 and subtype 2 as the PyHigh subtype and the PyLow subtype, respectively. Utilizing the 12 HPRGs as gene list, we enumerated an integrated pyroptosis score for each patient according to the ssGSEA algorithm. Differential analyses showed that the PyHigh group possessed remarkably a higher integrated pyroptosis score than the PyLow one (Wilcoxon test, *P* < 0.0001, Figure 2(e)), which is in line with the trend of HPRG expression discrepancies. Besides, the t-SNE analysis also demonstrated a distinctively different expression pattern of the 12 HPRGs between the two subtypes (Figure 2(f)).

To validate the classification effect in TCGA cohort, we also conducted consensus clustering in the ICGC cohort. Consequently, HCC patients were still clustered into two subtypes, subtype 1′ and subtype 2′ (Figures 2(g)–2(i)). According to the differentials of HPRG expression level as well as integrated pyroptosis score between the two subtypes, subtype 1′ was the PyLow group, and subtype2 ′ corresponded to the PyHigh subtype (Figures 2(j) and 2(k)). In t-SNE analysis, the two subtypes also presented a distinctive distribution (Figure 2(l)). Collectively, the above results demonstrated that HCC patients could be stratified into two heterogeneous pyroptosis status subtypes.

### 3.3. Prognostic and Therapeutic Values of the Pyroptosis Classification for HCC

**Figure 3:**
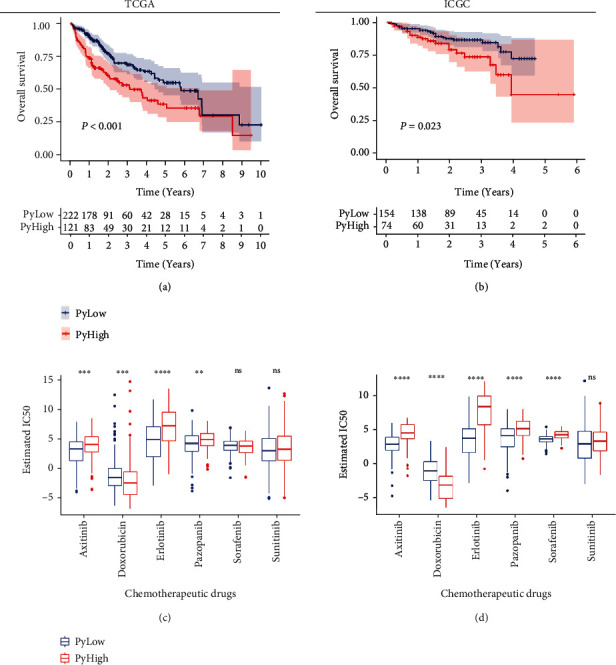
Clinical significances of the pyroptosis classification. (a, b) KM curves indicating the OS difference between the two subtypes in TCGA and ICGC cohorts. (c, d) Estimated IC_50_ value differences of chemotherapeutic drugs between the two subtypes in TCGA and ICGC databases. Wilcoxon test: ns: *P* > 0.05, ∗∗: *P* < 0.01, ∗∗∗: *P* < 0.001, and ∗∗∗∗: *P* < 0.0001.

The prognostic significance of the pyroptosis classification for HCC was further inspected. In TCGA cohort, the PyHigh subtype patients owned a significantly lower OS rate than the PyLow subtype ones (log-rank test, *P* < 0.001, Figure 3(a)). Similarly, the PyHigh subtype patients in the ICGC cohort were also in connection with a poorer prognosis (log-rank test, *P* = 0.023, Figure 3(b)). What is more, differences in disease-free survival (DFS), progression-free survival (PFI), and disease-specific survival (DSS) between the subtypes were examined in TCGA cohort (these survival data were not available in the ICGC cohort). Similar to the KM analysis result of OS, the DSS rate in the PyHigh subtype was significantly shorter than that in the PyLow subtype (log-rank test, *P* = 0.033, Figure S1(a)). However, there was no significant differences on PFS or DFS (log-rank test, *P* = 0.072 and *P* = 0.096, respectively, Figures S1(b) and S1(c)). Further, associations between the clinicopathological features and the subtypes were investigated. As shown in Table 1, the proportion of high grade (G3+G4) was notably higher in the PyHigh subtype than in the PyLow subtype in both datasets (chi-square test, *P* = 0.003 and *P* = 0.003, Table 1). However, we were unable to detect significant association with other clinicopathological variables (chi-square test, *P* > 0.05, Table 1).

Drug sensitivity differences of 6 common chemotherapeutic drugs (Axitinib, Doxorubicin, Erlotinib, Pazopanib, Sorafenib, and Sunitinib) for HCC patients were tested between the two subtypes [35]. Applying the pRRophetic algorithm [25], we estimated the IC_50_ of the drugs based upon the GDSC database. Results indicated that Doxorubicin was more sensitive to the PyHigh subtype patients while Axitinib, Erlotinib, and Pazopanib presented more sensitivity to the PyLow subtype patients (Wilcoxon test, *P* < 0.05, Figure 3(e)). Analysis results in the ICGC cohort exhibited similar results to TCGA cohort.

### 3.4. Functional Analysis of Differentially Expressed Genes between the Pyroptosis Subtypes

**Figure 4:**
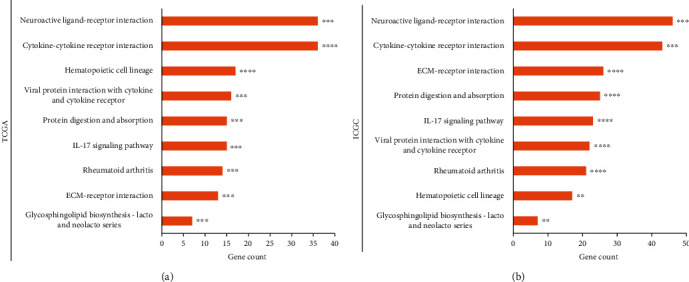
KEGG pathway enrichment. Bar plots of KEGG analysis results listing the enriched pathways among DEGs between the two subtypes in (a) TCGA database and (b) ICGC database. The *X*-axis indicates the count of DEGs included in the pathway. ∗∗: *P* < 0.01, ∗∗∗: *P* < 0.001, and ∗∗∗∗: *P* < 0.0001.

DEGs between the two subtypes were identified based on differential expression analysis. Then, enriched pathways among these DEGs were determined through the KEGG analysis. In differential expression analysis, 1119 genes were expressed aberrantly in TCGA cohort, among which 1063 genes were overexpressed in the PyHigh subtype (Table S3). In the ICGC cohort, 1659 and 72 genes were noted, respectively, up- and downregulated in the PyHigh subtype (Table S4). Pathway analysis in TCGA cohort indicated that several immune-relevant pathways, such as cytokine-cytokine receptor interaction, viral protein interaction with cytokine and cytokine receptor, and IL-17 signaling pathway, were significantly enriched among the DEGs (*P* < 0.05, Figure 4(a)). Interestingly, the pathways enriched in TCGA cohort were also remarkably enriched in the ICGC database (*P* < 0.05, Figure 4(b)). These results indicated that the potential mechanisms of the pyroptosis subtype affecting patients' survival and drug sensitivity may be related to immunity. Thus, we further explored the differences in immune characteristics between the pyroptosis subtypes.

### 3.5. Immune Characteristic Differences between the Pyroptosis Subtypes

**Figure 5:**
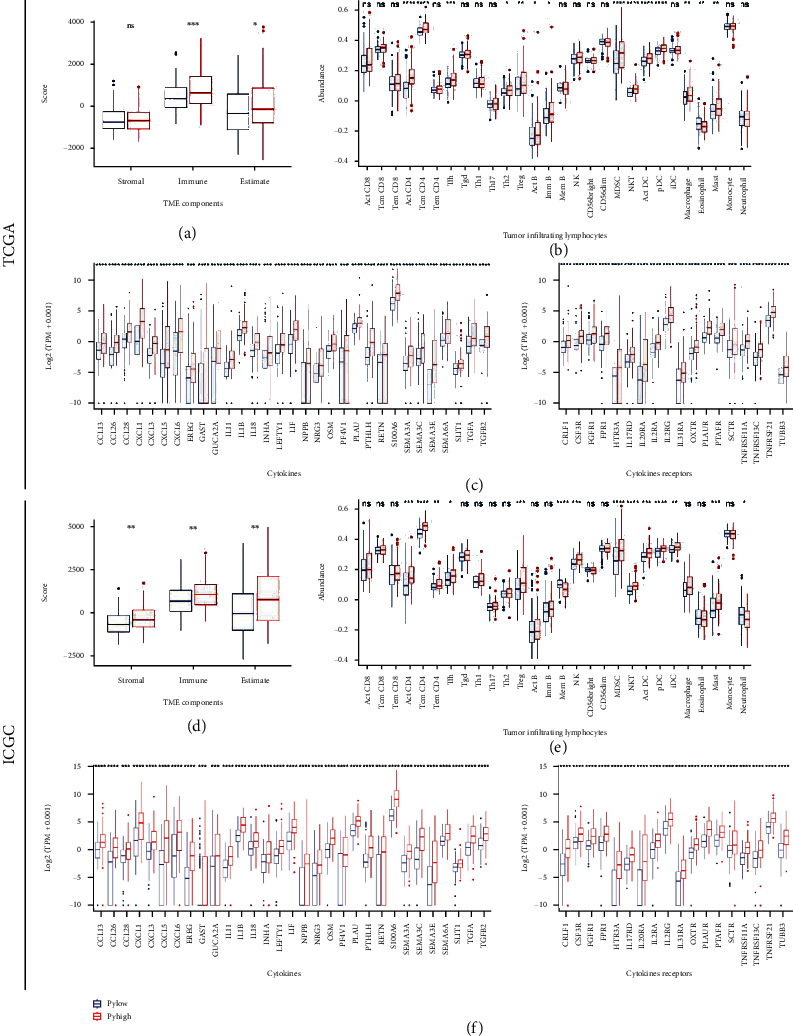
Immune characteristic difference between the subtypes. (a–c) Immune characteristic difference analyses in TCGA cohort. Wilcoxon test: ns: *P* > 0.05, ∗: *P* < 0.05, ∗∗: *P* < 0.01, ∗∗∗: *P* < 0.001, and ∗∗∗∗: *P* < 0.0001. (a) TME component differences. (b) 28 TIL abundance differences. (c) CRG expression differences. (d–f) Immune characteristic difference analyses in the ICGC cohort. Wilcoxon test: ns: *P* > 0.05, ∗: *P* < 0.05, ∗∗: *P* < 0.01, ∗∗∗: *P* < 0.001, and ∗∗∗∗: *P* < 0.0001. (d) TME component differences. (e) 28 TIL abundance differences. (f) CRG expression differences.

Immune characteristic differences between the pyroptosis subtypes were compared on three levels: TME components, TIL abundance, and CRG expression level. In TME component analyses, the PyHigh subtype harbored notably higher ImmuneScore and ESTIMATEScore than the PyLow subtype in both TCGA and ICGC databases (Wilcoxon test, *P* < 0.05, Figures 5(a) and 5(d)). The abundance of several TILs, including activated CD4 T cell, central memory CD4 T cell, natural killer T cell, regulatory T cell, myeloid derived suppressor cell, activated dendritic cell, plasmacytoid dendritic cell, and mast cell, was significantly richer in the PyHigh subtype in both the two cohorts (Wilcoxon test, *P* < 0.05, Figures 5(b) and 5(e)). Since cytokine-related pathways were notably enriched in KEGG analysis, we also investigated expression differences of CRGs between the subtypes. Results suggested that there were 31 cytokines and 18 cytokine receptors simultaneously expressed aberrantly in the two cohorts, and all of them were overexpressed in the PyHigh subtype (Wilcoxon test, |logFC| > 1.5, FDR < 0.001, Figures 5(c) and 5(f)). In summary, TME components, TIL abundance, and CRG expression level were markedly richer in the PyHigh group than in the PyLow group.

### 3.6. Construction of Pyroptosis Signature Predictive Model

**Figure 6:**
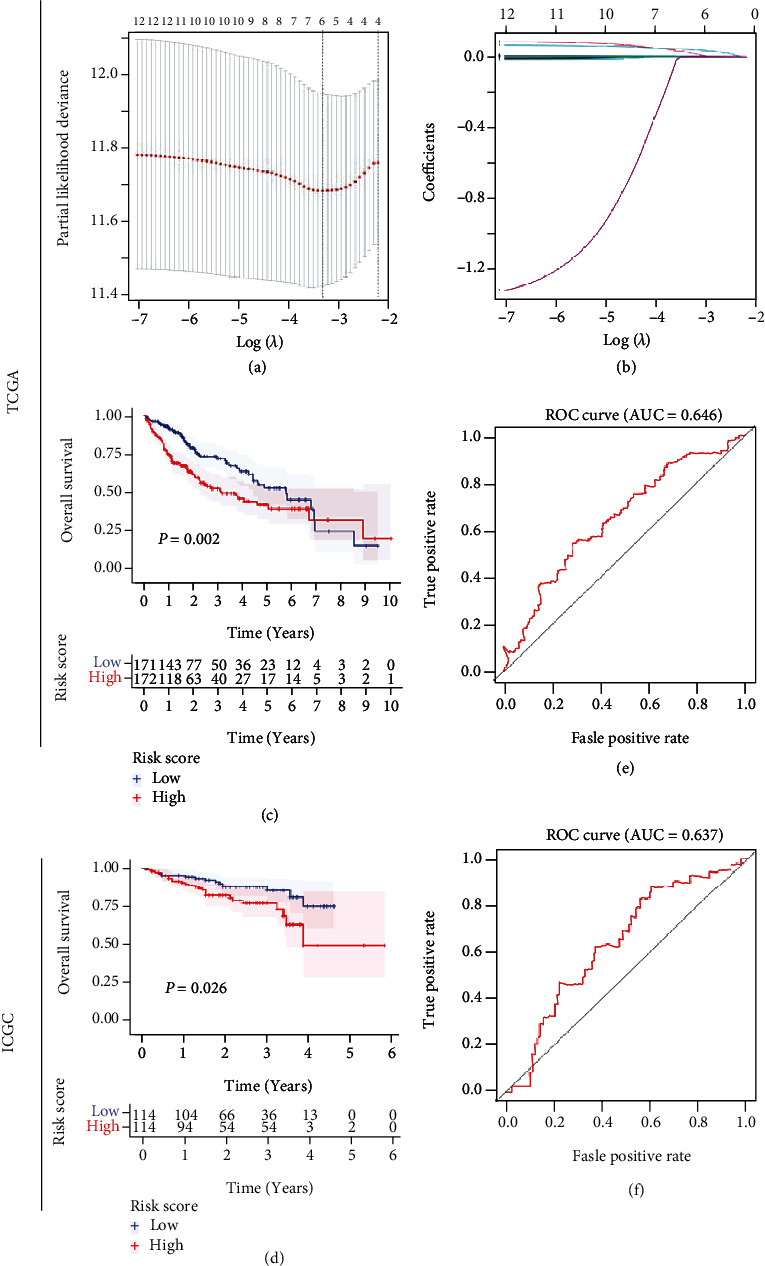
Constructing pyroptosis signature prognostic model. (a) Selection of *λ* based upon the partial likelihood deviance. (b) LASSO coefficient spectrum of the 12 HPRGs. (c, d) KM curves showing prognosis discrepancies between the low- and high-risk groups in TCGA and ICGC cohorts. (e, f) ROC curves of the forecast model in TCGA and ICGC cohorts.

To evaluate the joint effect of the 12 HRPGs on patients' survival, we established a multigene prognostic model by conducting the LASSO penalized regression analysis in TCGA cohort. Consequently, a 5-gene signature was identified according to the optimal value of *λ* (Figures 6(a) and 6(b)). Each patient's risk value was computed following the formula risk score = (0.0041 × BAK1_Exp_) + (0.0227 × CHMP4A_Exp_) + (0.0006 × CHMP4B_Exp_) + (0.0044 × DHX9_Exp_) + (0.0406 × GSDME_Exp_). Based upon the median risk value, samples were separated into low- or high-risk groups. Survival analysis indicated that the high-risk group was markedly correlated to the lower OS rate of patients (log-rank test, *P* = 0.002, Figure 6(c)). Further, we imputed patients' risk values in the ICGC database utilizing the same formula in TCGA cohort and divided them into two risk groups. Similarly, patients' prognosis in the high-risk group was noticeably poorer than in the low-risk group (log-rank test, *P* = 0.026, Figure 6(d)). Besides, area under the ROC curves (AUCs) in the two cohorts were both more than 0.6 (0.646 in TCGA, and 0.637 in ICGC, Figures 6(e) and 6(f)) [36]. The model's performances on predicting patients' DSS, PFS, and DFS in TCGA cohort were also estimated. KM curves demonstrated that the high-risk group was also significantly associated with the lower DSS and PFS rate of patients (log-rank test, *P* = 0.007 and *P* = 0.033, respectively, Figures S2(a) and S2(b)), but the DFS rate showed no significant difference (log-rank test, *P* = 0.238, Figure S2(c)). AUCs of DSS, PFS, and DFS were 0.665, 0.564, and 0.547, respectively, (Figures S2(d)–S2(f)). Based on a microarray dataset, GSE76427, patients' OS difference between the low-risk and high-risk groups was not significant (log-rank test, *P* > 0.05, Figure S3(a)) and the AUC value was less than 0.6 (Figure S3(b)). To test the model's generalization ability to other tumors, we analyzed another digestive tract cancer, pancreatic cancer (PAAD) from TCGA database, for the reason that the pancreas and liver have been reported to probably share differentiation patterns [37]. Results demonstrated that patients' OS rate in the high-risk group was significantly lower than that in the low-risk group (log-rank test, *P* = 0.024, Figure S3(c)) and the AUC reached 0.647 (Figure S3(d)). We also conducted survival analysis for the individual model genes (*GSDME*, *BAK1*, and *DHX9*) which cooccurred in the HCC datasets with survival information, GSE14520, GSE76427, and GSE10143. Results showed that high expression of *GSDME* and *DHX9* in GSE14520 was significantly associated with patients' poorer OS (log-rank test, *P* = 0.016 and *P* = 0.017, respectively, Figures S4(a) and S4(b)) consistent with the results in TCGA, while the others showed no significant difference (log-rank test, *P* > 0.05, Figures S4(c)–S4(i)).

To test the genetic selection consistency, we also performed elastic net regression analysis among the 12 HPRGs as suggested by a published article [38]. As a result, a total of six genes were selected out (Table S5). Interestingly, the five genes selected by the LASSO algorithm were all included and ranked in top five in terms of coefficients. What is more, to test whether the predictive ability of the model was independent of confounding factors, we carried out univariate and multivariate Cox regression analyses among the risk score and clinicopathological features. Results demonstrated that the risk score was significantly associated with patients' OS in both the cohorts (univariate analysis: *P* = 0.008 and *P* = 0.020 in TCGA and the ICGC cohort, respectively, Table 2; multivariate analysis: *P* = 0.007 and *P* = 0.033 in TCGA and the ICGC cohort, respectively, Table 3). Further, when dividing patients into two groups, early stage and advanced stage, the risk model still performed well (in TCGA, 0.619 and 0.682 for stage I+II and stage III+IV patients, respectively; in ICGC, 0.670 and 0.627 for stage I+II and stage III+IV patients, respectively, Figures S3(c) and S3(d)). These indicated that the risk score is an independent prognostic indicator for HCC patients.

### 3.7. Validating the Model Genes' Expression Differences

**Figure 7:**
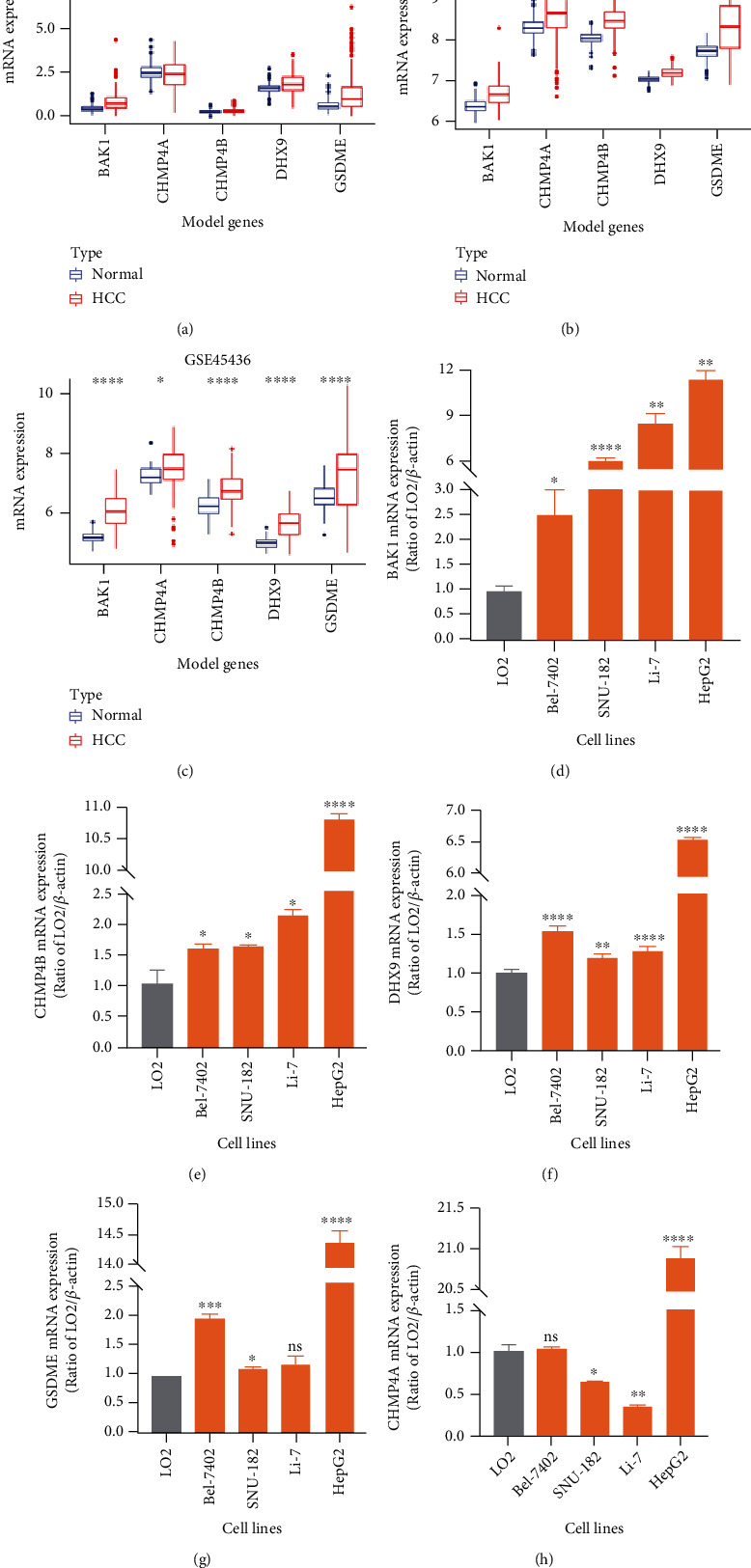
Validating the model genes' expression differences at mRNA level. Genes' expression alterations between adjacent and HCC tissues in the (a) GSE25097, (b) GSE36376, and (c) GSE45436 datasets. Wilcoxon test: ^∗^*P* < 0.05, ^∗∗∗^*P* < 0.001, and ^∗∗∗∗^*P* < 0.0001. (d–h) Genes' relative expression differences between normal liver cell line (LO2) and four HCC cell lines (Bel-7402, SNU-182, Li-7, and HepG2) based on the qRT-PCR experiment. Welch's *t*-test: ns: *P* > 0.05, ∗: *P* < 0.05, ∗∗: *P* < 0.01, ∗∗∗: *P* < 0.001, and ∗∗∗∗: *P* < 0.0001.

**Figure 8:**
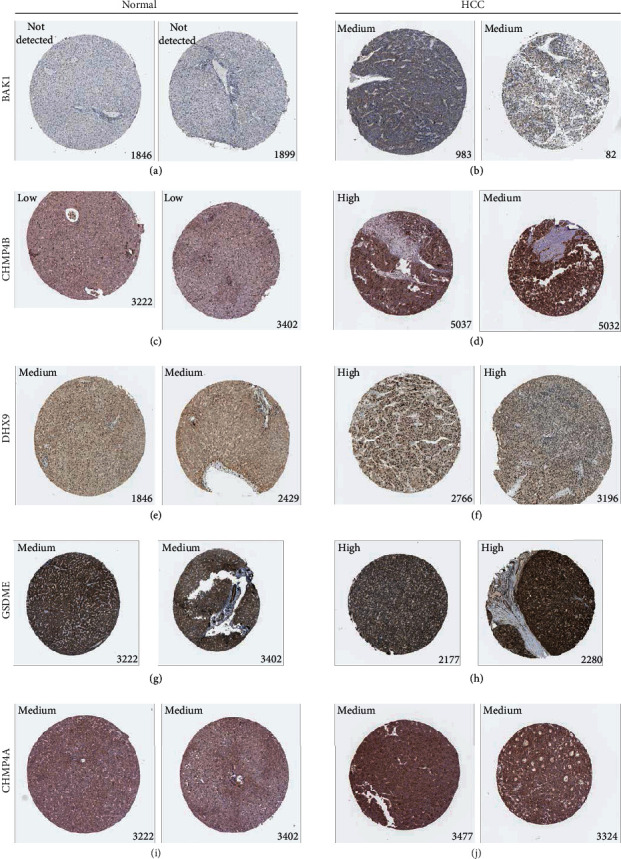
Validating the model genes' expression differences at protein level. (a–j) Immunohistochemistry results from the HPA database presenting protein expression level differences of the model genes between normal liver tissues and HCC ones. The text in the upper left corner represents the staining level, and the number in the lower right corner indicates the patient id.

To verify the five model genes' abnormal expressions, we preformed several different analyses. In mRNA expression difference analysis based on GEO databases, all the model genes were significantly overexpressed in HCC tissues in GSE25097, GSE36376, and GSE45436 datasets (Wilcoxon test, *P* < 0.05, Figures 7(a)–7(c)). In the qRT-PCR experiment, mRNA expression levels of BAK1, CHMP4B, and DHX9 were significantly higher in all experimental HCC cell lines than in normal one while GSDME and CHMP4A were only higher in three and one HCC cell lines, respectively, (Welch's *t*-test, *P* < 0.05, Figures 7(d)–7(h)). Besides, genes' expression differences at the protein level were also detected by immunohistochemistry results from the HPA database. Consistently, BAK1, CHMP4B, DHX9, and GSDME were upregulated in HCC tissues while CHMP4A showed no significant staining level difference (Figures 8(a) and 8(j)).

## 4. Discussion

Tumor-promoting inflammation is one of the ten well-known characteristics of cancer [39]. As a new type of proinflammatory cell death, pyroptosis has gained rising prominence in cancer research recently [9]. In HCC, pyroptosis was also noted to be engaged in tumor pathogenesis [16]. In this study, we systematically clarify the classification potential as well as prognostic and therapeutic values of pyroptosis for HCC patients via a comprehensive bioinformatics analysis based on TCGA and ICGC databases. We found that HCC patients could be stratified into two heterogeneous pyroptosis clusters, the PyHigh subtype and the PyLow subtype. Compared with the PyLow group, the PyHigh subtype presented significantly higher pyroptosis expression pattern, poorer prognosis, different drug sensitivities, and richer immune abundance. Besides, we constructed a novel multigene predictive model for HCC patients and validated the model genes' expression differences through several different analyses. Recently, some similar studies about different PRG research in HCC subtype identification [40–46] and predictive model construction [40–49] have been published. However, in all the previous subtype identification studies, the cluster analyses were conducted only in one cohort while we also tested in a validation cohort. Besides, further feature analyses between the subtypes in those studies were limited, leaving other features such as drug sensitivity unclear. Moreover, the association between the pyroptosis pattern and other features of the subtypes were not elucidated. In the predictive model construction studies, most of them did not perform expression validation for the model genes [41–48]. In a word, we have conducted in-depth analyses for the molecular subtypes and validated the mode genes' expression by various analyses which makes our results more reliable and practical in HCC clinical application as well as its future research.

Usually, pyroptosis is reckoned to be induced by caspase-1-dependent or caspase-1-independent ways with GSDMD as the effector. In recent years, a novel avenue, namely, the caspase-3-GSDME axis, has been identified. In the pathway, activation of caspase-3 could induce GSDME cleavage and thus result in pore formation and ultimately trigger pyroptosis [9]. According to our results, both CASP3 and GSDME exhibited as potential poor prognosis indicators for HCC patients, while GSDMD showed no significant effect on patients' OS. Moreover, GSDME contributed the most to predicting patients' risk with a coefficient of 0.0406 in the HPRG combined model. Interestingly, the association of caspase-3-GSDME axis pyroptosis with liver disease has also been noted in previous studies. GSDME, but not GSDMD, was found to be essential for miltirone-triggered pyroptosis in inhibiting HCC cell lines' viability [50]. The treatment effect of As_2_O_3_ on HCC cells was reported closely related to caspase-3-dependent GSDME pyroptosis [51]. In another study, GSDME-derived caspase-3 inhibitors were found to capably protect mice from acute hepatic failure [52]. Based on the above, we supposed that pyroptosis playing an effect on HCC might be tightly correlated with the caspase-3-GSDME pathway.

Pyroptosis is a double-edged sword for tumors [53]. It can on the one hand stimulate tumor proliferation through harboring a suitable microenvironment for tumor cell growth and on the other hand suppress tumor development via triggering pyroptotic cell death [54]. In our study, high pyroptosis expression pattern mainly acted as a bad indicator for HCC patients' prognosis. For example, high expression of HPRGs was all notably in connection with the poor OS of HCC patients. The coefficient of each HRPG in the risk model is positive, implying their harmful effect on patients' prognosis. Consensus clustering is a common unsupervised clustering method widely applied in molecular stratification for cancer based on gene expression data [55]. In the field of pyroptosis, it has also been utilized in molecular classification for colorectal cancer [13], gastric cancer [14], and bladder cancer [15]. Applying the method in HCC, we found that patients could be stratified into two distinctive pyroptosis subtypes based on HPRGs' expression. Moreover, the PyHigh subtype patients exhibited a lower OS rate and a higher high-grade proportion than the PyLow group ones.

In terms of mechanism, pyroptosis affecting HCC patients' prognosis and progress are inseparable from tumor immunity. In our study, several results from multiangle have implicated it. First, several immune-relevant biological functions, such as viral protein interaction with cytokine and cytokine receptor, were remarkably enriched. Second, TME components, TIL inflation level, and CRG expression conformably harbored more abundant in the PyHigh group in both the databases. It is well acknowledged that hepatitis B and C viruses are the two crucial risk causes for HCC [56]. They can affect HCC development in many ways, such as inflammation induction [57]. As an important component of the immune system and released in response to infection, inflammation, and carcinogen-induced injury, cytokine was found critically in promoting HCC carcinogenesis and progression [58]. Cytokine activity was a key signal of severity and development of hepatitis B or C virus infections [59]. Differential infiltration levels of TILs between the subtypes mainly exhibited in dendritic cell and T cell. Coincidentally, dendritic cell is one of the major documented places where pyroptosis occurs [60]. Besides, GSDME-mediated pyroptosis was reportedly essential in triggering cytokine storm during chimeric antigen receptor T cell therapy [61]. In addition, according to previous researches, the pyroptosis subtypes of other cancers also harbored distinguished TME landscapes [13–15].

In the drug sensitivity test, 4 common chemotherapy drugs, Axitinib, Doxorubicin, Erlotinib, and Pazopanib, presented noticeably different sensitivities to the two subtypes. A previous study found that Erlotinib could elicit GSDME-modulated pyroptotic tumor cell death in lung cancer [62]. Overexpression of GSDME in cervical cancer cell could result in Doxorubicin-induced apoptosis shifting into pyroptosis in a CASP3-dependent manner [63]. In lung cancer cells, Doxorubicin was also reported to capably induce robust pyroptosis and GSDME cleavage [63]. Moreover, the sensitivity of Doxorubicin to melanoma cells was increased after silencing eEF-2K resulting in Doxorubicin-induced autophagy switching to GSDME-dependent pyroptotic cell death [64].

Nevertheless, there are some limitations of our research. For example, only the most applied mRNA expression data for tumor classification was included in HCC subtyping. Other omics data, such as gene mutation, which is also important to tumor heterogeneity, should be considered in the future. Secondly, some model genes' expression showed no significant association with patients' survival in some GEO datasets. Actually, since gene's expression was affected by various confounding factors, usually several genes combined together rather than single gene alone were utilized to conduct predictive model [65]. The risk model performed poorly in the GSE76427 dataset but did well in the TCGA-PAAD cohort. We suspect the methodology difference that TCGA and the ICGC utilize the RNA-sequencing method while the GEO datasets apply the microarray approach may account for the inconsistent results.

## 5. Conclusions

In summary, we have recognized and verified two heterogeneous pyroptosis subtypes and a multigene prognostic model for HCC patients. Mechanically, pyroptosis playing an effect on HCC development is closely related to tumor immunity. These results may help guide HCC clinical management and deepen the understanding of pyroptosis.

## Figures and Tables

**Table 1:** Associations between the clinicopathological characteristics and the pyroptosis subtypes.

Characteristic	TCGA (*N* = 317)			ICGC (*N* = 209)		
	PyLow (*n* = 205)	PyHigh (*n* = 112)	*P*	PyLow (*n* = 150)	PyHigh (*n* = 59)	*P*
*Age*						
≤65	128 (62.4%)	79 (70.5%)	0.185	54 (36.0%)	23 (39.0%)	0.808
>65	77 (37.6%)	33 (29.5%)		96 (64.0%)	36 (61.0%)	
*Gender*						
Female	60 (29.3%)	41 (36.6%)	0.225	42 (28.0%)	11 (18.6%)	0.221
Male	145 (70.7%)	71 (63.4%)		108 (72.0%)	48 (81.4%)	
*Grade*						
G1+G2	139 (67.8%)	56 (50%)	**0.003**	117 (78.0%)	33 (55.9%)	**0.003**
G3+G4	66 (32.2%)	56 (50%)		33 (22.0%)	26 (44.1%)	
*Stage*						
I+II	154 (75.1%)	80 (71.4%)	0.561	100 (66.7%)	33 (55.9%)	0.196
III+IV	51 (24.9%)	32 (28.6%)		50 (33.3%)	26 (44.1%)	

**Table 2:** Univariate Cox regression analyses of the risk score and clinicopathological features.

Characteristic	TCGA		ICGC	
	HR (95% CI)	*P*	HR (95% CI)	*P*
*Age*				
≤65	1	0.584	1	0.228
>65	1.115 (0.755–1.648)		1.551 (0.760–3.167)	
*Gender*				
Female	1	0.199	1	0.049
Male	0.776 (0.526–1.143)		0.502 (0.253–0.998)	
*Grade*				
G1+G2	1	0.611	1	0.003
G3+G4	1.105 (0.751–1.627)		2.702 (1.402–5.208)	
*Stage*				
I+II	1	0.001	1	0.004
III+IV	1.944 (1.312–2.881)		2.623 (1.357–5.072)	
*Risk score*				
Low	1	0.008	1	0.020
High	1.684 (1.146–2.473)		2.259 (1.136–4.496)	

HR: hazard ratio.

**Table 3:** Multivariate Cox regression analyses of the risk score and clinicopathological features.

Characteristic	TCGA		ICGC	
	HR (95% CI)	*P*	HR (95% CI)	*P*
*Stage*				
I+II	1	0.001	1	0.007
III+IV	1.97 (1.328–2.922)		2.483 (1.282–4.809)	
*Risk score*				
Low	1	0.007	1	0.033
High	1.705 (1.16–2.507)		2.121 (1.061–4.242)	

HR: hazard ratio.

## Data Availability

The databases utilized in the current research can be downloaded at https://portal.gdc.cancer.gov/ and http://xena.ucsc.edu/ (the TCGA-LIHC and TCGA-PAAD cohorts), https://dcc.icgc.org/ (the ICGC LIRI-JP cohort), and http://www.ncbi.nlm.nih.gov/geo/ (the GSE14520, GSE76427, GSE10143, GSE25097, GSE36376, and GSE45436 datasets).

## Supplementary Materials

Supplementary Materials Figure S1: (a–c) KM curves indicating the differences of DSS, PFS, and DFS between the two pyroptosis subtypes in TCGA cohort. Figure S2: (a–c) KM curves showing the prognosis discrepancies of DSS, PFS, and DFS between the low- and high-risk groups in TCGA cohort. (d–f) ROC curves of the risk model in predicting patients' DSS, PFS, and DFS in TCGA cohort. Figure S3: (a–d) KM curves and ROC curves of the risk model in the GSE76427 dataset (a, b) and the TCGA-PAAD cohort (c, d). (e, f) ROC curves of the risk model for different stages of patients in TCGA cohort (e) and the ICGC cohort (f). Figure S4: KM curves of *GSDME*, *BAK1*, and *DHX9* in the GSE14520 (a–c), GSE76427 (d–f), and GSE10143 (g–i) datasets. Table S1: primer sequences applied in the qRT–PCR experiment. Table S2: differential expression analysis and log-rank test results of the 40 PRGs in TCGA cohort. Table S3: differentially expressed genes (DEGs) between the two subtypes in TCGA cohort. Table S4: Differentially expressed genes (DEGs) between the two subtypes in the ICGC cohort. Table S5: coefficients of the six genes selected by the elastic net algorithm in TCGA cohort.
