# Supplementary material for: Identification and Validation of Two Heterogeneous Molecular Subtypes and a Prognosis Predictive Model for Hepatocellular Carcinoma Based on Pyroptosis
Source: Oxid Med Cell Longev. 2022 Aug 28;2022:8346816. doi: 10.1155/2022/8346816 (PMC9441383; doi:10.1155/2022/8346816)
Supplement: Supplementary Materials — Figure S1: (a–c) KM curves indicating the differences of DSS, PFS, and DFS between the two pyroptosis subtypes in TCGA cohort. Figure S2: (a–c) KM curves showing the prognosis discrepancies of DSS, PFS, and DFS between the low- and high-risk groups in TCGA cohort. (d–f) ROC curves of the risk model in predicting patients' DSS, PFS, and DFS in TCGA cohort. Figure S3: (a–d) KM curves and ROC curves of the risk model in the GSE76427 dataset (a, b) and the TCGA-PAAD cohort (c, d). (e, f) ROC curves of the risk model for different stages of patients in TCGA cohort (e) and the ICGC cohort (f). Figure S4: KM curves of GSDME, BAK1, and DHX9 in the GSE14520 (a–c), GSE76427 (d–f), and GSE10143 (g–i) datasets. Table S1: primer sequences applied in the qRT–PCR experiment. Table S2: differential expression analysis and log-rank test results of the 40 PRGs in TCGA cohort. Table S3: differentially expressed genes (DEGs) between the two subtypes in TCGA cohort. Table S4: Differentially expressed genes (DEGs) between the two subtypes in the ICGC cohort. Table S5: coefficients of the six genes selected by the elastic net algorithm in TCGA cohort. [file 8346816.f1.zip › Figure S1-S4.docx]

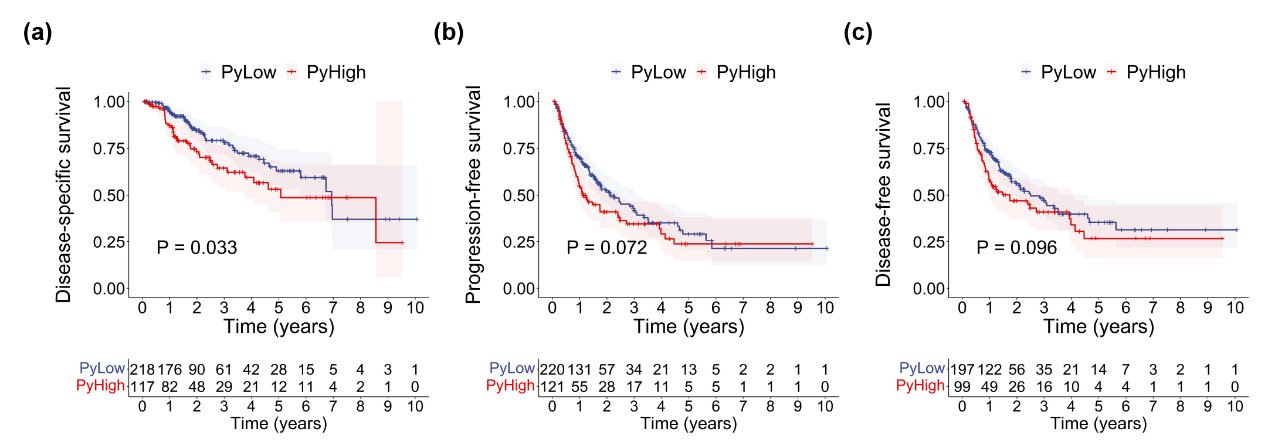
 Figure S1: (a-c) KM curves indicating the differences of DSS, PFS, and DFS between the two pyroptosis subtypes in the TCGA cohort.


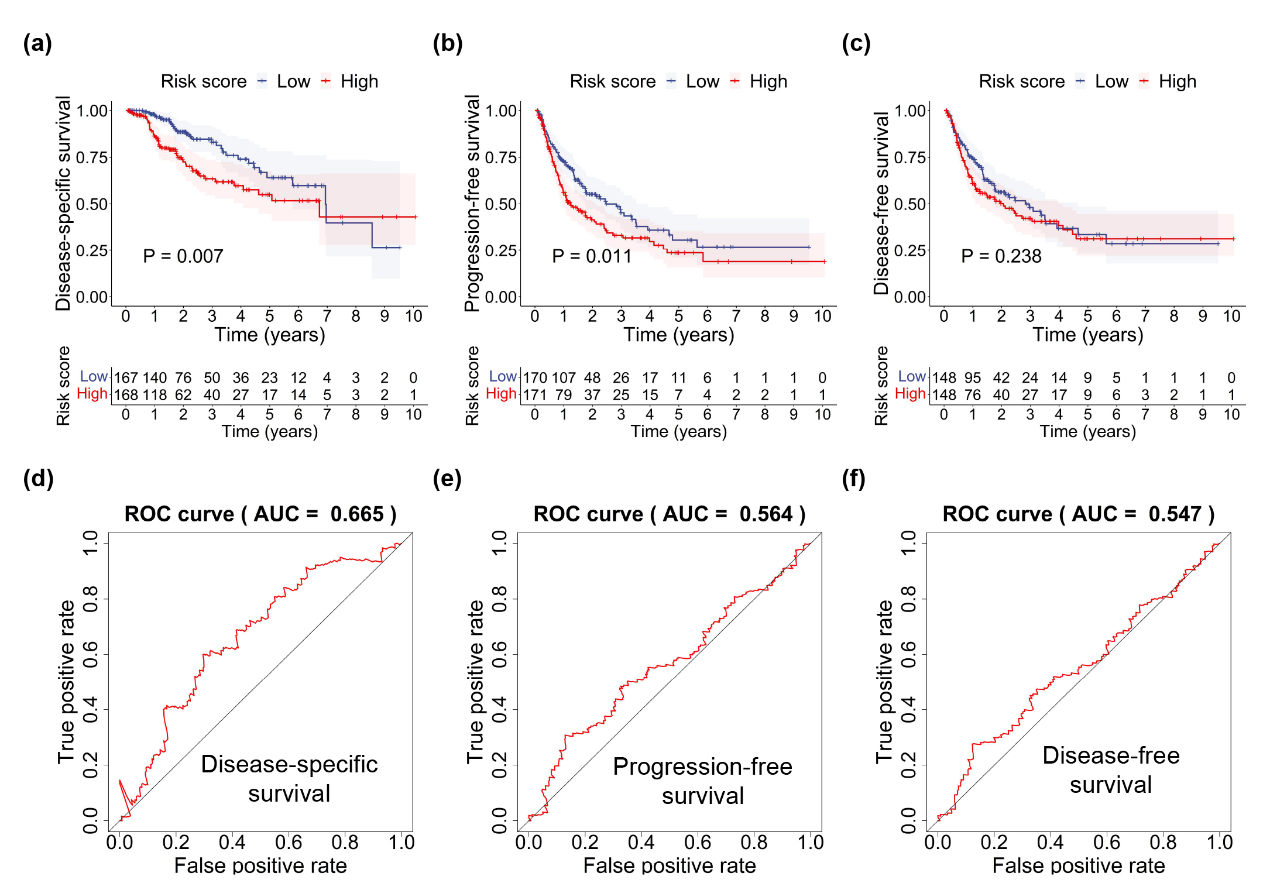
 Figure S2: (a-c) KM curves showing the prognosis discrepancies of DSS, PFS, and DFS between the low- and high-risk groups in the TCGA cohort. (d-f) ROC curves of the risk model in predicting patients’ DSS, PFS, and DFS in the TCGA cohort.


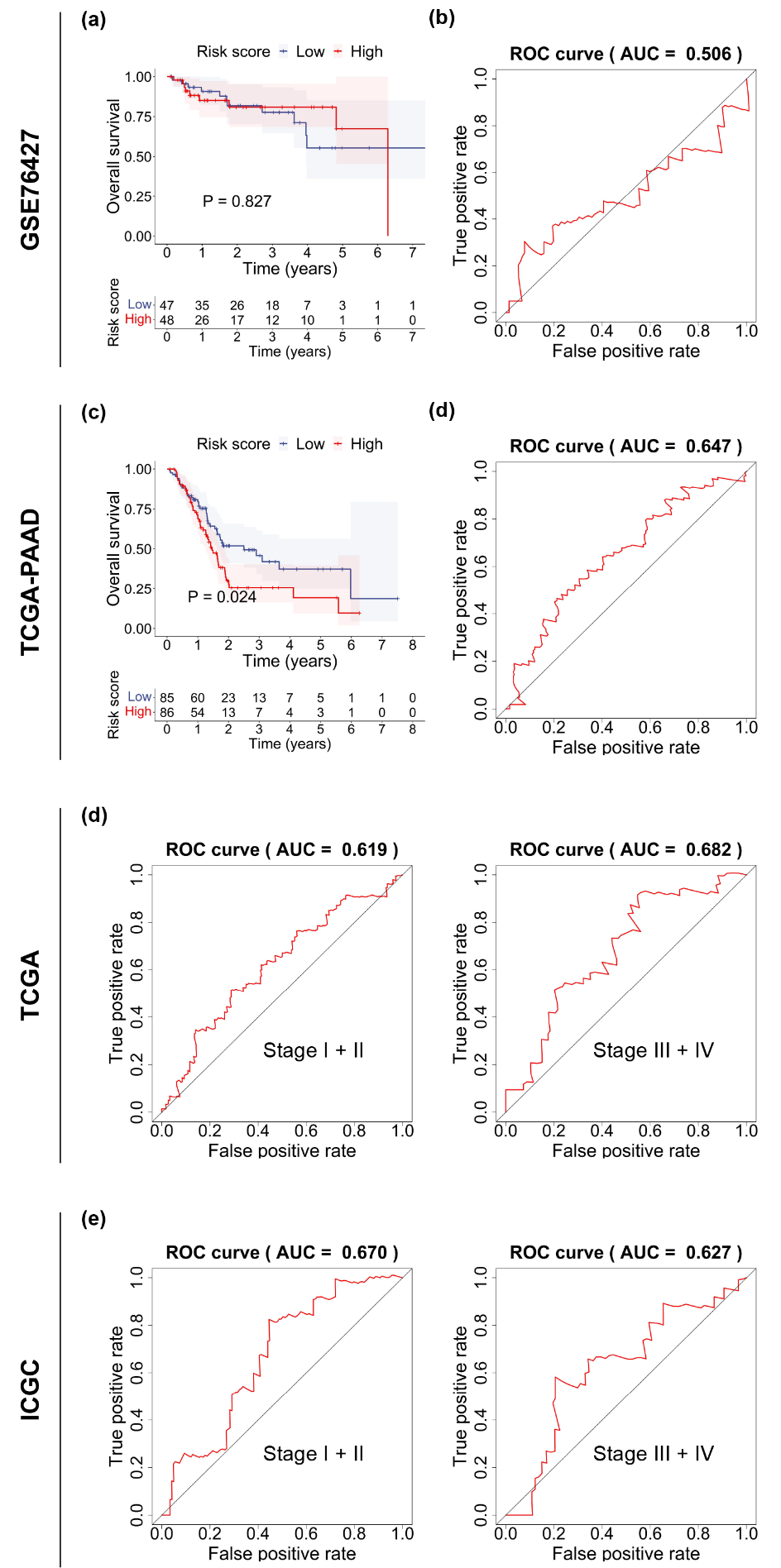


Figure S3: (a-d) KM curves and ROC curves of the risk model in the GSE76427 dataset (a-b) and the TCGA-PAAD cohort (c-d). (e-f) ROC curves of the risk model for different stages patients in the TCGA cohort (e) and the ICGC cohort (f).


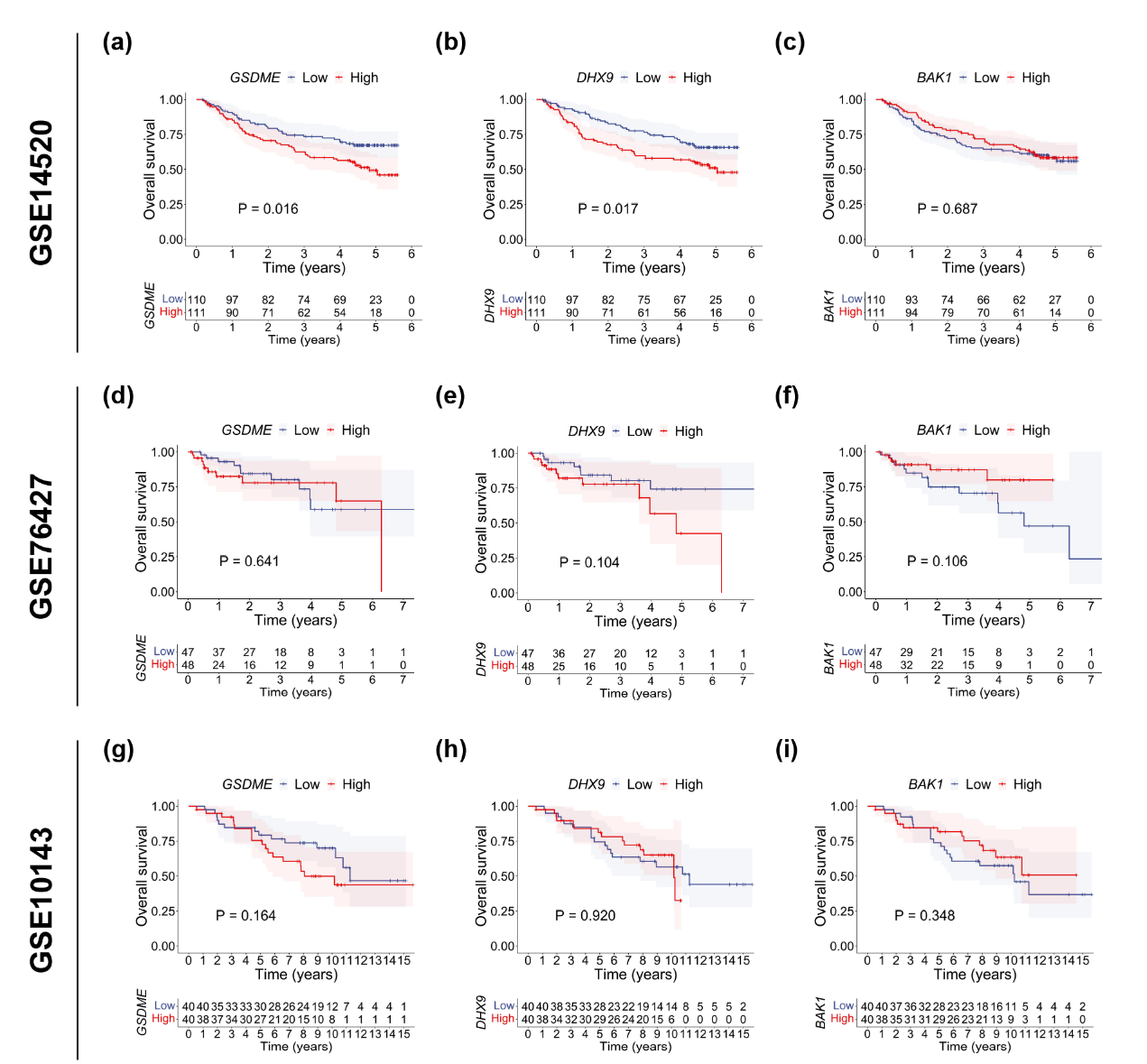


Figure S4: KM curves of *GSDME*, *BAK1*, and *DHX9* in the GSE14520 (a-c), GSE76427 (d-f), and GSE10143 (g-i) datasets.
