# Supplementary material for: Identification and Validation of Two Heterogeneous Molecular Subtypes and a Prognosis Predictive Model for Hepatocellular Carcinoma Based on Pyroptosis
Source: Oxid Med Cell Longev. 2022 Aug 28;2022:8346816. doi: 10.1155/2022/8346816 (PMC9441383; doi:10.1155/2022/8346816)
Supplement: Supplementary Materials — Figure S1: (a–c) KM curves indicating the differences of DSS, PFS, and DFS between the two pyroptosis subtypes in TCGA cohort. Figure S2: (a–c) KM curves showing the prognosis discrepancies of DSS, PFS, and DFS between the low- and high-risk groups in TCGA cohort. (d–f) ROC curves of the risk model in predicting patients' DSS, PFS, and DFS in TCGA cohort. Figure S3: (a–d) KM curves and ROC curves of the risk model in the GSE76427 dataset (a, b) and the TCGA-PAAD cohort (c, d). (e, f) ROC curves of the risk model for different stages of patients in TCGA cohort (e) and the ICGC cohort (f). Figure S4: KM curves of GSDME, BAK1, and DHX9 in the GSE14520 (a–c), GSE76427 (d–f), and GSE10143 (g–i) datasets. Table S1: primer sequences applied in the qRT–PCR experiment. Table S2: differential expression analysis and log-rank test results of the 40 PRGs in TCGA cohort. Table S3: differentially expressed genes (DEGs) between the two subtypes in TCGA cohort. Table S4: Differentially expressed genes (DEGs) between the two subtypes in the ICGC cohort. Table S5: coefficients of the six genes selected by the elastic net algorithm in TCGA cohort. [file 8346816.f1.zip › Table S1 (2).pdf]

Table S1. Primer sequences applied in the qRT–PCR experiment.

Genes	Primers (5’-3’)
β-actin-F	GTTGCTATCCAGGCTGTGCT
β-actin-R	AGTCCATCACGATGCCAGTG
BAK1-F	CAGGAACAGGAGGCTGAAGG
BAK1-R	ATAGGCATTCTCTGCCGTGG
CHMP4A-F	GGCCTATGGGCTTTGGAGAT
CHMP4A-R	AGGATACCCACTCAGCCAAC
CHMP4B-F	CGCAGATCGACGGCACATTA
CHMP4B-R	GTGTTGGTGTTGGCATTCTCC
DHX9-F	CGGTATGGAGATGGTCCACG
DHX9-R	CGCTACCATAGCCTCCACTG
GSDME-F	AAGGAATGAGGTCCTGTGCG
GSDME-R	CCACCACACTTCTCCTCGAC

F, forward; R, reverse.
