# Supplementary material for: Identification and Validation of Two Heterogeneous Molecular Subtypes and a Prognosis Predictive Model for Hepatocellular Carcinoma Based on Pyroptosis
Source: Oxid Med Cell Longev. 2022 Aug 28;2022:8346816. doi: 10.1155/2022/8346816 (PMC9441383; doi:10.1155/2022/8346816)
Supplement: Supplementary Materials — Figure S1: (a–c) KM curves indicating the differences of DSS, PFS, and DFS between the two pyroptosis subtypes in TCGA cohort. Figure S2: (a–c) KM curves showing the prognosis discrepancies of DSS, PFS, and DFS between the low- and high-risk groups in TCGA cohort. (d–f) ROC curves of the risk model in predicting patients' DSS, PFS, and DFS in TCGA cohort. Figure S3: (a–d) KM curves and ROC curves of the risk model in the GSE76427 dataset (a, b) and the TCGA-PAAD cohort (c, d). (e, f) ROC curves of the risk model for different stages of patients in TCGA cohort (e) and the ICGC cohort (f). Figure S4: KM curves of GSDME, BAK1, and DHX9 in the GSE14520 (a–c), GSE76427 (d–f), and GSE10143 (g–i) datasets. Table S1: primer sequences applied in the qRT–PCR experiment. Table S2: differential expression analysis and log-rank test results of the 40 PRGs in TCGA cohort. Table S3: differentially expressed genes (DEGs) between the two subtypes in TCGA cohort. Table S4: Differentially expressed genes (DEGs) between the two subtypes in the ICGC cohort. Table S5: coefficients of the six genes selected by the elastic net algorithm in TCGA cohort. [file 8346816.f1.zip › Table S2 (2).pdf]

Table S2. Differential expression analysis and log-rank test results of the 40 PRGs in the TCGA cohort.

Genes	logFC	Wilcox *P*	Log-rank *P*
CHMP4B	1.009	<0.001	0.001
CHMP4A	0.848	<0.001	0.001
GSDME	1.544	<0.001	0.002
BAK1	1.744	<0.001	0.002
IL1A	1.359	0.004	0.005
CHMP2B	0.560	<0.001	0.011
DHX9	1.152	<0.001	0.013
CHMP3	1.163	<0.001	0.015
APIP	1.280	<0.001	0.018
CASP4	0.396	0.008	0.026
CASP3	1.141	<0.001	0.026
BAX	1.632	<0.001	0.031
NLRC4	-0.060	0.236	0.016
GZMA	0.452	0.775	0.046
IL1B	-0.903	<0.001	0.054
IL18	0.672	0.833	0.075
GSDMC	4.995	<0.001	0.081
GSDMD	1.360	<0.001	0.105
CHMP6	0.997	<0.001	0.114
CASP8	1.165	<0.001	0.116
GSDMA	1.011	0.039	0.118
CHMP7	0.454	<0.001	0.138
IRF2	0.355	0.003	0.192
CASP5	1.124	0.103	0.195
HMGB1	0.617	<0.001	0.196
GZMB	0.649	0.315	0.225
CYCS	1.108	<0.001	0.235
NAIP	0.314	0.457	0.242
NLRP9	1.472	0.012	0.249
NLRP1	1.489	<0.001	0.340
ZBP1	0.390	0.445	0.357
CHMP4C	1.365	<0.001	0.362
GSDMB	1.462	<0.001	0.445
TP63	2.103	0.001	0.478
ELANE	0.112	0.048	0.535
CASP1	0.299	0.344	0.660
IRF1	0.253	0.091	0.756
TP53	0.874	<0.001	0.842
AIM2	0.801	0.030	0.919
CHMP2A	1.050	<0.001	0.950

logFC, $\log_2$(meanHCC – meanNormal).
