# Supplementary material for: Identification and Validation of Two Heterogeneous Molecular Subtypes and a Prognosis Predictive Model for Hepatocellular Carcinoma Based on Pyroptosis
Source: Oxid Med Cell Longev. 2022 Aug 28;2022:8346816. doi: 10.1155/2022/8346816 (PMC9441383; doi:10.1155/2022/8346816)
Supplement: Supplementary Materials — Figure S1: (a–c) KM curves indicating the differences of DSS, PFS, and DFS between the two pyroptosis subtypes in TCGA cohort. Figure S2: (a–c) KM curves showing the prognosis discrepancies of DSS, PFS, and DFS between the low- and high-risk groups in TCGA cohort. (d–f) ROC curves of the risk model in predicting patients' DSS, PFS, and DFS in TCGA cohort. Figure S3: (a–d) KM curves and ROC curves of the risk model in the GSE76427 dataset (a, b) and the TCGA-PAAD cohort (c, d). (e, f) ROC curves of the risk model for different stages of patients in TCGA cohort (e) and the ICGC cohort (f). Figure S4: KM curves of GSDME, BAK1, and DHX9 in the GSE14520 (a–c), GSE76427 (d–f), and GSE10143 (g–i) datasets. Table S1: primer sequences applied in the qRT–PCR experiment. Table S2: differential expression analysis and log-rank test results of the 40 PRGs in TCGA cohort. Table S3: differentially expressed genes (DEGs) between the two subtypes in TCGA cohort. Table S4: Differentially expressed genes (DEGs) between the two subtypes in the ICGC cohort. Table S5: coefficients of the six genes selected by the elastic net algorithm in TCGA cohort. [file 8346816.f1.zip › Table S3 (2).pdf]

Table S3. Differentially expressed genes (DEGs) between the two subtypes in the TCGA cohort. logFC, log2(meanPyHigh – meanPyLow); FDR, *P* values adjusted by false discovery rate.

Genes	logFC	Wilcox *P*	FDR
C16orf89	-3.37247761	1.93E-04	3.65E-04
PROK1	-3.15015099	1.94E-04	3.67E-04
HEPACAM	-2.86052905	1.00E-05	2.33E-05
INS-IGF2	-2.84501132	4.93E-05	1.03E-04
CYP1A1	-2.69305156	1.9101E-05	4.2452E-05
CD300LG	-2.6723949	1.04E-15	1.59E-14
RGSL1	-2.60023228	1.05E-08	4.05E-08
CYP3A4	-2.50346062	3.90E-10	1.92E-09
CYP1A2	-2.4529328	2.21E-07	6.77E-07
PMEL	-2.25766327	5.35E-04	9.40E-04
DRD1	-2.24616985	3.99E-11	2.36E-10
TMEM252	-2.13780498	5.5443E-05	0.00011457
HSD17B13	-2.09324654	4.92E-08	1.69E-07
GOLGA6B	-2.04029267	5.18E-06	1.2614E-05
RANBP3L	-2.03902932	6.70E-10	3.17E-09
LRCOL1	-1.96457939	1.30E-08	4.92E-08
C15orf43	-1.96297605	2.29E-06	5.95E-06
CA4	-1.87460216	1.87E-04	3.54E-04
CXorf66	-1.86130548	6.89E-07	1.95E-06
CYP8B1	-1.82663581	1.678E-16	3.1291E-15
UGT1A3	-1.82430564	6.60E-11	3.74E-10
GYS2	-1.82384864	1.66E-19	6.40E-18
MEP1B	-1.80487187	1.46E-04	2.81E-04
REN	-1.77978306	2.17E-08	7.90E-08
UGT1A4	-1.74949488	2.5209E-12	1.901E-11
SLCO1B7	-1.74535443	6.47E-05	1.32E-04
HPD	-1.70922562	8.81E-15	1.08E-13
GLS2	-1.70711259	1.78E-06	4.6976E-06
MROH2A	-1.68769927	1.00E-06	2.75E-06
ESR1	-1.68658252	1.01E-09	4.63E-09
RP11-545J16.1	-1.67810725	3.67E-04	6.62E-04
SLC22A2	-1.67758587	1.6008E-07	5.0159E-07
PTCHD4	-1.67598439	1.21E-06	3.26E-06
DSG1	-1.66352414	1.43E-10	7.56E-10
SRD5A2	-1.66064842	4.89E-10	2.37E-09
ZNF648	-1.65376484	2.53E-07	7.67E-07
SLC1A2	-1.65114455	1.32E-09	5.90E-09
OTOG	-1.6493603	1.89E-04	3.57E-04
ACE2	-1.64929103	2.2141E-05	4.8759E-05
HGFAC	-1.61961229	1.40E-06	3.75E-06
ABCA8	-1.60829131	1.39E-14	1.63E-13
CYP3A43	-1.60209335	3.26E-10	1.63E-09
HSD11B1	-1.59685844	1.52E-09	6.71E-09
MT1X	-1.59187367	3.76E-04	6.79E-04
ADRA1A	-1.58822817	4.5108E-09	1.8358E-08
SPDYC	-1.58731042	1.17E-11	7.69E-11
C1orf111	-1.58651026	3.69E-04	6.66E-04
ADH1B	-1.58634189	6.5911E-16	1.0578E-14
CTH	-1.58185701	2.07E-13	1.95E-12
ADH4	-1.58156114	3.09E-13	2.80E-12
PDK4	-1.57324329	6.90E-12	4.78E-11
AQP9	-1.55311604	2.84E-15	3.96E-14
TAT	-1.55259161	2.39E-15	3.37E-14
CFHR4	-1.54981242	2.75E-16	4.81E-15
ACSL6	-1.52039235	3.14E-05	6.74E-05
C18orf42	-1.50411447	1.30E-05	2.96E-05
MTFR2	1.50005294	1.13E-20	6.14E-19
GIP	1.50103242	4.55E-04	8.09E-04
SFXN3	1.50129796	7.43E-15	9.35E-14
C8orf88	1.50179754	1.70E-05	3.81E-05
CDT1	1.50197473	3.01E-20	1.41E-18
C12orf79	1.50402048	7.21E-11	4.05E-10
TEAD4	1.50412708	1.49E-11	9.62E-11
SLN	1.50518399	3.45E-05	7.38E-05
LIMK1	1.50548331	1.36E-18	4.19E-17
PCDHA7	1.50560509	6.24E-06	1.50E-05

**FLNA**	1.50591475	2.99E-06	7.58E-06
**PCDHB7**	1.50670485	4.28E-05	9.02E-05
**TNFRSF17**	1.50708421	0.00023728	0.00044212
**MAMSTR**	1.50724834	3.35E-16	5.73E-15
**FAM117B**	1.50774062	1.90E-12	1.46E-11
**BCAS4**	1.50855572	2.14E-22	2.00E-20
**FAM182B**	1.50856037	5.65E-09	2.26E-08
**MEP1A**	1.50876454	7.94E-06	1.88E-05
**CDC42BPG**	1.50881497	0.00049143	0.00086869
**PFN2**	1.50939741	3.16E-08	1.12E-07
**RNF186**	1.50946092	0.0003379	0.00061364
**CHST2**	1.51078959	1.49E-08	5.57E-08
**FOXS1**	1.51125739	2.18E-08	7.94E-08
**PRR15L**	1.5141297	2.10E-05	4.65E-05
**UCN2**	1.51419767	4.83E-07	1.40E-06
**TRNP1**	1.51439562	1.70E-15	2.47E-14
**STX1A**	1.51616967	1.80E-21	1.24E-19
**B3GALNT1**	1.51821496	5.42E-11	3.13E-10
**ASF1B**	1.51842323	1.71E-20	8.81E-19
**EMILIN2**	1.51873015	1.55E-15	2.28E-14
**GRB7**	1.51901427	5.22E-11	3.02E-10
**ORC6**	1.519082	2.10E-17	4.74E-16
**EMP3**	1.51958911	1.63E-14	1.88E-13
**COL16A1**	1.51972557	3.46E-09	1.43E-08
**CYBA**	1.51975047	1.78E-16	3.29E-15
**LAD1**	1.52005792	1.50E-09	6.62E-09
**KRTDAP**	1.52047401	3.85E-05	8.15E-05
**NRSN2**	1.52062047	8.66E-13	7.12E-12
**ZNF439**	1.52130347	5.40E-08	1.83E-07
**ZNF66**	1.52265086	6.01E-08	2.02E-07
**SLCO5A1**	1.52316135	1.33E-05	3.03E-05
**APLP1**	1.52454074	2.0425E-06	5.3435E-06
**MAGEA12**	1.52523146	5.58E-04	9.78E-04
**LDOC1**	1.52718546	4.16E-05	8.77E-05
**RAD54L**	1.5278372	2.52E-16	4.46E-15
**RAB6B**	1.52950099	7.77E-15	9.73E-14
**HPSE**	1.52957692	1.39E-11	9.0053E-11
**CMTM2**	1.53031098	2.39E-09	1.02E-08
**MAGEA3**	1.53083807	8.41E-05	1.68E-04
**PCDHA1**	1.53093311	8.30E-07	2.31E-06
**GLI1**	1.5311637	3.54E-12	2.60E-11
**GALNT6**	1.53123292	8.8089E-15	1.0833E-13
**SMIM6**	1.53166136	7.60E-09	2.97E-08
**KAL1**	1.53227247	2.96E-04	5.43E-04
**XKR6**	1.53248287	1.06E-06	2.90E-06
**TRIM45**	1.5335425	4.41E-11	2.58E-10
**TXLNB**	1.53464957	7.61E-07	2.13E-06
**FGD3**	1.53490317	5.45E-14	5.72E-13
**KCTD17**	1.5359002	8.70E-21	4.96E-19
**CYBB**	1.53632446	1.94E-10	1.00E-09
**TLR7**	1.53775205	2.50E-09	1.06E-08
**CD8B**	1.53816517	2.73E-07	8.23E-07
**SEMA4A**	1.53819212	1.80E-17	4.15E-16
**NAT8L**	1.53825766	1.04E-05	2.41E-05
**PCDHB10**	1.53852679	2.91E-05	6.28E-05
**TROAP**	1.53904826	2.31E-21	1.55E-19
**DIRAS1**	1.53922429	2.59E-07	7.83E-07
**SP140**	1.54007562	5.86E-10	2.79E-09
**APOBEC3C**	1.54124608	1.31E-11	8.57E-11
**CREB5**	1.54126447	1.25E-07	3.99E-07
**HAP1**	1.54152391	6.21E-06	1.49E-05
**FAM26F**	1.54275201	6.32E-11	3.60E-10
**CARD11**	1.54366757	5.83E-09	2.32E-08
**RIBC2**	1.54434906	4.03E-12	2.93E-11
**FAM57A**	1.54503822	3.02E-17	6.53E-16
**LMNB2**	1.54826193	1.32E-24	2.38E-22
**LGALS9**	1.54844042	7.38E-18	1.87E-16
**SLC7A7**	1.54875352	6.37E-13	5.42E-12
**UBE2U**	1.54887531	2.84E-05	6.13E-05
**CXCL14**	1.54891288	1.31E-04	2.54E-04

**TRPM2**	1.54918756	1.51E-18	4.60E-17
**ZDHHC1**	1.54928883	1.43E-07	4.50E-07
**CATSPER1**	1.54998776	2.29E-15	3.25E-14
**TMCC2**	1.55108882	1.19E-10	6.43E-10
**BCAS1**	1.55171414	3.53E-08	1.24E-07
**ANLN**	1.55267448	1.72E-12	1.34E-11
**XCL1**	1.55321644	1.22E-08	4.62E-08
**RNF17**	1.55332467	2.67E-05	5.80E-05
**PCNXL2**	1.55341413	5.90E-10	2.81E-09
**TTYH1**	1.55342473	5.18E-08	1.76E-07
**DBNDD2**	1.55767212	7.66E-12	5.24E-11
**GULP1**	1.55772259	1.73E-05	3.88E-05
**TEAD2**	1.55801567	1.83E-14	2.09E-13
**CDCA7**	1.55866184	1.81E-11	1.15E-10
**HOXA1**	1.56052648	2.12E-05	4.68E-05
**CDCA3**	1.56080801	1.09E-20	5.99E-19
**FCER1G**	1.56099729	1.13E-13	1.12E-12
**SLC5A5**	1.56216473	1.17E-05	2.70E-05
**NEURL1**	1.56227767	1.60E-09	7.06E-09
**B3GNT4**	1.56338366	7.76E-10	3.62E-09
**FJX1**	1.56399577	9.98E-06	2.32E-05
**TUBB2B**	1.56462149	1.57E-07	4.94E-07
**PLK1**	1.5650245	4.21E-19	1.4612E-17
**HCRT**	1.56544154	1.8726E-05	4.1686E-05
**CPNE7**	1.56582254	5.82E-06	1.41E-05
**IL2RA**	1.56607707	3.63E-12	2.66E-11
**COLCA2**	1.56657703	3.16E-13	2.86E-12
**FAM46B**	1.5670887	1.57E-07	4.93E-07
**DLX3**	1.56729456	8.09E-05	1.62E-04
**TPRXL**	1.56747576	5.21E-04	9.18E-04
**FABP5**	1.56790184	2.34E-11	1.45E-10
**ADAP1**	1.56891097	2.07E-11	1.30E-10
**CCDC183**	1.56920886	2.18E-12	1.66E-11
**GNB3**	1.56986991	5.89E-12	4.13E-11
**ACTA1**	1.57221325	2.71E-09	1.15E-08
**HAVCR2**	1.57246881	1.07E-12	8.66E-12
**CLECL1**	1.57294956	1.56E-13	1.50E-12
**GAS7**	1.57508397	8.29E-12	5.63E-11
**TPPP3**	1.57516337	1.20E-09	5.42E-09
**NCF2**	1.57543133	3.376E-15	4.6131E-14
**MMP2**	1.57549835	4.57E-05	9.56E-05
**FAM72D**	1.5755514	6.19E-18	1.60E-16
**PRR11**	1.57643559	2.05E-20	1.02E-18
**TACR2**	1.5764944	4.31E-07	1.26E-06
**GAGE1**	1.57659363	4.41E-06	1.09E-05
**RP11-392E22.9**	1.57679635	2.93E-04	5.37E-04
**NCAPH**	1.57725254	3.08E-17	6.65E-16
**LILRB4**	1.57750467	2.06E-12	1.57E-11
**SKA1**	1.578881	2.76E-17	6.03E-16
**HIST3H2BB**	1.57964956	5.85E-09	2.33E-08
**B4GALNT3**	1.57971298	8.63E-06	2.03E-05
**ARMCX2**	1.58251857	1.91E-05	4.25E-05
**KIF23**	1.58254486	4.20E-16	7.03E-15
**BIRC5**	1.58346533	1.88E-21	1.29E-19
**C4orf48**	1.58593874	3.19E-11	1.92E-10
**CLIC3**	1.58631235	1.42E-11	9.21E-11
**KIF18B**	1.58633572	2.15E-16	3.87E-15
**WDR62**	1.58722838	4.73E-22	3.96E-20
**PMAIP1**	1.58793411	2.16E-09	9.25E-09
**ZIC5**	1.58801821	3.20E-11	1.93E-10
**ACSS1**	1.58857535	2.22E-13	2.07E-12
**LRFN1**	1.58857567	7.0134E-12	4.8461E-11
**SLC38A1**	1.59085545	1.06E-10	5.77E-10
**C11orf70**	1.59106717	6.67E-09	2.62E-08
**TOR4A**	1.59206285	2.89E-12	2.16E-11
**HTRA3**	1.59276292	5.37E-09	2.15E-08
**DKKL1**	1.59277392	2.35E-09	1.00E-08
**SPECC1**	1.59296458	1.24E-04	2.42E-04
**CAPN9**	1.5932174	1.73E-08	6.41E-08
**CRTAM**	1.59507183	2.99E-06	7.58E-06

**TNFRSF21**	1.59651525	6.49E-14	6.70E-13
**FAM171B**	1.59793728	6.73E-07	1.90E-06
**CPA5**	1.59806391	7.37E-06	1.76E-05
**HIST3H2A**	1.59858572	2.92E-10	1.47E-09
**MCM10**	1.59888533	3.55E-16	6.03E-15
**PLP2**	1.59925879	2.88E-16	5.00E-15
**IGF2BP3**	1.59948397	1.55E-06	4.14E-06
**RAB34**	1.60085692	1.73E-09	7.58E-09
**EVC2**	1.60207884	1.37E-08	5.1579E-08
**NCK2**	1.60307614	2.27E-11	1.41E-10
**HLA-DQA2**	1.60339046	2.19E-08	7.98E-08
**GDPD3**	1.60411585	4.70E-12	3.37E-11
**KIAA1244**	1.6042844	1.35E-10	7.21E-10
**CERCAM**	1.60556475	7.30E-12	5.02E-11
**CTBP2**	1.60688703	5.52E-05	1.14E-04
**HILPDA**	1.6075709	8.80E-19	2.79E-17
**REEP2**	1.6098309	1.81E-15	2.63E-14
**ASPHD2**	1.61013965	7.10E-19	2.30E-17
**APCDD1**	1.61159169	1.89E-07	5.85E-07
**ENPP5**	1.6122067	3.45E-07	1.02E-06
**WDR54**	1.61267794	2.62E-19	9.70E-18
**NANOS3**	1.61349987	3.59E-11	2.14E-10
**FBXL2**	1.61497012	5.3434E-07	1.5393E-06
**ZDHHC13**	1.61585926	2.07E-12	1.58E-11
**PDZD7**	1.61587415	7.29E-17	1.46E-15
**AXDND1**	1.61649404	3.82E-08	1.33E-07
**NCS1**	1.61696812	4.95E-11	2.87E-10
**PIP5KL1**	1.61727322	5.16E-09	2.08E-08
**LRRC49**	1.61816884	1.22E-04	2.38E-04
**PPP2R2C**	1.61835653	1.73E-07	5.39E-07
**P3H4**	1.61879952	5.86E-13	5.01E-12
**DAND5**	1.62154274	1.34E-10	7.12E-10
**KIF12**	1.62178524	1.15E-10	6.2205E-10
**LIN28B**	1.6220748	2.20E-08	7.99E-08
**SERPINE2**	1.6222927	2.67E-08	9.57E-08
**CSF3R**	1.62294833	8.69E-12	5.88E-11
**SBK1**	1.62423051	1.18E-08	4.48E-08
**SELM**	1.62809805	3.19E-18	8.91E-17
**IKBKE**	1.62910753	6.43E-21	3.77E-19
**ZNF280B**	1.62919102	5.70E-05	1.17E-04
**CR1L**	1.63163014	1.35E-09	6.05E-09
**TESC**	1.63164965	3.47E-10	1.72E-09
**CCNI2**	1.63200763	2.26E-09	9.67E-09
**ZNF93**	1.63217693	7.42E-12	5.09E-11
**HDAC9**	1.63245264	6.77E-06	1.62E-05
**ANXA13**	1.63317988	2.17E-08	7.8958E-08
**KLHL35**	1.63357861	9.4823E-08	3.0869E-07
**MAPK13**	1.63426605	1.73E-12	1.35E-11
**HR**	1.63517946	4.89E-07	1.42E-06
**WDR66**	1.63582107	3.16E-09	1.32E-08
**FPR1**	1.63615384	9.2201E-10	4.2443E-09
**BSPRY**	1.63669124	9.57E-06	2.23E-05
**SYK**	1.63731476	9.50E-14	9.53E-13
**TAC3**	1.63738338	6.60E-05	1.34E-04
**RNF224**	1.63807566	1.41E-04	2.72E-04
**PTAFR**	1.64140679	1.62E-14	1.86E-13
**FAM101A**	1.64499577	9.61E-07	2.65E-06
**SV2A**	1.64720588	1.57E-09	6.93E-09
**KIAA0226L**	1.6473057	1.28E-10	6.84E-10
**IGLL5**	1.64896609	1.06E-04	2.08E-04
**ILDR1**	1.64981072	1.62E-08	6.03E-08
**C16orf74**	1.64993015	6.16E-10	2.93E-09
**IL2RG**	1.65015692	8.56E-14	8.67E-13
**TMEM54**	1.65164726	2.82E-10	1.42E-09
**HOXB7**	1.65314287	1.01E-06	2.76E-06
**RUNX2**	1.65319967	4.34E-06	1.07E-05
**P3H3**	1.65353509	7.91E-08	2.61E-07
**CLDN11**	1.65439359	1.37E-05	3.10E-05
**KLF5**	1.65480871	6.09E-07	1.74E-06
**ZNF239**	1.65844024	1.13E-11	7.50E-11

**ABCC1**	1.66428439	4.80E-11	2.79E-10
**NMB**	1.66558549	4.2499E-19	1.4701E-17
**GFPT2**	1.6657439	2.71E-07	8.16E-07
**FSCN1**	1.66588963	6.37E-09	2.52E-08
**NCF1**	1.66637853	2.68E-13	2.46E-12
**C5orf30**	1.66774391	2.63E-12	1.97E-11
**KRT15**	1.66916639	4.04E-09	1.66E-08
**AANAT**	1.67277594	1.16E-10	6.30E-10
**HAPLN3**	1.67376926	1.29E-14	1.53E-13
**ART5**	1.67419305	2.3342E-05	5.1172E-05
**FSCN2**	1.67436552	7.12E-10	3.34E-09
**CERS1**	1.67551307	1.02E-10	5.60E-10
**GCM1**	1.67697226	1.97E-04	3.72E-04
**S100P**	1.67812646	9.66E-07	2.66E-06
**GPX8**	1.67918271	4.11E-07	1.21E-06
**RGS1**	1.67973113	7.27E-13	6.09E-12
**WFDC3**	1.68013267	1.954E-11	1.2323E-10
**CCL13**	1.68259278	2.62E-05	5.69E-05
**SLC35E4**	1.682636	4.08E-19	1.42E-17
**SLC6A8**	1.68360653	1.28E-11	8.40E-11
**TNFRSF18**	1.68372528	3.45E-16	5.8788E-15
**ATP10B**	1.68403368	2.77E-04	5.11E-04
**SOWAHD**	1.68513936	6.61E-19	2.17E-17
**LEFTY1**	1.68530555	7.23E-08	2.40E-07
**GTSE1**	1.68532239	3.97E-18	1.09E-16
**CELF4**	1.68651443	1.18E-07	3.7802E-07
**TCTN2**	1.68707346	3.09E-12	2.30E-11
**DRAXIN**	1.6875286	2.68E-05	5.82E-05
**TLDC2**	1.68754481	1.68E-15	2.45E-14
**PCDHA6**	1.68773203	2.43E-04	4.52E-04
**CKMT1B**	1.687964	3.90E-08	1.36E-07
**CTHRC1**	1.68922047	9.19E-12	6.21E-11
**PCDHA3**	1.69374031	4.74E-06	1.16E-05
**ADRA2A**	1.69439109	6.54E-05	1.33E-04
**LETM2**	1.69492729	1.47E-09	6.54E-09
**3-Sep**	1.69512911	5.87E-09	2.33E-08
**GPR56**	1.69521778	4.68E-05	9.78E-05
**HPDL**	1.69599715	2.75E-10	1.39E-09
**PCDHB9**	1.6970776	6.40E-05	1.31E-04
**FAM81A**	1.69736529	1.20E-10	6.47E-10
**FAM171A2**	1.69788714	3.49E-10	1.73E-09
**MMP11**	1.69820686	3.48E-13	3.11E-12
**SMOX**	1.69843621	1.21E-21	8.68E-20
**KHDC1**	1.70119924	2.21E-10	1.13E-09
**SLC25A24**	1.70157711	2.87E-07	8.60E-07
**PPP2R3A**	1.70177068	7.76E-11	4.3322E-10
**ADAMTS14**	1.70382455	4.24E-11	2.49E-10
**TNFRSF13B**	1.70486391	6.24E-06	1.50E-05
**CARD14**	1.70542925	2.84E-11	1.73E-10
**FOXD4**	1.7064816	5.09E-07	1.47E-06
**IMPDH1**	1.70674206	2.60E-17	5.72E-16
**DUSP4**	1.70741389	3.84E-11	2.27E-10
**C17orf64**	1.70861592	2.82E-06	7.21E-06
**CENPA**	1.70895784	7.56E-21	4.39E-19
**LPCAT4**	1.70977804	2.66E-18	7.53E-17
**CCDC8**	1.71173997	4.3468E-05	9.1411E-05
**SIGLEC10**	1.71261437	1.3761E-12	1.0888E-11
**CEACAM3**	1.71294161	1.05E-06	2.86E-06
**APOBEC2**	1.71369133	8.2468E-05	0.00016514
**ZNF880**	1.7139456	6.72E-10	3.17E-09
**DPEP1**	1.71453103	4.17E-07	1.22E-06
**SHCBP1**	1.71757951	1.91E-15	2.76E-14
**CYP26B1**	1.71757967	4.56E-05	9.56E-05
**MIXL1**	1.71820523	8.88E-11	4.93E-10
**LIPH**	1.71831256	1.56E-06	4.15E-06
**OLFML2B**	1.71856273	3.95E-09	1.62E-08
**NT5DC2**	1.7205256	5.85E-14	6.10E-13
**ZBED2**	1.72203944	3.32E-11	2.00E-10
**CDH9**	1.72215544	4.37E-04	7.78E-04
**GGT6**	1.72274883	1.99E-04	3.75E-04

**CBX2**	1.72291952	6.66E-11	3.77E-10
**VCX**	1.7232706	5.55E-04	9.74E-04
**TMEM190**	1.72399066	5.02E-06	1.23E-05
**RAB27B**	1.72399569	2.80E-06	7.16E-06
**HS3ST1**	1.72471255	3.0121E-05	6.4893E-05
**EREG**	1.72526753	1.10E-04	2.15E-04
**TREM1**	1.72599656	2.54E-12	1.91E-11
**STYK1**	1.72637429	4.23E-06	1.05E-05
**ALOX5**	1.72661037	1.44E-08	5.40E-08
**BDKRB1**	1.72738748	9.17E-06	2.15E-05
**GCSAM**	1.7285339	4.62E-09	1.88E-08
**BCL2L15**	1.72881471	1.35E-05	3.06E-05
**ARL4C**	1.72904122	1.13E-14	1.35E-13
**IL21R**	1.72911887	1.24E-10	6.65E-10
**PKIB**	1.72956224	2.87E-09	1.21E-08
**BAI2**	1.72970608	5.40E-09	2.17E-08
**MCOLN2**	1.73014461	1.9404E-09	8.409E-09
**EMILIN3**	1.73022211	2.75E-09	1.16E-08
**PAQR5**	1.73224906	1.64E-08	6.1049E-08
**EPO**	1.73260326	3.46E-06	8.68E-06
**SHISA8**	1.73352513	1.08E-04	2.13E-04
**EPCAM**	1.73420062	5.36E-07	1.54E-06
**CPSF4L**	1.73475283	5.13E-08	1.75E-07
**MRC2**	1.73484648	1.29E-08	4.89E-08
**GAREML**	1.73599695	1.28E-14	1.51E-13
**EPHX4**	1.73613905	3.06E-10	1.53E-09
**CLEC11A**	1.73692207	1.60E-08	5.96E-08
**IL1B**	1.73717278	4.55E-10	2.21E-09
**CCNJL**	1.737429	8.96E-10	4.14E-09
**NAALADL1**	1.73842983	5.37E-08	1.82E-07
**CILP2**	1.73972143	3.25E-06	8.19E-06
**GRID2IP**	1.74008839	2.5708E-06	6.6049E-06
**PLA2G2D**	1.74094145	4.06E-06	1.01E-05
**SEMA3C**	1.74111665	1.78E-06	4.70E-06
**GPR161**	1.74251226	2.5672E-09	1.0886E-08
**CTB-102L5.4**	1.74252529	4.88E-04	8.63E-04
**HAGHL**	1.74369841	3.35E-12	2.47E-11
**GGTLC1**	1.74591238	8.55E-05	1.71E-04
**MMP14**	1.74743874	2.23E-10	1.14E-09
**TRIP13**	1.74976799	7.79E-20	3.23E-18
**CENPM**	1.75020593	3.18E-25	6.97E-23
**DUSP26**	1.75052874	3.61E-05	7.69E-05
**B3GNT8**	1.75127902	7.92E-14	8.07E-13
**DNAJB13**	1.75129394	4.58E-08	1.58E-07
**OSM**	1.75367485	2.43E-11	1.5018E-10
**AMH**	1.75437063	7.75E-06	1.84E-05
**EN2**	1.7553714	3.79E-05	8.04E-05
**CDC20**	1.75547296	3.27E-23	3.77E-21
**SOX9**	1.75631021	2.13E-14	2.3953E-13
**IGLL1**	1.75699547	2.53E-07	7.67E-07
**P2RY6**	1.75712875	8.95E-13	7.35E-12
**AFP**	1.75728917	3.89E-09	1.60E-08
**FGFR1**	1.76043207	4.30E-05	9.06E-05
**MITF**	1.76243343	5.65E-04	9.90E-04
**EMR1**	1.76344427	1.8526E-05	4.1291E-05
**CCDC185**	1.76401613	5.68E-07	1.63E-06
**IZUMO1R**	1.76671882	5.65E-04	9.90E-04
**MEX3A**	1.76867447	9.7228E-15	1.1798E-13
**PTCHD2**	1.76905329	2.3333E-08	8.4513E-08
**CKMT1A**	1.76978512	4.47E-09	1.82E-08
**ZNF296**	1.7701532	2.43E-20	1.18E-18
**CASP5**	1.7702921	3.02E-06	7.66E-06
**KIF2C**	1.77073346	3.35E-22	2.92E-20
**DSG2**	1.77129554	2.41E-11	1.49E-10
**RGS20**	1.77173462	3.24E-10	1.62E-09
**CHRNB2**	1.77253464	1.73E-05	3.86E-05
**DDX43**	1.77253912	9.63E-05	1.91E-04
**JSRP1**	1.77326295	2.02E-11	1.27E-10
**FAM90A1**	1.77416982	4.29E-12	3.10E-11
**HOXC6**	1.77464825	2.56E-05	5.58E-05

**KCNQ3**	1.77545445	6.29E-05	1.29E-04
**ZNF695**	1.77563687	5.58E-08	1.89E-07
**SEMA3A**	1.77697499	1.22E-05	2.81E-05
**GAL3ST4**	1.77945081	2.12E-11	1.33E-10
**C1orf145**	1.78163638	5.04E-08	1.72E-07
**CITED1**	1.78178251	2.85E-05	6.15E-05
**C1orf106**	1.78544685	8.10E-13	6.70E-12
**FAM129C**	1.7870945	5.23E-04	9.20E-04
**MORN3**	1.78757783	1.09E-08	4.17E-08
**PAPLN**	1.78781095	4.21E-11	2.48E-10
**CPA6**	1.7891999	0.00013351	0.00025891
**BNC2**	1.79037097	1.39E-04	2.69E-04
**DZIP1**	1.79116258	1.40E-06	3.77E-06
**TREM2**	1.79363421	1.95E-15	2.80E-14
**EFHC2**	1.79456466	1.07E-05	2.46E-05
**GAP43**	1.79830008	1.67E-06	4.42E-06
**SLC6A6**	1.79834386	1.07E-08	4.11E-08
**TNF**	1.79886616	8.57E-07	2.38E-06
**RNF175**	1.79895268	9.94E-07	2.73E-06
**TMC6**	1.79937601	1.13E-17	2.72E-16
**COL28A1**	1.80067179	2.80E-06	7.15E-06
**INSL3**	1.80179601	8.89E-15	1.09E-13
**CHST3**	1.80220885	5.10E-07	1.47E-06
**TRIM46**	1.80326712	3.46E-09	1.43E-08
**FBXO41**	1.81012958	3.09E-13	2.80E-12
**PTGES3L**	1.81274206	5.12E-16	8.4125E-15
**OTX1**	1.81293004	7.23E-12	4.98E-11
**CFAP45**	1.81590256	1.98E-17	4.50E-16
**TEX19**	1.81632532	3.34E-11	2.01E-10
**BPIFA2**	1.81771942	3.20E-08	1.13E-07
**TFAP2E**	1.81809644	6.04E-17	1.23E-15
**CREG2**	1.81879799	1.18E-09	5.35E-09
**CST2**	1.81923653	2.11E-06	5.51E-06
**GOLGA7B**	1.81957372	1.27E-09	5.72E-09
**MFSD10**	1.82167099	4.74E-26	1.45E-23
**CCL28**	1.82228525	1.89E-07	5.85E-07
**ACTL8**	1.82259698	1.50E-04	2.89E-04
**IGSF3**	1.82304492	1.45E-13	1.40E-12
**PODNL1**	1.82614854	1.87E-08	6.9072E-08
**IL4I1**	1.82651591	3.64E-19	1.29E-17
**UAP1L1**	1.82771089	1.73E-11	1.10E-10
**PLD4**	1.82782284	1.03E-10	5.64E-10
**ORAI2**	1.83006315	2.1002E-17	4.7438E-16
**ASCL5**	1.8307614	5.12E-08	1.75E-07
**SMARCD3**	1.8347731	1.46E-08	5.47E-08
**AURKB**	1.83484301	1.77E-22	1.67E-20
**GABRA3**	1.83506542	9.67E-06	2.25E-05
**TNFRSF11A**	1.83555543	1.34E-13	1.31E-12
**PDPN**	1.83649858	6.24E-06	1.50E-05
**ISYNA1**	1.84086817	3.82E-08	1.33E-07
**KLHDC8A**	1.8409093	1.48E-12	1.17E-11
**HOXC9**	1.84174221	7.04E-05	1.43E-04
**HNF1B**	1.84248423	4.30E-12	3.11E-11
**HID1**	1.84344607	9.38E-15	1.15E-13
**FCGR1B**	1.84561562	1.75E-13	1.66E-12
**FAM178B**	1.84858464	5.26E-09	2.11E-08
**RAB42**	1.8488959	1.42E-15	2.12E-14
**COLEC12**	1.84911339	4.69E-07	1.36E-06
**HOMER3**	1.85129456	1.07E-18	3.33E-17
**DLG3**	1.85307699	4.28E-11	2.51E-10
**FA2H**	1.85323874	1.48E-10	7.7917E-10
**SEMA6A**	1.85481837	3.38E-05	7.23E-05
**NFE2L3**	1.85538614	3.79E-16	6.41E-15
**B3GNT3**	1.8567372	1.64E-09	7.20E-09
**MS4A8**	1.85835836	3.48E-07	1.03E-06
**USP44**	1.85843437	7.1782E-05	0.00014531
**TMEM159**	1.85848952	2.11E-08	7.70E-08
**OLFML3**	1.85943674	1.63E-11	1.05E-10
**ALOX5AP**	1.86193161	3.8483E-13	3.4105E-12
**CD79A**	1.86215029	2.35E-04	4.38E-04

**MSC**	1.86288189	1.31E-06	3.53E-06
**NBL1**	1.86406993	2.01E-12	1.54E-11
**SLC16A3**	1.86554211	7.24E-20	3.05E-18
**CNKSR1**	1.87143519	3.08E-06	7.79E-06
**MST1R**	1.87225159	6.93E-07	1.95E-06
**FHOD3**	1.87355329	5.52E-07	1.59E-06
**SLC2A1**	1.87468665	2.46E-10	1.25E-09
**RSPH14**	1.87532402	2.43E-15	3.43E-14
**DUSP9**	1.87802142	9.76E-10	4.48E-09
**GBP5**	1.88179531	4.61E-06	1.13E-05
**KCNF1**	1.88290669	8.7733E-11	4.8722E-10
**PLA2G4F**	1.88356414	2.78E-05	6.03E-05
**PRR36**	1.8850706	3.53E-09	1.46E-08
**DLX6**	1.8867879	3.41E-05	7.28E-05
**UBE2C**	1.88728292	7.54E-22	5.80E-20
**LRP8**	1.88740096	8.12E-11	4.52E-10
**LARP6**	1.8900618	1.62E-09	7.11E-09
**PNMA2**	1.89052538	7.13E-05	1.44E-04
**EPHB6**	1.89219542	2.23E-07	6.82E-07
**VAV3**	1.89304675	4.10E-08	1.42E-07
**GPC2**	1.89341049	3.62E-12	2.65E-11
**C7orf61**	1.89342216	5.01E-11	2.91E-10
**GABBR1**	1.89464964	1.05E-04	2.07E-04
**SPP1**	1.89513273	1.06E-09	4.82E-09
**G6PD**	1.89531123	3.9922E-21	2.49E-19
**ADD2**	1.89551333	1.16E-06	3.14E-06
**OR8G5**	1.89725873	1.97E-04	3.72E-04
**C5orf46**	1.89814287	1.46E-08	5.47E-08
**TWIST1**	1.898432	3.32E-09	1.38E-08
**FHDC1**	1.89890557	5.88E-11	3.37E-10
**DEPDC1B**	1.89903414	2.07E-18	6.00E-17
**KLK1**	1.89947183	8.27E-09	3.22E-08
**CD22**	1.90632186	3.16E-06	7.97E-06
**RIPPLY2**	1.90702374	6.78E-06	1.62E-05
**IL18**	1.9094607	8.66E-13	7.12E-12
**SORCS1**	1.91098601	2.1284E-05	4.6954E-05
**M1AP**	1.91118817	5.83E-07	1.67E-06
**FFAR2**	1.91756833	9.40E-13	7.68E-12
**SLC35F2**	1.91815037	1.25E-10	6.70E-10
**MGAM**	1.9196783	2.58E-04	4.78E-04
**SPATA17**	1.92226233	4.34E-09	1.77E-08
**ZNF469**	1.92294669	9.72E-07	2.67E-06
**CXCL8**	1.92581362	4.46E-10	2.18E-09
**TNFRSF13C**	1.92774295	4.85E-10	2.35E-09
**TMEM63C**	1.92924325	2.70E-05	5.85E-05
**PAFAH1B3**	1.92953908	2.58E-26	8.67E-24
**TNNT2**	1.93108153	3.45E-09	1.43E-08
**GAPT**	1.93305053	6.842E-07	1.9346E-06
**WNT10A**	1.93313326	1.26E-14	1.49E-13
**DBN1**	1.93432498	8.65E-15	1.07E-13
**ANKLE1**	1.93497207	1.28E-09	5.73E-09
**S100A6**	1.93605511	2.25E-17	5.03E-16
**ELOVL4**	1.93794629	2.49E-07	7.56E-07
**CXCL3**	1.94618536	1.34E-12	1.06E-11
**PDIA2**	1.94761263	1.83E-05	4.08E-05
**BHLHE41**	1.95052814	2.46E-07	7.48E-07
**ALX1**	1.95337522	3.71E-04	6.70E-04
**TMPRSS3**	1.95418332	2.18E-07	6.664E-07
**TMSB10**	1.95465468	2.61E-27	1.13E-24
**KCNG1**	1.95580148	1.10E-05	2.54E-05
**UBASH3B**	1.95589029	4.15E-11	2.44E-10
**DDR1**	1.95708148	1.74E-11	1.11E-10
**CD24**	1.95813807	2.64E-19	9.76E-18
**FMN1**	1.95892228	5.62E-08	1.90E-07
**PLEKHH2**	1.95907779	6.16E-10	2.93E-09
**DDN**	1.95985376	5.56E-12	3.93E-11
**S100B**	1.96177022	1.82E-12	1.41E-11
**COL10A1**	1.96316821	2.42E-04	4.50E-04
**RGS17**	1.96458238	1.57E-08	5.85E-08
**QSOX1**	1.96674358	2.28E-10	1.17E-09

**NEFH**	1.97381021	4.14E-09	1.69E-08
**C3orf80**	1.97503368	9.22E-11	5.09E-10
**TYRO3**	1.9757137	2.5126E-20	1.2097E-18
**KCNK9**	1.97802382	1.77E-09	7.71E-09
**FZD7**	1.97826504	1.30E-11	8.51E-11
**GPC4**	1.97990168	8.45E-06	1.9905E-05
**HLA-DQB2**	1.98156033	4.74E-12	3.40E-11
**FXYD2**	1.98164749	7.24E-08	2.40E-07
**FAM24B**	1.98223252	4.791E-16	7.9253E-15
**NCAM1**	1.98379653	5.58E-04	9.78E-04
**SAPCD2**	1.9910845	3.89E-18	1.07E-16
**GLIS3**	1.99138151	3.7814E-07	1.1154E-06
**DYX1C1**	1.99173145	4.99E-07	1.44E-06
**INHA**	1.99296666	4.59E-04	8.15E-04
**NTS**	1.99576306	9.07E-05	1.81E-04
**FBLN1**	1.99723826	2.81E-09	1.18E-08
**S100A2**	1.99730165	3.69E-12	2.70E-11
**CCDC136**	1.99790362	5.23E-13	4.53E-12
**DUSP15**	1.99887687	1.67E-13	1.60E-12
**PIFO**	2.00037936	3.16E-11	1.91E-10
**CCDC74A**	2.00483462	2.82E-11	1.72E-10
**PLAU**	2.0051902	4.17E-10	2.04E-09
**ZNF860**	2.00573552	5.03E-05	1.05E-04
**KIAA1211**	2.00669234	1.12E-05	2.57E-05
**SCRN1**	2.00850965	5.14E-06	1.25E-05
**IL17RD**	2.00909305	1.45E-07	4.58E-07
**KIF5A**	2.00982522	3.31E-13	2.98E-12
**PCLO**	2.01176489	2.21E-08	8.01E-08
**PTPLA**	2.0120075	4.86E-07	1.41E-06
**SLMO1**	2.01289079	7.20E-10	3.38E-09
**SLFN13**	2.01351757	5.34E-07	1.54E-06
**LRRC36**	2.01406776	1.29E-08	4.87E-08
**DKK1**	2.01414097	7.77E-07	2.17E-06
**RUNDC3A**	2.01678664	1.94E-15	2.79E-14
**FUT4**	2.01681606	5.01E-12	3.57E-11
**CD70**	2.01765504	1.9956E-09	8.6189E-09
**ELF4**	2.02157839	4.44E-13	3.89E-12
**COMP**	2.02243599	2.78E-06	7.10E-06
**PLEKHG4**	2.02273139	1.06E-07	3.43E-07
**PFKFB3**	2.02456105	5.47E-07	1.5731E-06
**TMPRSS4**	2.02512211	3.93E-04	7.06E-04
**POPDC3**	2.02983111	1.14E-05	2.6225E-05
**SCUBE3**	2.03171811	2.17E-07	6.63E-07
**IGFBPL1**	2.03205518	0.00039079	0.0007028
**A4GNT**	2.03396942	3.60E-11	2.15E-10
**INPP5J**	2.03639724	3.58E-16	6.08E-15
**NPNT**	2.03777623	3.68E-04	6.65E-04
**GAD1**	2.03864109	2.07E-05	4.58E-05
**C19orf84**	2.0421078	2.23E-08	8.09E-08
**CADM3**	2.04269006	1.71E-04	3.27E-04
**FZD1**	2.04459371	4.48E-10	2.19E-09
**DZIP1L**	2.04873298	1.8089E-13	1.7167E-12
**MBOAT2**	2.05121088	2.91E-06	7.39E-06
**POU3F2**	2.05274598	5.64E-06	1.37E-05
**PTGES**	2.05460606	2.76E-09	1.16E-08
**CDR2L**	2.05624015	6.68E-09	2.63E-08
**FUT7**	2.05879618	1.32E-13	1.29E-12
**EVPL**	2.06070136	7.04E-08	2.34E-07
**NKAIN1**	2.06620651	1.0394E-10	5.6896E-10
**ARNT2**	2.06663133	1.70E-11	1.09E-10
**SLIT1**	2.06784693	1.55E-08	5.7771E-08
**RXFP4**	2.06959709	5.79E-09	2.31E-08
**TTC39A**	2.06977205	1.36E-13	1.33E-12
**MLLT3**	2.07050506	1.09E-09	4.98E-09
**OPRD1**	2.07085322	3.56E-08	1.25E-07
**C1orf186**	2.07441848	2.0714E-05	4.5813E-05
**SEC14L6**	2.07447676	1.52E-06	4.07E-06
**PRSS12**	2.07469604	5.14E-06	1.25E-05
**PPAP2C**	2.07485419	3.9289E-10	1.9352E-09
**RYR1**	2.07509899	8.06E-08	2.65E-07

**PLCD3**	2.07541556	2.25E-12	1.71E-11
**FANK1**	2.07677461	9.91E-12	6.65E-11
**GCNT1**	2.07705635	7.35E-09	2.88E-08
**ART3**	2.07771531	1.12E-05	2.58E-05
**PRAME**	2.0796153	7.90E-08	2.61E-07
**TRIM67**	2.07983374	1.17E-04	2.28E-04
**TLR10**	2.08123257	1.45E-07	4.57E-07
**PPFIA4**	2.08310167	1.84E-13	1.75E-12
**FCGR1A**	2.08426463	4.81E-15	6.30E-14
**LOXL1**	2.08427373	9.13E-05	1.82E-04
**BACE2**	2.08523532	3.04E-06	7.69E-06
**BEND6**	2.08591684	7.74E-05	1.56E-04
**RASAL1**	2.08833629	3.38E-11	2.03E-10
**SCIN**	2.09115873	0.00010399	0.00020491
**CYP2W1**	2.09320703	7.03E-07	1.98E-06
**IGSF11**	2.09737877	1.37E-06	3.67E-06
**ZNF486**	2.09777023	2.39E-07	7.27E-07
**KIF26B**	2.10099794	2.53E-06	6.50E-06
**TSPAN15**	2.1100371	1.74E-14	1.99E-13
**SOX4**	2.11030723	1.92E-18	5.66E-17
**FAM159A**	2.11161533	7.16E-16	1.14E-14
**PTPN13**	2.11228043	2.85E-04	5.23E-04
**CD19**	2.11229115	1.09E-07	3.50E-07
**EPHA10**	2.11272054	1.39E-06	3.72E-06
**DNM1**	2.11277068	1.10E-10	5.97E-10
**EGLN3**	2.11314796	2.65E-12	1.99E-11
**TMEM200C**	2.11356947	2.3836E-05	5.2185E-05
**ELOVL7**	2.11657649	9.97E-10	4.57E-09
**RNF183**	2.12379594	0.00028795	0.000529
**SMTNL2**	2.12411277	2.15E-07	6.59E-07
**IGDCC3**	2.12428208	5.18E-05	1.07E-04
**CLDN4**	2.12463355	3.42E-10	1.70E-09
**CT83**	2.12559408	1.59E-07	4.99E-07
**GAL3ST1**	2.12606826	9.79E-12	6.58E-11
**MBOAT4**	2.12636246	1.82E-08	6.732E-08
**PASD1**	2.12649582	2.41E-04	4.48E-04
**ERICH4**	2.12715725	4.20E-05	8.85E-05
**RETN**	2.12767296	7.78E-05	1.56E-04
**KHDC1L**	2.12949757	3.03E-05	6.52E-05
**HORMAD1**	2.12968539	4.19E-04	7.49E-04
**MCOLN3**	2.13009502	4.08E-08	1.42E-07
**STAP1**	2.13015661	2.65E-07	8.01E-07
**ADORA1**	2.13154007	3.32E-07	9.89E-07
**ADAMDEC1**	2.13329535	4.83E-08	1.66E-07
**CLMP**	2.13330818	1.95E-06	5.13E-06
**ARL9**	2.13331241	3.49E-08	1.23E-07
**C12orf56**	2.13426124	1.20E-06	3.26E-06
**CKM**	2.13947715	1.05E-09	4.81E-09
**LYPD6**	2.14100864	1.42E-07	4.47E-07
**DCDC2**	2.14206685	1.19E-06	3.22E-06
**PLBD1**	2.14255328	2.80E-12	2.10E-11
**TTLL6**	2.14901443	6.54E-07	1.85E-06
**PITX1**	2.14943913	5.27E-13	4.55E-12
**AIM2**	2.15351432	1.50E-07	4.71E-07
**SLC52A3**	2.15371218	2.52E-04	4.67E-04
**CCL26**	2.15392776	7.97E-13	6.62E-12
**DLX5**	2.15475159	9.95E-07	2.73E-06
**UGT8**	2.15581431	8.67E-07	2.40E-06
**TMEM35**	2.15609998	1.56E-05	3.51E-05
**LTB**	2.15691832	9.10E-13	7.46E-12
**PRSS16**	2.15774163	3.84E-07	1.13E-06
**GRIN2A**	2.15917127	6.32E-06	1.52E-05
**ITGB6**	2.16001052	5.56E-07	1.60E-06
**MLF1**	2.1603873	1.55E-10	8.15E-10
**C19orf33**	2.16325384	3.65E-10	1.80E-09
**CAGE1**	2.16490547	5.04E-08	1.72E-07
**PLAUR**	2.16763724	3.53E-20	1.61E-18
**ANKRD7**	2.17022787	1.73E-08	6.40E-08
**ADAM28**	2.1738557	1.29E-10	6.93E-10
**CEP55**	2.17531709	1.22E-22	1.18E-20

**14-Sep**	2.17591859	0.0003674	0.00066341
**HOXC4**	2.17632379	1.37E-06	3.69E-06
**WNT9A**	2.1793504	1.02E-04	0.00020165
**UNC13D**	2.17960353	6.05E-14	6.30E-13
**FBN2**	2.18637307	2.81E-06	7.16E-06
**ADORA2B**	2.18715804	4.91E-10	2.38E-09
**CLEC5A**	2.18905333	2.88E-12	2.16E-11
**MCEMP1**	2.18975398	1.23E-10	6.62E-10
**RIMKLA**	2.19120786	7.9338E-05	0.00015914
**ZNF90**	2.19180865	9.1769E-14	9.2307E-13
**DLX4**	2.19273378	2.38E-09	1.02E-08
**ANO4**	2.20129936	1.45E-06	3.89E-06
**TGFA**	2.20235126	2.67E-08	9.57E-08
**VANGL2**	2.20481612	4.03E-07	1.18E-06
**ZNF560**	2.20596181	8.18E-06	1.93E-05
**SEL1L3**	2.20969192	1.2757E-10	6.8355E-10
**SPINT2**	2.21259896	1.00E-06	2.74E-06
**CA9**	2.21393477	4.44E-14	4.72E-13
**MPP2**	2.2151235	4.43E-10	2.1628E-09
**MYBL2**	2.22026311	4.91E-27	1.84E-24
**BCAT1**	2.22736901	1.03E-11	6.87E-11
**BCL11A**	2.22905501	3.40E-09	1.41E-08
**IGDCC4**	2.22968779	7.73E-12	5.29E-11
**SLC4A11**	2.2313145	4.59E-13	4.01E-12
**CD7**	2.23219958	4.81E-12	3.44E-11
**TPBG**	2.23449683	6.19E-07	1.76E-06
**KCNQ1**	2.23732484	3.98E-06	9.88E-06
**FAM227A**	2.2411203	1.51E-12	1.19E-11
**SH2D3A**	2.24334459	6.8049E-13	5.7402E-12
**B3GNT7**	2.24429675	5.57E-10	2.67E-09
**ESRP1**	2.24599271	8.31E-06	1.96E-05
**HPCA**	2.2510385	9.04E-06	2.12E-05
**FXYD3**	2.25205696	1.15E-08	4.38E-08
**TMEM51**	2.25352523	3.91E-19	1.37E-17
**PF4V1**	2.25785904	3.80E-04	6.85E-04
**TUBAL3**	2.2621062	2.0252E-06	5.3027E-06
**CCDC64B**	2.26624683	1.9407E-07	5.9855E-07
**BICC1**	2.26625277	1.5776E-13	1.5173E-12
**CLIC6**	2.26766207	7.01E-07	1.98E-06
**S100A8**	2.27558422	1.91E-06	5.0192E-06
**MFI2**	2.27652645	7.72E-17	1.54E-15
**LIF**	2.27973553	6.66E-14	6.85E-13
**FAM64A**	2.28063027	9.51E-20	3.85E-18
**ARSI**	2.28314725	3.23E-06	8.14E-06
**RGS4**	2.28538004	4.50E-05	9.44E-05
**MMP9**	2.29203663	1.92E-17	4.39E-16
**ZNF488**	2.29264472	1.05E-08	4.03E-08
**CLEC17A**	2.29270466	1.17E-04	2.28E-04
**DUOXA1**	2.29543879	3.87E-05	8.19E-05
**CT45A10**	2.29574098	6.76E-05	1.37E-04
**SLC1A5**	2.29705301	2.35E-23	2.98E-21
**S100A11**	2.29914649	1.90E-20	9.59E-19
**GUCA2A**	2.29940396	9.92E-06	2.31E-05
**KAAG1**	2.30265137	1.09E-07	3.49E-07
**COL8A2**	2.30414494	2.50E-09	1.06E-08
**MMP1**	2.30579152	4.76E-08	1.64E-07
**NPTX2**	2.30623188	1.98E-04	0.00037381
**TMC4**	2.30703438	4.57E-04	8.11E-04
**S100A3**	2.30976881	3.25E-15	4.46E-14
**NCEH1**	2.31117105	2.89E-10	1.45E-09
**CTSE**	2.31395595	9.56E-08	3.11E-07
**ANKRD1**	2.3145685	2.62E-06	6.72E-06
**C1QL4**	2.31522481	3.48E-13	3.11E-12
**AQP10**	2.31937034	1.83E-04	3.47E-04
**KCNS1**	2.31960352	2.52E-06	6.48E-06
**PSORS1C1**	2.32038988	5.18E-08	1.76E-07
**ARHGEF38**	2.32257368	4.81E-05	1.00E-04
**IL20RA**	2.32323107	4.36E-08	1.51E-07
**TREX2**	2.32534794	3.1855E-08	1.1263E-07
**ISL2**	2.32595432	1.03E-12	8.36E-12

**FZD2**	2.32793955	1.85E-14	2.10E-13
**PRAC2**	2.32900344	1.14E-05	2.62E-05
**CTAG2**	2.32905204	1.07E-05	2.47E-05
**KEL**	2.33641048	1.19E-05	2.7381E-05
**SH2D5**	2.33673378	3.60E-08	1.26E-07
**POF1B**	2.3371939	4.67E-09	1.89E-08
**CTNND2**	2.33950835	2.25E-10	1.15E-09
**LMX1B**	2.34493164	4.09E-04	7.33E-04
**MYLK2**	2.34955631	1.54E-17	3.59E-16
**SHOX2**	2.35070879	8.17E-14	8.31E-13
**MAGEA11**	2.35415659	1.69E-05	3.7937E-05
**SEMA3E**	2.35424573	7.67E-11	4.29E-10
**FKBP10**	2.36106698	5.18E-12	3.67E-11
**KIF3C**	2.36965189	1.08E-11	7.16E-11
**DPF1**	2.36969224	1.56E-17	3.64E-16
**DCAF12L1**	2.37053432	3.89E-04	7.00E-04
**DSCAML1**	2.37362847	4.70E-04	0.00083339
**BANK1**	2.37495721	4.62E-04	8.20E-04
**ACPP**	2.37655114	2.08E-08	7.61E-08
**NLRP2**	2.37902562	4.8839E-05	0.00010185
**ANKRD18B**	2.38084143	2.68E-11	1.64E-10
**CEACAM6**	2.38829941	4.36E-05	9.16E-05
**MISP**	2.38883212	2.10E-10	1.08E-09
**ITPR3**	2.39340713	2.97E-08	1.05E-07
**UGT1A10**	2.40015196	4.37E-05	9.18E-05
**TFAP2A**	2.40266999	8.2043E-07	2.2864E-06
**MCIDAS**	2.40535301	4.17E-05	8.80E-05
**MDFI**	2.40971382	1.19E-10	6.41E-10
**GCNT3**	2.41008025	8.88E-09	3.44E-08
**CADPS**	2.41023582	9.38E-06	2.19E-05
**TRIM17**	2.41202042	4.01E-11	2.37E-10
**S100A9**	2.41259087	8.77E-10	4.05E-09
**HUNK**	2.41264401	5.60E-07	1.61E-06
**MTCL1**	2.41507619	7.84E-13	6.52E-12
**CDH6**	2.41590859	1.95E-07	6.02E-07
**PITX2**	2.42005482	1.08E-05	2.50E-05
**TRPA1**	2.42087472	9.82E-05	1.94E-04
**NXPH4**	2.42181761	1.49E-10	7.87E-10
**FRAS1**	2.42404255	2.51E-10	1.27E-09
**GJB3**	2.42826256	9.91E-07	2.72E-06
**TGFB2**	2.42931557	1.8413E-07	5.7054E-07
**MMP7**	2.4305128	1.05E-11	7.03E-11
**MFAP2**	2.43243438	7.58E-13	6.33E-12
**CRYGS**	2.43299297	1.88E-06	4.94E-06
**TCN1**	2.4349759	1.48E-07	4.67E-07
**SRRM3**	2.43844259	3.11E-06	7.85E-06
**FUT3**	2.44102706	3.54E-06	8.87E-06
**LPAR2**	2.4446507	2.55E-17	5.6309E-16
**ITGB4**	2.44497035	3.57E-12	2.62E-11
**CHIT1**	2.44609217	1.45E-04	0.00028023
**MACC1**	2.45167164	1.74E-07	5.41E-07
**TM4SF19**	2.45222651	1.62E-06	4.31E-06
**SPOCD1**	2.4629405	8.69E-11	4.83E-10
**ENO2**	2.46849166	3.1672E-15	4.3641E-14
**NKX2-5**	2.47029871	6.11E-05	1.25E-04
**PKIA**	2.4704056	4.9371E-14	5.2179E-13
**SRPX2**	2.47270093	5.78E-10	2.76E-09
**SSTR3**	2.4937663	4.14E-15	5.50E-14
**SOHLH2**	2.49477244	1.49E-06	3.98E-06
**C12orf75**	2.49862606	3.91E-12	2.84E-11
**RASGEF1A**	2.49888401	4.10E-10	2.01E-09
**HS3ST6**	2.49893927	7.93E-05	1.59E-04
**KRT17**	2.50250907	5.82E-10	2.78E-09
**EMR3**	2.50558672	1.12E-06	3.05E-06
**FOLR1**	2.50600695	3.01E-06	7.63E-06
**TMEM61**	2.5108485	2.2254E-11	1.3875E-10
**ANO9**	2.51214242	8.64E-14	8.74E-13
**NUDT11**	2.51224728	5.34E-05	1.11E-04
**HMX2**	2.51349324	4.83E-07	1.40E-06
**PFKP**	2.51723007	8.71E-10	4.03E-09

TFAP2C	2.51910275	4.36E-04	7.77E-04
TEKT2	2.5201471	8.19E-09	3.18E-08
CDH3	2.52123538	3.60E-07	1.07E-06
CRABP2	2.52697305	8.40E-07	2.34E-06
FIBCD1	2.52990776	1.03E-06	2.81E-06
NXNL2	2.53065793	3.23E-06	8.14E-06
KLHL30	2.5354252	1.47E-04	2.83E-04
DLX2	2.53791417	2.08E-08	7.60E-08
C6orf132	2.53796503	9.63E-09	3.71E-08
CNGB1	2.53924521	9.56E-12	6.43E-11
EPHB3	2.54188907	2.48E-11	1.53E-10
ERP27	2.54649627	1.63E-07	5.11E-07
PIWIL4	2.56584666	3.0231E-09	1.2657E-08
CHI3L2	2.57357378	4.87E-10	2.36E-09
RAB36	2.57388481	2.38E-09	1.01E-08
TNNT1	2.57508818	1.79E-11	1.14E-10
CXCL6	2.57724199	3.78E-07	1.11E-06
CST6	2.5881415	6.44E-10	3.0525E-09
CDC20B	2.59039391	1.69E-09	7.41E-09
SLC4A3	2.59150212	6.42E-08	2.15E-07
PQLC2L	2.59306874	1.23E-11	8.08E-11
TRPV6	2.59629276	2.88E-06	7.34E-06
ALPI	2.59645988	5.57E-04	9.76E-04
TMC5	2.59755019	1.57E-09	6.91E-09
TUBB3	2.59814073	1.34E-07	4.26E-07
STRA6	2.59968133	3.34E-10	1.67E-09
EYA1	2.60026847	8.83E-06	2.07E-05
FCGBP	2.60797019	4.98E-16	8.20E-15
PMEPA1	2.60801763	1.31E-07	4.15E-07
VWDE	2.61368093	6.22E-05	1.27E-04
SLC7A10	2.61610742	9.26E-08	3.02E-07
C2CD4C	2.61793386	9.30E-05	1.85E-04
SNAP25	2.61896875	2.96E-08	1.05E-07
PKM	2.61912467	3.76E-23	4.24E-21
CDCP1	2.62543015	5.13E-15	6.69E-14
CRLF1	2.63194991	1.15E-08	4.39E-08
C11orf86	2.63621594	5.29E-04	9.30E-04
BPIFB1	2.64482919	2.94E-08	1.05E-07
IL31RA	2.64558673	6.09E-05	1.25E-04
CPA4	2.64797765	2.19E-04	4.09E-04
TENM4	2.65393183	4.30E-05	9.05E-05
LRRC38	2.65450469	1.62E-05	3.64E-05
GPR84	2.65780286	1.39E-17	3.2888E-16
ARHGAP40	2.66562735	8.59E-09	3.33E-08
NEURL3	2.66769681	1.80E-19	6.89E-18
L1CAM	2.67604051	1.57E-05	3.53E-05
STEAP1B	2.67664979	7.05E-13	5.93E-12
TLL2	2.6796284	6.49E-16	1.05E-14
WNK2	2.68005469	2.66E-15	3.72E-14
LRRN1	2.68689677	1.36E-04	2.63E-04
NTRK1	2.68805767	1.31E-10	7.01E-10
ITGA3	2.69792596	2.04E-05	4.52E-05
NT5DC4	2.70284615	1.15E-15	1.75E-14
DES	2.70796567	7.33E-06	1.75E-05
SPTSSB	2.71494323	4.8147E-11	2.8004E-10
SLC35F3	2.72348396	1.22E-06	3.30E-06
KCNK2	2.72359739	9.8913E-06	2.3001E-05
CXCL1	2.73131935	5.49E-11	3.16E-10
CALCR	2.73233906	3.41E-09	1.42E-08
RCOR2	2.73426994	9.39E-13	7.68E-12
KCNH3	2.73891272	8.35E-09	3.25E-08
BMPR1B	2.74056164	6.85E-05	1.39E-04
TMEM72	2.74284035	3.48E-08	1.22E-07
NR0B1	2.74564737	7.91E-08	2.61E-07
FAM83B	2.74798827	4.60E-05	9.63E-05
PCYT1B	2.75347888	8.40E-06	1.98E-05
PLAC1	2.7541324	4.24E-11	2.49E-10
DUSP13	2.75525171	3.09E-09	1.29E-08
RP11-569G13.3	2.75604908	1.15E-06	3.12E-06
DMKN	2.76504975	5.37E-10	2.58E-09

**GPR1**	2.76696133	1.40E-04	2.70E-04
**DMBX1**	2.78505578	1.84E-09	8.00E-09
**FAXC**	2.78648468	1.41E-08	5.31E-08
**SLC6A17**	2.79276571	5.13E-06	1.25E-05
**EPS8L1**	2.79978899	6.32E-11	3.60E-10
**RBP2**	2.80693341	7.41E-05	1.49E-04
**GLIS1**	2.80693949	7.35E-07	2.066E-06
**CXorf67**	2.80854744	4.24E-05	8.93E-05
**SP8**	2.81180389	7.73E-08	2.56E-07
**FOXF2**	2.81348106	4.34E-09	1.77E-08
**SPINK13**	2.82455874	2.67E-07	8.07E-07
**SYNGR3**	2.82666897	1.04E-14	1.25E-13
**EFNA5**	2.82714232	1.87E-11	1.18E-10
**CAMKV**	2.8318307	4.64E-06	1.14E-05
**CYS1**	2.83354591	3.74E-05	7.94E-05
**ATP1A3**	2.83714252	3.7784E-08	1.3184E-07
**GIPR**	2.84366709	1.36E-12	1.08E-11
**HMGA2**	2.84416983	5.34E-10	2.57E-09
**MAGEA10**	2.85562963	4.67E-09	1.90E-08
**IAPP**	2.86620051	2.85E-05	6.16E-05
**TKTL1**	2.87139921	4.19E-06	1.04E-05
**MTNR1B**	2.87317203	0.00016923	0.000323
**SPINT1**	2.87866539	8.6374E-16	1.343E-14
**SPHK1**	2.87889685	3.05E-19	1.10E-17
**CTSV**	2.88516429	1.23E-11	8.10E-11
**CLPSL2**	2.89257503	5.75E-10	2.75E-09
**TFF1**	2.89310716	9.61E-06	2.24E-05
**KLC3**	2.91234655	1.96E-11	1.23E-10
**ITGB8**	2.92062856	1.57E-08	5.87E-08
**PVRL4**	2.9244012	1.3598E-06	3.6555E-06
**C1orf116**	2.92449676	2.40E-11	1.48E-10
**MAPK15**	2.93780655	1.46E-05	3.31E-05
**TDRD5**	2.94807562	2.203E-08	8.006E-08
**OVOL2**	2.94898259	6.34E-05	1.29E-04
**ZG16B**	2.9566394	1.09E-10	5.95E-10
**NMU**	2.97261184	8.40E-08	2.75E-07
**AC234582.1**	2.97479256	5.71E-06	1.38E-05
**GRHL2**	2.97617871	4.63E-06	1.1376E-05
**ZPLD1**	2.98710991	1.17E-10	6.31E-10
**MMP13**	2.98855702	3.11E-05	6.69E-05
**MMP12**	2.99301714	2.46E-09	1.0461E-08
**ST6GALNAC5**	2.99719669	3.13E-15	4.32E-14
**PPP1R14D**	3.00020121	5.05E-10	2.44E-09
**ATP2C2**	3.00146655	2.79E-06	7.12E-06
**COL9A2**	3.00896336	8.24E-21	4.75E-19
**DLX1**	3.00975573	3.97E-04	7.13E-04
**CAPN6**	3.01071086	9.5471E-10	4.3862E-09
**SLC5A1**	3.01425191	1.70E-04	3.25E-04
**SIX3**	3.02572152	3.20E-10	1.60E-09
**CTXN1**	3.03434914	1.55E-10	8.1466E-10
**RAB25**	3.03522632	4.6302E-05	9.69E-05
**B4GALNT4**	3.03721515	1.36E-08	5.11E-08
**NRG3**	3.04670367	1.89E-06	4.98E-06
**CHST4**	3.05025872	2.31E-07	7.05E-07
**SCG3**	3.05441624	7.90E-07	2.21E-06
**CR2**	3.05761876	3.95E-05	8.36E-05
**CDH10**	3.05920098	5.30E-06	1.29E-05
**GALNT5**	3.05950381	1.8598E-06	4.9006E-06
**SMIM22**	3.06130799	1.51E-13	1.46E-12
**PI3**	3.06243804	4.7012E-12	3.3745E-11
**OXTR**	3.07367988	1.5823E-09	6.9699E-09
**SAGE1**	3.10607922	1.8667E-07	5.7755E-07
**STAC2**	3.11159802	1.74E-08	6.45E-08
**CT45A1**	3.11199317	1.96E-06	5.15E-06
**TMSB15A**	3.11513488	6.16E-08	2.0674E-07
**BARX2**	3.12550201	5.90E-07	1.69E-06
**PRSS21**	3.13204759	9.01E-10	4.16E-09
**CLDN18**	3.14393099	6.65E-13	5.62E-12
**TMEM132A**	3.14579957	1.55E-16	2.93E-15
**RP11-1220K2.2**	3.14811381	6.07E-05	1.25E-04

**EPN3**	3.14858482	8.11E-10	3.77E-09
**HOXB13**	3.17655544	1.69E-06	4.4933E-06
**KRT80**	3.18003513	2.80E-11	1.71E-10
**PPAPDC1A**	3.18157701	2.93E-06	7.44E-06
**HOXC13**	3.18423382	2.01E-10	1.04E-09
**TMEM158**	3.19049179	1.39E-13	1.35E-12
**FOXJ1**	3.1987349	2.6033E-10	1.3185E-09
**KRTAP1-1**	3.1999751	1.46E-05	3.31E-05
**C2orf70**	3.20990744	1.82E-04	3.46E-04
**MUC1**	3.20996057	6.02E-08	2.03E-07
**GJA3**	3.21517933	5.59E-12	3.94E-11
**C3orf52**	3.22057317	3.44E-15	4.69E-14
**VWA5B2**	3.23260905	2.15E-11	1.34E-10
**LYPD6B**	3.23355739	3.92E-11	2.32E-10
**PPP1R1B**	3.23622474	1.37E-04	2.65E-04
**NELL1**	3.24180439	2.41E-04	4.49E-04
**CD207**	3.25718219	7.52E-07	2.11E-06
**GAST**	3.25965787	7.10E-06	1.69E-05
**FABP6**	3.26780422	2.00E-11	1.26E-10
**FTHL17**	3.26964298	4.21E-05	8.88E-05
**KRT19**	3.27407069	9.40E-10	4.32E-09
**C6orf222**	3.27929867	1.06E-06	2.8909E-06
**ARL14**	3.28455623	1.78E-06	4.70E-06
**KCNJ16**	3.30615551	3.21E-04	5.86E-04
**SUN3**	3.31203188	1.17E-05	2.69E-05
**UCHL1**	3.31511706	1.22E-07	3.90E-07
**HOXC11**	3.33201494	1.69E-04	3.22E-04
**FDCSP**	3.33309341	5.27E-10	2.54E-09
**MFSD6L**	3.33322302	1.07E-05	2.47E-05
**KIAA0319**	3.35261959	1.38E-05	3.13E-05
**PLEKHB1**	3.35743509	5.62E-16	9.17E-15
**PROM2**	3.36285531	7.22E-05	1.46E-04
**CD1A**	3.37534666	2.12E-10	1.09E-09
**IGFL2**	3.38109376	7.17E-12	4.94E-11
**C11orf53**	3.38200664	9.67E-05	1.92E-04
**ABCA12**	3.40286573	9.40E-08	3.06E-07
**CCDC74B**	3.41583195	3.656E-14	3.9605E-13
**SGPP2**	3.42849657	2.37E-11	1.47E-10
**PLEKHS1**	3.44076242	3.11E-07	9.30E-07
**DMRT2**	3.47142881	1.33E-07	4.22E-07
**ANXA8L1**	3.47831454	0.00015235	0.00029268
**COX6B2**	3.48016902	8.33E-08	2.74E-07
**CALB2**	3.49161741	3.09E-10	1.55E-09
**LAMA1**	3.49825618	8.28E-05	1.66E-04
**VCX2**	3.49878196	1.46E-04	2.82E-04
**COL11A1**	3.51213711	9.71E-07	2.67E-06
**NCCRP1**	3.52128408	9.77E-07	2.69E-06
**LAMP5**	3.52486167	7.93E-15	9.90E-14
**KIAA1549L**	3.53503391	1.1109E-08	4.2411E-08
**PKP3**	3.53849898	1.32E-08	4.97E-08
**HCN4**	3.54845599	1.55E-05	3.49E-05
**SOX21**	3.55320612	3.51E-04	6.36E-04
**PTHLH**	3.57836127	4.57E-13	4.00E-12
**INSM1**	3.5836993	7.88E-06	1.87E-05
**DSC3**	3.5859601	2.93E-04	5.37E-04
**CCDC160**	3.58655972	1.58E-04	3.03E-04
**SPEG**	3.6362321	5.00E-04	8.83E-04
**EPHA6**	3.63718235	7.09E-08	2.35E-07
**B3GALT5**	3.6525323	8.78E-08	2.87E-07
**SCD5**	3.66979894	9.75E-05	0.00019307
**CLEC2L**	3.67376281	3.28E-13	2.96E-12
**TNFAIP6**	3.67474633	4.06E-12	2.94E-11
**SIX2**	3.6902209	2.50E-07	7.58E-07
**NPPB**	3.69026959	2.08E-06	5.45E-06
**HOXD11**	3.73812943	3.35E-05	7.17E-05
**SLC28A3**	3.75182592	1.6399E-07	5.1267E-07
**IBSP**	3.75258079	4.58E-09	1.86E-08
**VCX3A**	3.76121776	8.55E-05	1.71E-04
**TMPRSS13**	3.76720103	1.87E-10	9.71E-10
**SHISA9**	3.76841957	1.61E-05	3.61E-05

**VEPH1**	3.77132649	4.8446E-11	2.8158E-10
**LAMC2**	3.79556624	1.2121E-05	2.7786E-05
**OPRK1**	3.84999413	5.23E-04	0.00092011
**FOXD1**	3.86407715	0.00012052	0.0002354
**CYP24A1**	3.87556296	3.57E-05	7.61E-05
**BIRC7**	3.87903684	6.90E-12	4.78E-11
**MMP10**	3.93200981	1.1453E-12	9.1907E-12
**TRIM72**	3.9329321	3.99E-10	1.96E-09
**ATP8A2**	3.93608882	2.50E-09	1.06E-08
**TEX15**	3.941372	3.69E-06	9.20E-06
**MYBPC2**	3.9428327	1.4213E-11	9.2212E-11
**LHFPL3**	3.95273768	4.53E-13	3.96E-12
**NPTX1**	3.95305606	4.30E-08	1.49E-07
**DLL3**	3.96549241	2.14E-10	1.10E-09
**SLC30A8**	3.9853843	2.12E-06	5.52E-06
**GPRIN2**	4.01881669	2.10E-09	9.04E-09
**MAGEA4**	4.0456883	1.18E-06	3.19E-06
**SYT13**	4.05211609	1.55E-10	8.15E-10
**CXCL5**	4.06597746	5.49E-08	1.86E-07
**RBBP8NL**	4.07836782	6.16E-05	1.26E-04
**ITIH5**	4.11713853	2.66E-05	5.77E-05
**NKAIN4**	4.14758996	3.08E-12	2.29E-11
**SBSN**	4.16920526	2.65E-08	9.49E-08
**TFF2**	4.19437818	4.89E-07	1.42E-06
**KRTAP4-1**	4.20038705	1.81E-05	4.03E-05
**PRSS22**	4.23181634	6.03E-08	2.03E-07
**SPANXB2**	4.24383461	2.7747E-06	7.0967E-06
**GPR115**	4.25224744	8.21E-11	4.5734E-10
**RHOV**	4.28432728	1.18E-14	1.40E-13
**SFRP5**	4.28816882	9.81E-08	3.19E-07
**WNT7B**	4.29390169	1.75E-09	7.65E-09
**CD177**	4.33469956	7.85E-12	5.36E-11
**SPIB**	4.34200549	2.81E-13	2.5667E-12
**HTR3A**	4.36426426	4.54E-06	1.12E-05
**SLC6A14**	4.36919797	7.16E-13	6.01E-12
**SLC6A15**	4.37146852	5.11E-04	9.02E-04
**HAPLN1**	4.37698518	1.4675E-05	3.318E-05
**APCDD1L**	4.40935833	2.26E-09	9.66E-09
**OLFM4**	4.45346379	1.14E-05	2.63E-05
**SCTR**	4.4565636	4.45E-04	7.93E-04
**PROM1**	4.46801939	1.83E-08	6.75E-08
**HOXB9**	4.51388336	1.47E-06	3.92E-06
**CUZD1**	4.53463965	3.56E-09	1.47E-08
**PPP1R14C**	4.65021747	1.17E-06	3.17E-06
**KRT4**	4.65270643	3.30E-05	7.09E-05
**TMEM130**	4.66697696	6.24E-06	1.50E-05
**MYO3A**	4.81372153	4.33E-07	1.26E-06
**MS4A15**	4.88764426	3.44E-04	6.25E-04
**SLC26A9**	4.95261703	2.98E-05	6.42E-05
**VTCN1**	4.99162475	2.4446E-07	7.4307E-07
**BPIFA1**	5.14726145	3.6666E-09	1.512E-08
**CRYBB1**	5.16345013	1.90E-09	8.25E-09
**SEZ6L**	5.3661992	1.77E-06	4.67E-06
**CHRNA1**	5.41216144	1.07E-05	2.48E-05
**MATN4**	5.45359404	1.13E-06	3.07E-06
**ALDH3B2**	5.5955813	1.86E-06	4.90E-06
**FAM216B**	5.62606004	1.39E-07	4.40E-07
**IL11**	5.68205256	4.05E-10	1.99E-09
**SLC34A2**	5.73997714	4.83E-07	1.40E-06
**FGF8**	5.95145238	4.10E-04	7.34E-04
**CACNG7**	6.09770561	5.35E-05	1.11E-04
**CLLU1OS**	6.13503163	4.57E-07	1.33E-06
**PAK7**	6.28625135	2.99E-07	8.96E-07
**CER1**	6.36634292	2.50E-04	4.65E-04
**XAGE2B**	6.43112603	5.83E-06	1.4101E-05
**DMBT1**	6.4689807	2.92E-08	1.04E-07
**CRYBA4**	6.57994152	3.40E-05	7.282E-05
**CLPSL1**	6.63475406	2.00E-04	3.78E-04
**RTL1**	7.08228122	3.73E-06	9.30E-06
**KIRREL2**	7.42335496	2.8608E-06	7.2886E-06

**CPA2**	7.51811217	1.73E-07	5.39E-07
**LGALS14**	7.62326127	4.3791E-06	1.0796E-05
**CEACAM7**	8.04278721	2.23E-04	4.17E-04
