# Supplementary material for: Identification and Validation of Two Heterogeneous Molecular Subtypes and a Prognosis Predictive Model for Hepatocellular Carcinoma Based on Pyroptosis
Source: Oxid Med Cell Longev. 2022 Aug 28;2022:8346816. doi: 10.1155/2022/8346816 (PMC9441383; doi:10.1155/2022/8346816)
Supplement: Supplementary Materials — Figure S1: (a–c) KM curves indicating the differences of DSS, PFS, and DFS between the two pyroptosis subtypes in TCGA cohort. Figure S2: (a–c) KM curves showing the prognosis discrepancies of DSS, PFS, and DFS between the low- and high-risk groups in TCGA cohort. (d–f) ROC curves of the risk model in predicting patients' DSS, PFS, and DFS in TCGA cohort. Figure S3: (a–d) KM curves and ROC curves of the risk model in the GSE76427 dataset (a, b) and the TCGA-PAAD cohort (c, d). (e, f) ROC curves of the risk model for different stages of patients in TCGA cohort (e) and the ICGC cohort (f). Figure S4: KM curves of GSDME, BAK1, and DHX9 in the GSE14520 (a–c), GSE76427 (d–f), and GSE10143 (g–i) datasets. Table S1: primer sequences applied in the qRT–PCR experiment. Table S2: differential expression analysis and log-rank test results of the 40 PRGs in TCGA cohort. Table S3: differentially expressed genes (DEGs) between the two subtypes in TCGA cohort. Table S4: Differentially expressed genes (DEGs) between the two subtypes in the ICGC cohort. Table S5: coefficients of the six genes selected by the elastic net algorithm in TCGA cohort. [file 8346816.f1.zip › Table S4 (2).pdf]

Table S4. Differentially expressed genes (DEGs) between the two subtypes in the ICGC cohort.
logFC, log2(meanPyHigh – meanPyLow); FDR, *P* values adjusted by false discovery rate.

Genes	logFC	Wilcox *P*	FDR
KCNU1	-3.73770974	2.26E-05	5.06E-05
RGSL1	-3.42511291	2.41E-10	1.81E-09
TKTL1	-3.26947383	2.50E-07	8.43E-07
SLC22A12	-3.26054397	1.12E-05	2.65E-05
RHBG	-2.94463495	1.28E-06	3.66E-06
CYP1A1	-2.89309991	4.52E-09	2.35E-08
DMRTC2	-2.78965745	4.17E-06	1.08E-05
SLC22A11	-2.74900197	7.89E-06	1.92E-05
C15orf43	-2.7294239	1.74E-11	1.93E-10
CYP3A4	-2.70180527	1.11E-10	9.27E-10
DRD1	-2.63703985	7.83E-05	1.59E-04
HGFAC	-2.48326934	6.28E-10	4.15E-09
PROL1	-2.40606861	6.40E-07	1.97E-06
DSG4	-2.36103819	1.07E-04	2.13E-04
CYP2E1	-2.36043996	4.99E-12	6.63E-11
DKK4	-2.35885405	1.72E-04	3.30E-04
TTC36	-2.28110733	7.40E-08	2.80E-07
TRPM3	-2.25524339	0.00029132	0.00053657
CFHR4	-2.23824255	4.72E-18	1.08E-15
CTH	-2.20782117	4.84E-12	6.48E-11
ZNF648	-2.18741433	9.79E-10	6.12E-09
NOTUM	-2.18594978	4.54E-07	1.44E-06
ALDH1L1	-2.07860059	4.84E-13	9.45E-12
SLC25A47	-2.07703432	9.16E-08	3.40E-07
APOC4	-2.06207608	3.85E-16	3.04E-14
HEPN1	-2.0438887	6.52E-06	1.61E-05
HSD11B1	-2.0224106	6.92E-09	3.42E-08
NECAB2	-2.01658947	7.89E-11	6.99E-10
TEX101	-2.01156596	5.38E-04	9.45E-04
HEPACAM	-1.9930235	4.83E-06	1.23E-05
CYP2C9	-1.99244963	2.61E-17	4.00E-15
APOA5	-1.96379036	4.13E-13	8.38E-12
TPPP2	-1.93662329	6.05E-11	5.59E-10
DSG1	-1.93102882	1.02E-11	1.22E-10
GALR3	-1.88472838	3.85E-06	9.99E-06
AQP9	-1.85783697	4.40E-15	2.18E-13
GOLGA6D	-1.84744064	5.55E-05	1.16E-04
C5orf27	-1.83997339	0.00041449	0.0007424
CYP2C8	-1.82581203	2.18E-10	1.65E-09
G6PC	-1.82463027	4.91E-12	6.56E-11
ADH1B	-1.81744864	1.01E-14	4.23E-13
CYP1A2	-1.81561898	1.15E-08	5.33E-08
CYP3A43	-1.81528228	9.81E-13	1.73E-11
AOX1	-1.79722148	1.54E-15	9.38E-14
CES5A	-1.78940537	2.90E-08	1.21E-07
HPD	-1.78232911	2.89E-12	4.25E-11
APOF	-1.76413715	1.26E-10	1.03E-09
SLC1A2	-1.75856242	2.03E-10	1.55E-09
ACSL6	-1.74377996	1.41E-07	5.04E-07
CA14	-1.7333976	4.26E-08	1.70E-07
GNMT	-1.70483163	5.13E-11	4.88E-10
WDR65	-1.68133167	1.06E-09	6.54E-09
SLC13A5	-1.67411899	1.03E-15	6.94E-14
CYP2A7	-1.66889542	5.07E-04	8.92E-04
TDGF1	-1.65298675	2.83E-09	1.56E-08
SLC13A3	-1.63605766	8.31E-06	2.02E-05
CYP2B6	-1.63123206	3.82E-08	1.55E-07
UBXN10	-1.61887605	5.08E-07	1.60E-06
TECTB	-1.61338948	1.26E-07	4.55E-07
SERPINC1	-1.59217725	5.28E-18	1.13E-15
CCDC38	-1.59053632	2.24E-11	2.38E-10
C9orf173	-1.58312022	9.44E-07	2.78E-06
PGLYRP2	-1.58290881	1.35E-10	1.10E-09
IAPP	-1.57856323	3.40E-11	3.42E-10
SLC22A10	-1.57588085	9.81E-10	6.12E-09
ADH4	-1.5485924	1.60E-11	1.80E-10

**CYP4A11**	-1.54109965	5.60E-14	1.64E-12
**G6PC2**	-1.53901947	1.48E-05	3.42E-05
**LINGO4**	-1.53643454	1.30E-08	5.97E-08
**RANBP3L**	-1.53312365	1.06E-07	3.89E-07
**SHBG**	-1.50453443	4.30E-09	2.24E-08
**TTPA**	-1.50156547	1.64E-12	2.65E-11
**GAPT**	1.50013978	1.43E-06	4.06E-06
**KIAA0408**	1.50015604	1.59E-08	7.14E-08
**SLC2A3**	1.50044951	1.23E-09	7.45E-09
**C19orf26**	1.50099396	3.00E-11	3.08E-10
**COL4A2**	1.50137181	4.56E-12	6.18E-11
**AKR1E2**	1.50224505	9.96E-07	2.92E-06
**IGFBPL1**	1.50270746	8.75E-06	2.11E-05
**NRM**	1.50320332	1.03E-15	6.94E-14
**FCGR1C**	1.5039028	5.06E-08	2.00E-07
**CLSTN1**	1.50437973	1.59E-14	6.06E-13
**GPR132**	1.50448219	3.73E-10	2.65E-09
**GRAMD1B**	1.50497776	4.96E-06	1.26E-05
**ABCA3**	1.50529293	3.28E-07	1.08E-06
**LAPTM5**	1.50756099	1.17E-11	1.37E-10
**MRVI1**	1.50801929	5.42E-09	2.75E-08
**DYNC2H1**	1.50814476	1.03E-08	4.82E-08
**ARMCX6**	1.50859586	5.41E-10	3.64E-09
**FPR1**	1.50955131	7.22E-08	2.75E-07
**LGALS2**	1.5099069	5.25E-06	1.32E-05
**SHCBP1**	1.51007542	1.02E-13	2.69E-12
**SYDE1**	1.51038133	5.30E-12	6.96E-11
**TLR2**	1.51129283	6.36E-11	5.83E-10
**RIPK3**	1.51148922	1.89E-09	1.09E-08
**CDC20**	1.51246252	5.12E-10	3.48E-09
**EZR**	1.51258941	4.27E-13	8.58E-12
**APOBEC3C**	1.51326845	1.47E-09	8.74E-09
**BGN**	1.51485385	1.02E-07	3.74E-07
**3-Mar**	1.51510196	5.00E-13	9.71E-12
**MMP10**	1.51519466	2.62E-10	1.95E-09
**PTRF**	1.51556871	1.54E-12	2.51E-11
**CCRL2**	1.51581386	1.61E-07	5.65E-07
**ARHGAP22**	1.51689749	9.90E-11	8.41E-10
**SRC**	1.51732824	1.06E-14	4.42E-13
**RGS1**	1.51744286	2.59E-09	1.44E-08
**GNB3**	1.51781182	8.57E-14	2.30E-12
**PKDCC**	1.51796744	4.34E-10	3.02E-09
**OVOL1**	1.51822904	3.21E-06	8.45E-06
**DKK2**	1.51852339	2.58E-06	6.93E-06
**CDX1**	1.51866876	1.38E-04	2.70E-04
**CD9**	1.5195076	1.15E-09	7.02E-09
**CAPN9**	1.51970307	1.47E-04	2.86E-04
**BANK1**	1.52094722	3.92E-06	1.02E-05
**MECOM**	1.52125115	1.91E-09	1.10E-08
**MDH1B**	1.5214653	2.45E-09	1.37E-08
**RAP1GAP**	1.52167612	8.07E-05	1.64E-04
**HAVCR2**	1.52189872	4.35E-12	5.95E-11
**MMEL1**	1.5222746	9.61E-15	4.10E-13
**DDN**	1.5226794	1.70E-08	7.56E-08
**SULF2**	1.52284987	1.47E-10	1.19E-09
**C1QTNF2**	1.5233644	3.50E-08	1.43E-07
**CECR6**	1.52348051	2.83E-09	1.56E-08
**GTSF1**	1.52527428	1.04E-04	2.08E-04
**PDE5A**	1.52641797	9.87E-09	4.66E-08
**ERC2**	1.52818375	5.66E-08	2.21E-07
**GNG8**	1.52818863	6.95E-08	2.66E-07
**RADIL**	1.52854552	2.33E-07	7.89E-07
**KLRC2**	1.52910476	9.99E-06	2.39E-05
**ACE**	1.52913096	2.42E-09	1.36E-08
**GALNT4**	1.52914745	2.73E-07	9.13E-07
**SLC4A5**	1.5296108	2.15E-13	4.94E-12
**DIRC3**	1.53039429	2.78E-08	1.16E-07
**ZNF391**	1.53204284	2.17E-06	5.93E-06
**SAMD15**	1.53234692	1.61E-09	9.52E-09
**EDNRA**	1.5329173	1.77E-11	1.95E-10

**LAIR1**	1.53350103	2.12E-10	1.61E-09
**FBXL13**	1.53403231	7.16E-12	8.94E-11
**RAB3D**	1.53406456	3.68E-08	1.50E-07
**IQCD**	1.53411132	1.49E-14	5.74E-13
**MB21D2**	1.53466675	9.30E-10	5.84E-09
**PAPPA**	1.53510105	6.56E-05	1.36E-04
**SMPD3**	1.5352684	4.76E-06	1.21E-05
**PNMAL1**	1.5354788	1.01E-07	3.71E-07
**HDAC7**	1.53639075	1.91E-14	6.90E-13
**CEACAM4**	1.53733949	1.32E-06	3.77E-06
**EVC**	1.53776766	9.00E-06	2.17E-05
**SH3RF3**	1.53794169	1.40E-05	3.26E-05
**PLCB4**	1.53837643	7.52E-06	1.84E-05
**UPP1**	1.53843021	2.56E-13	5.68E-12
**ITGAX**	1.538458	1.70E-09	9.95E-09
**NAALADL1**	1.53945415	2.35E-07	7.97E-07
**SLIT1**	1.53984499	2.60E-05	5.74E-05
**TMEM145**	1.54047481	2.25E-05	5.04E-05
**HDGFL1**	1.54173104	5.04E-04	8.87E-04
**ZBED2**	1.54186499	5.23E-06	1.32E-05
**POC1B-GALNT4**	1.54308562	6.80E-08	2.60E-07
**RPP25**	1.54379321	6.01E-11	5.56E-10
**C1QTNF1**	1.54581133	2.61E-07	8.75E-07
**GBGT1**	1.54632327	2.61E-13	5.75E-12
**PLXNA1**	1.54671686	1.01E-14	4.23E-13
**LMTK3**	1.54720786	4.29E-07	1.37E-06
**PRR19**	1.54786555	8.66E-13	1.56E-11
**NOTCH3**	1.54857804	8.71E-11	7.55E-10
**EFEMP2**	1.55030134	9.95E-12	1.19E-10
**PKMYT1**	1.55176364	2.72E-15	1.49E-13
**SLC38A1**	1.55218015	3.07E-12	4.48E-11
**LUM**	1.55262009	6.01E-07	1.86E-06
**PLA2R1**	1.55271029	1.10E-06	3.19E-06
**CATSPERG**	1.55363922	1.16E-08	5.39E-08
**ZNF320**	1.55410838	7.24E-11	6.52E-10
**SH3YL1**	1.55413373	3.38E-04	6.15E-04
**SOD3**	1.55566694	6.83E-09	3.38E-08
**VWDE**	1.55675246	5.43E-04	9.52E-04
**COX7A1**	1.55855101	4.31E-08	1.72E-07
**KCNQ5**	1.56009622	2.07E-05	4.67E-05
**NBEA**	1.56073696	1.24E-06	3.56E-06
**SH3BGRL3**	1.56104764	6.20E-16	4.58E-14
**PKNOX2**	1.5612241	6.77E-07	2.06E-06
**RGS2**	1.56146859	6.63E-10	4.36E-09
**STAR**	1.56195883	1.10E-04	2.19E-04
**SELM**	1.56220799	1.28E-11	1.48E-10
**BCAT1**	1.56221513	2.65E-13	5.82E-12
**CSF3R**	1.56295845	3.91E-08	1.58E-07
**PLXDC2**	1.56382762	3.63E-10	2.59E-09
**CORO2A**	1.56394298	1.02E-05	2.44E-05
**ACSS1**	1.56478857	2.72E-12	4.08E-11
**MYH11**	1.56490829	1.68E-07	5.89E-07
**PTPRO**	1.56548562	3.14E-11	3.20E-10
**C11orf45**	1.56553869	7.37E-09	3.61E-08
**PRAM1**	1.56645494	1.87E-08	8.24E-08
**ROR2**	1.56736247	2.96E-06	7.84E-06
**PHYHIP**	1.56788544	8.37E-09	4.04E-08
**GATA3**	1.56831225	2.95E-04	5.43E-04
**ATP1B3**	1.56867757	1.70E-18	5.57E-16
**HMGA1**	1.56908179	9.29E-15	4.01E-13
**IGLL5**	1.56962988	8.74E-05	1.77E-04
**CHRFAM7A**	1.56987789	2.65E-06	7.11E-06
**HS3ST6**	1.57099492	3.27E-04	5.96E-04
**SLC9A1**	1.57113503	1.07E-15	7.04E-14
**EFS**	1.57185078	3.31E-07	1.09E-06
**SLC39A2**	1.57231419	2.30E-05	5.14E-05
**AEBP1**	1.57260198	2.73E-07	9.13E-07
**SLIT2**	1.5733192	2.44E-06	6.58E-06
**CIB2**	1.57577765	4.13E-11	4.06E-10
**PYGB**	1.57602788	4.51E-19	2.17E-16

**CTSK**	1.57628495	2.10E-09	1.19E-08
**LIMK1**	1.57650552	3.85E-16	3.04E-14
**GSDMC**	1.57758615	7.33E-09	3.60E-08
**LRRC16A**	1.57830604	9.65E-13	1.71E-11
**C6orf223**	1.57903567	4.53E-09	2.35E-08
**APBA2**	1.57967608	2.33E-05	5.20E-05
**SCARF2**	1.58029725	1.99E-08	8.68E-08
**EHD2**	1.58156165	2.26E-13	5.12E-12
**CD52**	1.58194446	7.09E-07	2.15E-06
**SUSD5**	1.5820241	1.56E-06	4.39E-06
**SPINK2**	1.58305382	5.00E-04	8.81E-04
**KLC3**	1.58322938	1.26E-11	1.46E-10
**GRB7**	1.58419098	3.63E-07	1.18E-06
**PAQR4**	1.58466986	7.58E-15	3.37E-13
**KCNC4**	1.58470291	3.36E-12	4.81E-11
**TIMP4**	1.58522487	3.00E-06	7.95E-06
**KCNK6**	1.58524102	1.92E-08	8.41E-08
**ITGAM**	1.58592346	1.22E-12	2.07E-11
**CITED1**	1.58610119	1.46E-05	3.38E-05
**MFSD6**	1.58756694	7.64E-13	1.40E-11
**C10orf107**	1.58771163	1.19E-07	4.32E-07
**SGCA**	1.58859792	3.87E-05	8.31E-05
**TMCC2**	1.58862762	1.47E-10	1.19E-09
**FZD7**	1.58868521	2.24E-09	1.27E-08
**SLC4A9**	1.58893801	5.29E-08	2.08E-07
**KRBA1**	1.58894298	6.23E-13	1.18E-11
**NCF2**	1.58900057	5.71E-12	7.42E-11
**IQCA1**	1.58951141	2.75E-10	2.04E-09
**KCTD17**	1.58955801	6.13E-13	1.16E-11
**IKBKE**	1.58980647	2.11E-14	7.50E-13
**ASCL2**	1.59010029	7.76E-08	2.92E-07
**ZNF835**	1.5911665	2.12E-09	1.21E-08
**FOXC2**	1.59133058	3.13E-09	1.70E-08
**ARHGEF19**	1.59194398	2.05E-11	2.21E-10
**BVES**	1.59203119	9.99E-09	4.72E-08
**PDP1**	1.59281975	1.14E-09	6.94E-09
**SERPINI1**	1.59517608	4.81E-05	1.02E-04
**RHBDF1**	1.59528491	2.40E-12	3.67E-11
**LAT2**	1.59648952	6.74E-12	8.53E-11
**LTBP1**	1.59677167	2.27E-09	1.28E-08
**AP1S3**	1.59762879	1.92E-10	1.48E-09
**SHOX2**	1.59829795	3.18E-09	1.72E-08
**RNF24**	1.5986495	2.92E-17	4.39E-15
**BNC2**	1.59887025	1.04E-09	6.41E-09
**P2RX1**	1.59924049	1.38E-06	3.92E-06
**FAM163A**	1.60073287	5.84E-08	2.27E-07
**NRSN2**	1.60141082	1.65E-11	1.84E-10
**TRPM2**	1.60172474	1.04E-12	1.82E-11
**SAP25**	1.60362071	2.00E-07	6.89E-07
**SIM2**	1.60406444	1.01E-05	2.42E-05
**HTR1B**	1.60413983	1.60E-06	4.48E-06
**SLAMF8**	1.60547222	9.68E-10	6.05E-09
**PLD4**	1.60655031	5.87E-07	1.82E-06
**FBXO41**	1.6065863	1.50E-07	5.30E-07
**RNFT2**	1.60746916	1.98E-14	7.08E-13
**ATP8B3**	1.60767747	2.33E-04	4.37E-04
**ENDOD1**	1.6087978	4.59E-10	3.17E-09
**RAB9B**	1.60935298	6.96E-08	2.66E-07
**PAFAH1B3**	1.60966896	8.51E-16	5.98E-14
**TTLL9**	1.60997327	7.09E-05	0.00014565
**OXCT1**	1.61053326	4.30E-09	2.24E-08
**PPP1R13L**	1.61161427	3.58E-10	2.56E-09
**RUFY4**	1.61167243	1.79E-06	4.98E-06
**RGS11**	1.61229105	2.47E-04	4.60E-04
**DACT3**	1.61240746	2.49E-08	1.05E-07
**DPY19L2**	1.6129615	8.83E-07	2.62E-06
**CSTA**	1.61314705	1.59E-04	3.07E-04
**OSBPL10**	1.61388992	2.55E-09	1.42E-08
**ACRV1**	1.61414534	1.07E-06	3.11E-06
**PRPH2**	1.61415553	1.09E-08	5.09E-08

**HOXD1**	1.61474021	4.66E-06	1.19E-05
**HN1**	1.61633418	4.21E-18	1.01E-15
**DSEL**	1.61667842	1.09E-08	5.09E-08
**PROCR**	1.61753283	6.18E-11	5.69E-10
**HYAL4**	1.61798741	4.06E-04	7.29E-04
**ASNS**	1.61983544	1.39E-10	1.13E-09
**TUBA1A**	1.62083785	8.00E-13	1.45E-11
**MSRB3**	1.62208195	4.16E-12	5.75E-11
**ZSWIM4**	1.6222935	1.62E-11	1.82E-10
**RGMA**	1.62275026	1.75E-05	4.00E-05
**SLC25A24**	1.62382272	3.53E-14	1.14E-12
**MYL9**	1.62542702	4.44E-11	4.33E-10
**CENPM**	1.62636003	2.93E-12	4.31E-11
**ANXA4**	1.62672981	6.08E-06	1.51E-05
**PTAFR**	1.62721328	1.03E-10	8.72E-10
**MICAL1**	1.62780398	6.43E-13	1.21E-11
**RIMKLB**	1.62853962	2.49E-04	4.64E-04
**CRISPLD2**	1.62860283	5.20E-07	1.63E-06
**SYS1-DBNDD2**	1.62924077	4.01E-19	2.06E-16
**NOX4**	1.62939962	2.41E-13	5.36E-12
**CCL17**	1.63017462	7.16E-05	1.47E-04
**SPIRE1**	1.63028353	4.83E-09	2.48E-08
**PTGS1**	1.63046678	4.35E-12	5.95E-11
**MBOAT4**	1.63064839	8.78E-05	1.78E-04
**AFAP1**	1.63086101	2.75E-11	2.85E-10
**ADAMTS4**	1.63110131	3.58E-05	7.73E-05
**WDR86**	1.63224232	1.60E-04	3.09E-04
**LIMCH1**	1.6324496	1.09E-05	2.58E-05
**FAR1**	1.63245132	5.06E-12	6.69E-11
**OSBPL7**	1.63259904	1.85E-11	2.03E-10
**KCNMB1**	1.63459175	3.23E-11	3.28E-10
**SYK**	1.63467136	2.41E-11	2.53E-10
**TINAGL1**	1.63706083	4.47E-08	1.78E-07
**TAGLN**	1.63729685	5.56E-09	2.81E-08
**UNC5B**	1.6375255	1.03E-10	8.72E-10
**GXYLT2**	1.6382205	5.48E-09	2.78E-08
**CCIN**	1.63831289	9.46E-08	3.50E-07
**PYCR1**	1.63896714	1.69E-08	7.55E-08
**AMIGO2**	1.63904437	7.96E-09	3.86E-08
**ANXA2**	1.63953397	5.24E-17	7.08E-15
**SAMD13**	1.63954703	2.07E-09	1.18E-08
**TNFSF13**	1.63988343	2.29E-13	5.17E-12
**CAMKK1**	1.64060861	1.80E-11	1.98E-10
**HNF1B**	1.6407489	2.80E-05	6.16E-05
**ZNF287**	1.64082263	2.72E-10	2.01E-09
**NPHP1**	1.64191877	4.63E-12	6.24E-11
**KCNH1**	1.64247329	3.35E-04	6.10E-04
**GAL3ST4**	1.64274936	1.13E-12	1.95E-11
**IFT57**	1.64292075	9.78E-16	6.70E-14
**FCGR1A**	1.64367516	2.98E-12	4.37E-11
**GDPD3**	1.64387786	7.77E-11	6.91E-10
**KIAA1324**	1.64602789	7.59E-10	4.90E-09
**BRSK1**	1.64649015	1.45E-12	2.39E-11
**C5orf30**	1.6466873	2.56E-13	5.68E-12
**PAQR5**	1.64689085	5.62E-07	1.75E-06
**C20orf197**	1.6471001	2.16E-06	5.91E-06
**TRIP13**	1.64832902	5.00E-14	1.49E-12
**KCNE4**	1.64932773	6.03E-10	4.01E-09
**RPL39L**	1.65000477	1.85E-07	6.40E-07
**FGF1**	1.6507458	1.30E-07	4.68E-07
**MYH15**	1.65194507	2.45E-07	8.28E-07
**ATP10A**	1.65313385	3.04E-10	2.21E-09
**KCNK10**	1.65465389	6.11E-05	1.27E-04
**LAMA5**	1.65474532	1.30E-10	1.06E-09
**TRIM59**	1.65569667	1.71E-15	1.02E-13
**TMEM108**	1.65666889	1.71E-11	1.91E-10
**HTR2C**	1.65701814	7.00E-06	1.72E-05
**IMPDH1**	1.65745856	9.12E-16	6.33E-14
**VAV3**	1.65757261	3.72E-08	1.51E-07
**ADAM22**	1.65781199	2.83E-09	1.56E-08

**CRHR1**	1.65845497	2.93E-05	6.43E-05
**MICALL2**	1.65922151	2.22E-13	5.06E-12
**ZNF816**	1.65975861	3.16E-13	6.70E-12
**PDLIM7**	1.65978783	1.66E-13	3.96E-12
**MRGPRX3**	1.6599394	1.24E-11	1.44E-10
**SLC9A5**	1.65995901	1.09E-12	1.89E-11
**C8orf48**	1.66155608	2.86E-10	2.10E-09
**MYOZ1**	1.66189027	2.58E-08	1.09E-07
**ICAM5**	1.66189193	1.69E-08	7.53E-08
**P4HA3**	1.66335337	8.35E-10	5.32E-09
**LGALS9**	1.66411465	1.11E-12	1.92E-11
**TMSB4X**	1.66447692	9.45E-15	4.05E-13
**MAMSTR**	1.66454655	8.07E-12	9.93E-11
**PHLDA2**	1.66455406	2.83E-09	1.56E-08
**MAP1A**	1.6650073	4.35E-12	5.95E-11
**TSPAN2**	1.6654819	9.05E-08	3.36E-07
**ANKRD13B**	1.66760519	6.64E-12	8.45E-11
**MARK1**	1.66795746	1.92E-08	8.41E-08
**STAC**	1.66852284	3.46E-06	9.04E-06
**SYTL1**	1.6687652	2.76E-07	9.22E-07
**DTX3**	1.66903513	4.34E-10	3.02E-09
**IQUB**	1.66922121	1.58E-12	2.59E-11
**CCDC109B**	1.67006807	3.42E-13	7.16E-12
**ARL4C**	1.67096772	8.59E-11	7.46E-10
**IL2RG**	1.67194049	1.81E-09	1.05E-08
**ST14**	1.6732649	5.38E-06	1.35E-05
**MYADML2**	1.67425492	2.79E-04	5.16E-04
**GLIS2**	1.67638011	1.49E-12	2.45E-11
**RNF175**	1.67648437	7.51E-09	3.68E-08
**ACHE**	1.6774917	4.60E-07	1.46E-06
**MYEF2**	1.67751567	7.86E-09	3.82E-08
**B3GALNT1**	1.67757563	7.40E-17	9.02E-15
**CNN1**	1.67799309	2.02E-08	8.77E-08
**FHOD3**	1.67939056	2.11E-05	4.74E-05
**SLC35E4**	1.67942618	2.47E-17	3.90E-15
**SLC7A7**	1.68001076	1.86E-13	4.35E-12
**B3GALT4**	1.68015775	1.72E-10	1.35E-09
**PPAPDC3**	1.68104386	1.65E-10	1.30E-09
**KCNA5**	1.68166096	7.25E-05	1.49E-04
**MAP7D2**	1.68195933	2.37E-05	5.29E-05
**RNF186**	1.68317101	2.48E-04	4.64E-04
**ZNF880**	1.68322313	5.02E-09	2.57E-08
**TPM4**	1.68365967	6.09E-16	4.52E-14
**TRIM61**	1.68421404	2.11E-04	3.99E-04
**CD300LB**	1.68553871	1.24E-10	1.02E-09
**MARCKSL1**	1.68573113	2.80E-14	9.34E-13
**XKR3**	1.68694524	5.49E-04	9.62E-04
**ORAI2**	1.68742605	7.33E-15	3.29E-13
**SSC5D**	1.6882394	6.64E-07	2.03E-06
**DEFB124**	1.688798	1.31E-05	3.06E-05
**GLIS3**	1.68893793	8.14E-08	3.05E-07
**CPA4**	1.68936037	2.72E-05	6.00E-05
**CLSTN2**	1.68962161	1.20E-04	2.37E-04
**OR13A1**	1.68977763	2.93E-08	1.22E-07
**CMTM3**	1.6917937	9.78E-16	6.70E-14
**PADI2**	1.69223876	5.56E-09	2.81E-08
**SPRED3**	1.69315851	4.33E-09	2.26E-08
**TMC7**	1.69318126	1.14E-09	6.94E-09
**ANTXR1**	1.69399289	7.47E-09	3.65E-08
**C4orf48**	1.69415715	1.05E-09	6.47E-09
**HSPB2**	1.69439725	5.89E-12	7.59E-11
**DOK1**	1.69444688	8.51E-16	5.98E-14
**FAIM2**	1.69493556	2.07E-05	4.67E-05
**ISLR2**	1.69502105	6.19E-05	1.29E-04
**NSUN7**	1.69667637	1.43E-07	5.08E-07
**SOX4**	1.69708047	4.99E-12	6.63E-11
**THBS2**	1.69724113	4.26E-08	1.70E-07
**LCA5**	1.6973001	3.63E-09	1.94E-08
**KIAA1257**	1.69754103	6.74E-08	2.58E-07
**NEGR1**	1.69955952	1.19E-06	3.44E-06

**PPM1H**	1.70015385	9.75E-09	4.61E-08
**GSDME**	1.70166381	6.71E-14	1.90E-12
**NLRP4**	1.7024541	5.62E-06	1.41E-05
**TMC6**	1.70271495	1.59E-13	3.84E-12
**EYA2**	1.7049789	1.23E-06	3.54E-06
**TAF4B**	1.70508592	3.13E-07	1.03E-06
**CDC42EP5**	1.70508731	2.02E-11	2.19E-10
**HDAC9**	1.70566333	9.83E-08	3.63E-07
**TSPO**	1.70676913	4.19E-11	4.12E-10
**TRIM17**	1.70897849	1.33E-09	7.98E-09
**KCNQ1**	1.70992111	1.76E-10	1.38E-09
**DLGAP3**	1.7099883	0.0001288	0.00025273
**HES4**	1.71057081	2.82E-15	1.54E-13
**PLCH1**	1.71070975	2.84E-06	7.54E-06
**CCL2**	1.71148314	2.75E-06	7.34E-06
**ITGA2**	1.71262207	4.17E-10	2.91E-09
**SLC35F1**	1.71385369	1.19E-06	3.44E-06
**CYBA**	1.71406203	5.28E-11	4.98E-10
**MFGE8**	1.71480491	9.76E-14	2.57E-12
**MGP**	1.71482228	4.98E-08	1.97E-07
**PFKFB4**	1.71679711	7.67E-17	9.21E-15
**ADAM8**	1.71694358	1.80E-08	7.97E-08
**SLC24A5**	1.71716616	6.32E-09	3.16E-08
**ARMC9**	1.71865277	1.92E-16	1.90E-14
**HOXB3**	1.71906751	2.40E-08	1.02E-07
**COLEC12**	1.71934436	1.71E-08	7.63E-08
**CUBN**	1.72016979	5.26E-07	1.64E-06
**VENTX**	1.72103041	1.99E-09	1.14E-08
**NOS3**	1.72143628	2.66E-04	4.93E-04
**NCK2**	1.72233254	3.40E-15	1.78E-13
**GAS1**	1.72260587	3.54E-04	6.40E-04
**GPC2**	1.7229701	7.16E-14	2.00E-12
**RAB31**	1.72316905	3.90E-15	1.96E-13
**EMILIN2**	1.72390114	8.03E-14	2.18E-12
**NETO2**	1.72409194	6.01E-11	5.56E-10
**STK32B**	1.72443403	1.60E-11	1.80E-10
**EMP3**	1.72555226	1.09E-12	1.89E-11
**FAM132A**	1.72571964	2.75E-05	6.05E-05
**TAX1BP3**	1.72595307	8.44E-18	1.62E-15
**PRKCDBP**	1.7270119	4.27E-13	8.58E-12
**FAM171A2**	1.72732641	6.70E-09	3.32E-08
**KCNK13**	1.72777853	1.32E-10	1.08E-09
**FAM127C**	1.72787827	1.37E-10	1.11E-09
**EPS8L3**	1.72792901	2.28E-06	6.19E-06
**CCL11**	1.72838463	1.43E-07	5.08E-07
**NLRP12**	1.7284034	1.33E-09	7.98E-09
**LRRC49**	1.72886489	1.63E-09	9.59E-09
**C10orf55**	1.72998447	4.33E-13	8.68E-12
**IFI27L2**	1.73029628	1.49E-15	9.09E-14
**C1QTNF9B**	1.73121631	3.76E-04	6.78E-04
**KHDC1**	1.73163231	7.69E-09	3.75E-08
**LGALS3**	1.73194139	6.94E-11	6.29E-10
**PARM1**	1.73216092	1.65E-09	9.70E-09
**TFAP2C**	1.73219416	9.41E-07	2.78E-06
**CLMP**	1.73228336	3.27E-04	5.96E-04
**GPR97**	1.73250276	5.08E-05	1.07E-04
**ADAMTS14**	1.73254569	1.99E-11	2.16E-10
**MALL**	1.73258504	1.35E-07	4.83E-07
**GPRC5B**	1.73399877	1.22E-10	1.01E-09
**LEPREL1**	1.73463308	6.94E-06	1.71E-05
**LEPREL4**	1.73604134	5.46E-12	7.15E-11
**CACNB3**	1.73638147	6.16E-09	3.09E-08
**VIM**	1.7386948	1.08E-13	2.80E-12
**HCAR2**	1.74123458	1.67E-05	3.83E-05
**MAPK10**	1.74266678	9.15E-09	4.37E-08
**HLA-DQA2**	1.74480538	1.53E-04	0.00029698
**LSP1**	1.74522162	8.00E-11	7.05E-10
**AMPD3**	1.74525427	1.39E-14	5.45E-13
**MAGEL2**	1.74571142	1.15E-05	2.71E-05
**ZNF853**	1.74749944	3.18E-08	1.31E-07

TLL2	1.74829687	2.76E-10	2.04E-09
ABCC1	1.74863912	7.28E-14	2.02E-12
LOXL2	1.75071724	6.84E-13	1.27E-11
GULP1	1.75074329	3.14E-09	1.71E-08
CRMP1	1.75098025	4.03E-09	2.13E-08
ANO4	1.75211538	2.01E-10	1.55E-09
CACNB1	1.75267407	3.63E-11	3.62E-10
PHLDB1	1.75269	8.16E-14	2.21E-12
PPP2R3A	1.75437694	2.21E-17	3.63E-15
ZDHHC1	1.75457866	1.08E-09	6.63E-09
LY6G6C	1.75596216	6.52E-07	2.00E-06
OSBPL5	1.75671536	6.19E-10	4.11E-09
TTC39A	1.75808263	9.81E-13	1.73E-11
IGF1R	1.75821357	9.42E-10	5.91E-09
PTX3	1.75822528	9.35E-09	4.45E-08
HLA-DQB2	1.75880158	2.67E-05	5.89E-05
LIPM	1.7595807	7.02E-09	3.46E-08
HIST1H2BI	1.75964496	3.95E-06	1.02E-05
DAND5	1.75982558	7.26E-07	2.20E-06
FGF18	1.76116261	1.50E-05	3.47E-05
HIST3H2BB	1.76148214	2.85E-08	1.19E-07
G6PD	1.76197627	4.22E-12	5.82E-11
KAL1	1.76374709	7.86E-09	3.82E-08
PLN	1.76489598	5.70E-07	1.77E-06
CEP55	1.76501053	1.26E-14	5.05E-13
CHST5	1.76557919	3.05E-05	6.67E-05
GJC2	1.76885027	4.91E-11	4.72E-10
SLC12A2	1.76916938	2.22E-07	7.56E-07
GALNT6	1.76967871	2.09E-10	1.59E-09
DHDH	1.77242447	4.64E-11	4.49E-10
EFHC2	1.77293469	7.69E-08	2.90E-07
CD248	1.77404066	7.77E-14	2.14E-12
PLP2	1.77540253	3.58E-13	7.45E-12
TNFRSF13C	1.77580464	3.23E-06	8.52E-06
ID4	1.77627342	9.96E-07	2.92E-06
MFAP5	1.77737812	3.48E-07	1.13E-06
CPEB1	1.77883356	6.22E-07	1.91E-06
COL9A1	1.77951845	3.13E-05	6.83E-05
CYP26B1	1.77962855	3.67E-07	1.19E-06
NRARP	1.77998855	1.14E-11	1.34E-10
FABP3	1.78014418	6.28E-07	1.93E-06
MYLK2	1.78037476	2.88E-12	4.25E-11
XYLT1	1.78293833	3.59E-08	1.46E-07
KLHL35	1.78294657	1.13E-09	6.88E-09
ARHGEF25	1.78309354	3.59E-08	1.46E-07
ISYNA1	1.78354752	2.63E-11	2.74E-10
RAB19	1.78412031	2.13E-04	4.02E-04
DEPDC1B	1.78435374	7.66E-11	6.83E-10
IL1B	1.78524481	9.60E-08	3.55E-07
DPYSL4	1.78562466	1.65E-06	4.61E-06
KIF26B	1.78638671	2.58E-07	8.66E-07
CDKN1C	1.7890729	2.61E-10	1.94E-09
PLEKHN1	1.78943732	1.94E-08	8.51E-08
ALDH3B1	1.7902479	2.73E-13	5.97E-12
HOXA1	1.79062443	4.80E-07	1.52E-06
NEK11	1.79278804	2.44E-13	5.43E-12
CLEC4D	1.79331216	7.29E-06	1.79E-05
WDR38	1.79390467	7.74E-07	2.33E-06
SLC5A5	1.79530074	1.06E-06	3.09E-06
PRR15L	1.79722589	1.18E-04	2.33E-04
GPNMB	1.79872834	2.02E-08	8.77E-08
C1orf170	1.80001977	1.01E-08	4.74E-08
NXN	1.80336942	1.80E-08	7.97E-08
EMR3	1.80375246	4.39E-09	2.29E-08
WFDC1	1.80494105	2.10E-06	5.75E-06
STK31	1.80495402	4.91E-04	8.67E-04
PFN2	1.8067689	3.21E-12	4.66E-11
TMEM200A	1.80755666	5.76E-11	5.36E-10
TIMP2	1.80762833	3.52E-12	5.01E-11
ADM	1.80868646	8.46E-07	2.53E-06

**ZDHHC13**	1.80938419	2.50E-15	1.40E-13
**CCL20**	1.80947877	1.29E-06	3.70E-06
**SLC5A12**	1.80980572	2.96E-04	5.44E-04
**TCTN2**	1.81040925	9.45E-14	2.50E-12
**PCDHB7**	1.81153094	1.60E-05	3.69E-05
**PMP22**	1.81184271	1.39E-13	3.47E-12
**IL31RA**	1.81208477	4.13E-07	1.32E-06
**ELOVL7**	1.81298936	1.30E-07	4.68E-07
**FHL2**	1.81478085	1.16E-05	2.75E-05
**SAGE1**	1.8159662	6.68E-06	1.65E-05
**LGALS1**	1.81697435	3.69E-18	9.61E-16
**FGF11**	1.81737395	8.13E-13	1.47E-11
**AP1M2**	1.81984638	1.50E-08	6.77E-08
**C7orf34**	1.81996634	0.00014223	0.00027732
**DNAH7**	1.82322975	6.27E-06	1.56E-05
**CCL22**	1.82376029	6.04E-06	1.50E-05
**ENPP5**	1.82389532	1.26E-08	5.80E-08
**ICA1L**	1.82413858	4.22E-12	5.82E-11
**HS6ST3**	1.82457257	1.12E-04	2.22E-04
**KCNH8**	1.82497514	2.16E-04	4.08E-04
**TMEM54**	1.826891	1.82E-12	2.90E-11
**SBK1**	1.8270554	2.59E-09	1.44E-08
**WDR54**	1.8272705	1.14E-18	4.05E-16
**CCNI2**	1.82743348	1.05E-09	6.47E-09
**PRSS3**	1.82805199	6.73E-05	1.39E-04
**KCNMB4**	1.82861527	2.41E-11	2.53E-10
**P2RY1**	1.83002433	3.06E-04	5.62E-04
**GPX8**	1.83007849	2.83E-11	2.92E-10
**SERPINE2**	1.83117497	9.16E-08	3.40E-07
**CPXM1**	1.83151587	2.79E-10	2.06E-09
**YPEL4**	1.83190178	1.15E-13	2.96E-12
**SMARCD3**	1.83204685	5.05E-10	3.44E-09
**DDX43**	1.83262327	5.53E-04	9.69E-04
**C2orf27A**	1.83320159	5.68E-14	1.65E-12
**QPCT**	1.83331494	2.44E-13	5.43E-12
**HRASLS**	1.83366625	4.74E-06	1.21E-05
**MYBL2**	1.83387277	1.08E-13	2.80E-12
**HOXC4**	1.8343926	1.08E-09	6.64E-09
**ENTPD2**	1.83485995	1.17E-06	3.39E-06
**SYNDIG1**	1.83676844	6.82E-06	1.68E-05
**PCSK1N**	1.83880367	2.88E-05	6.33E-05
**GOLGA7B**	1.83958676	1.20E-07	4.35E-07
**EVC2**	1.84011476	8.26E-09	3.99E-08
**BAIAP2L2**	1.84078961	2.86E-07	9.51E-07
**CHST1**	1.84115901	3.44E-10	2.46E-09
**C11orf91**	1.84125231	2.64E-09	1.46E-08
**HOMER3**	1.84185404	1.34E-16	1.44E-14
**GPRC5D**	1.84274698	3.21E-12	4.66E-11
**FAM19A3**	1.84347136	2.53E-06	6.81E-06
**SLC25A36**	1.84371896	1.12E-10	9.37E-10
**PLAT**	1.84530318	1.46E-06	4.12E-06
**SIGLEC10**	1.84610932	5.49E-09	2.78E-08
**FABP5**	1.84642633	2.84E-12	4.22E-11
**TMEM119**	1.84767012	1.66E-08	7.44E-08
**CCL28**	1.84811275	4.83E-04	8.54E-04
**CARD11**	1.84930121	7.25E-07	2.20E-06
**GPR35**	1.84931579	1.01E-06	2.95E-06
**ZNF486**	1.85013445	4.84E-10	3.32E-09
**GTF2A1L**	1.8510858	3.06E-08	1.27E-07
**HES7**	1.85230816	1.37E-08	6.23E-08
**SDK2**	1.85296873	1.33E-09	7.99E-09
**HDGFRP3**	1.85548568	8.93E-10	5.64E-09
**C11orf63**	1.85660809	2.55E-08	1.08E-07
**KIAA1024**	1.85822289	3.23E-09	1.75E-08
**PTPRS**	1.85823157	9.55E-06	2.29E-05
**AFF2**	1.86041169	2.11E-05	4.75E-05
**ULBP1**	1.86056539	3.53E-09	1.89E-08
**GPR173**	1.86112092	1.22E-08	5.64E-08
**DPYSL3**	1.86132503	7.96E-09	3.86E-08
**WDR66**	1.86159658	2.55E-09	1.42E-08

**SNX32**	1.86170969	6.77E-10	4.45E-09
**TUSC3**	1.86210837	3.23E-11	3.28E-10
**UNC13D**	1.86248089	2.69E-09	1.49E-08
**TMTC2**	1.86278307	2.96E-11	3.04E-10
**HAS1**	1.8639291	2.12E-04	4.01E-04
**GAS7**	1.86398268	6.74E-13	1.26E-11
**GPR161**	1.86463153	5.00E-14	1.49E-12
**IL37**	1.86654457	1.13E-05	2.68E-05
**ITGA11**	1.86716821	2.87E-09	1.58E-08
**RBM20**	1.86824727	1.10E-04	0.00021853
**PRSS23**	1.86950229	9.13E-15	3.96E-13
**CRISPLD1**	1.86992349	1.54E-05	3.55E-05
**ANKRD18A**	1.87040362	1.76E-04	3.38E-04
**SLC7A1**	1.87065804	1.15E-07	4.17E-07
**FSCN1**	1.87126649	1.01E-13	2.65E-12
**LRRN4**	1.87328363	5.71E-04	9.98E-04
**SLFN13**	1.87385952	5.10E-08	2.01E-07
**MITF**	1.87403249	1.94E-11	2.11E-10
**RHPN1**	1.8760526	1.78E-08	7.88E-08
**NEFH**	1.87683735	5.49E-09	2.78E-08
**MFSD10**	1.87717948	1.52E-16	1.58E-14
**TREML1**	1.877244	6.04E-09	3.03E-08
**DBNDD2**	1.87792712	3.76E-18	9.61E-16
**ADAMDEC1**	1.87852433	3.77E-08	1.53E-07
**PLAU**	1.87864142	5.25E-14	1.55E-12
**MXRA8**	1.87922391	5.22E-09	2.66E-08
**TNFSF9**	1.88032025	1.32E-06	3.77E-06
**FERMT1**	1.8805744	8.66E-05	1.75E-04
**CR1L**	1.88060293	0.00019478	0.00037101
**PNMA2**	1.88115174	2.61E-07	8.75E-07
**C2CD4A**	1.88146555	4.33E-07	1.38E-06
**NPNT**	1.88166082	2.74E-08	1.15E-07
**TNFRSF6B**	1.8853171	1.08E-07	3.95E-07
**SNAP25**	1.88571026	1.86E-06	5.15E-06
**WDR49**	1.88584365	2.41E-06	6.52E-06
**MMP2**	1.88770045	4.53E-10	3.13E-09
**PTGES**	1.88784079	1.69E-08	7.53E-08
**TNFSF15**	1.88912221	1.71E-08	7.63E-08
**PAK6**	1.88918296	4.91E-14	1.47E-12
**LPPR3**	1.88994316	0.00016051	0.00031012
**CD44**	1.89029732	5.41E-10	3.64E-09
**SPATA17**	1.89108441	2.10E-08	9.10E-08
**LUZP4**	1.89164983	1.12E-04	2.23E-04
**GABBR1**	1.89211195	1.64E-06	4.57E-06
**DUSP26**	1.89246457	7.57E-07	2.29E-06
**LFNG**	1.89287985	2.37E-13	5.32E-12
**PRDM16**	1.89321386	5.75E-05	1.20E-04
**RARG**	1.89517758	4.91E-10	3.36E-09
**XKR6**	1.89550555	7.07E-11	6.40E-10
**AKR1B1**	1.89584129	3.28E-11	3.32E-10
**TEAD4**	1.89593979	1.92E-16	1.90E-14
**CACNA1F**	1.89702687	1.02E-06	2.98E-06
**OBSCN**	1.89716088	9.60E-08	3.55E-07
**KRT36**	1.89770487	5.43E-06	1.37E-05
**SULT1C4**	1.89940366	0.00025499	0.00047446
**LXN**	1.90099984	1.02E-09	6.34E-09
**FHDC1**	1.90336355	3.28E-11	3.32E-10
**C16orf93**	1.90347984	1.14E-14	4.64E-13
**KIF5C**	1.90441632	2.41E-07	8.13E-07
**FMN1**	1.90512739	1.79E-10	1.39E-09
**SOX15**	1.90576228	1.10E-05	2.60E-05
**JAG1**	1.9058506	7.64E-13	1.40E-11
**FIBIN**	1.90627692	4.29E-07	1.37E-06
**MAP9**	1.90676628	3.06E-08	1.27E-07
**PLA2G4A**	1.90773046	5.20E-07	1.63E-06
**HMGA2**	1.90897016	5.08E-11	4.85E-10
**HRASLS5**	1.9097318	5.34E-04	9.37E-04
**KCNS1**	1.91001575	7.24E-07	2.19E-06
**ELOVL4**	1.91098704	5.25E-09	2.68E-08
**N4BP3**	1.91182297	7.58E-15	3.37E-13

**SNPH**	1.91294588	3.80E-12	5.33E-11
**SEMA3A**	1.91331495	3.40E-09	1.83E-08
**STX1A**	1.91341119	1.30E-14	5.15E-13
**CLIP2**	1.91342482	1.38E-11	1.58E-10
**RGS4**	1.91451123	7.15E-06	1.76E-05
**BICC1**	1.91604238	2.37E-04	4.45E-04
**TNFRSF21**	1.91797279	1.01E-14	4.23E-13
**MFSD4**	1.91806226	2.38E-05	5.31E-05
**C15orf52**	1.91857552	3.47E-07	1.13E-06
**SMOX**	1.91872375	5.95E-17	7.91E-15
**SMPDL3B**	1.92043629	5.51E-11	5.15E-10
**KEL**	1.92224138	2.68E-04	4.97E-04
**CNFN**	1.92288185	2.96E-09	1.61E-08
**SYNDIG1L**	1.92362948	9.94E-06	2.38E-05
**TM4SF1**	1.92591686	2.80E-12	4.18E-11
**GNA15**	1.92859431	1.20E-13	3.08E-12
**WTIP**	1.92878008	5.22E-12	6.88E-11
**TMEM59L**	1.92878332	3.03E-09	1.65E-08
**DSG2**	1.92895683	2.06E-10	1.57E-09
**GLI3**	1.93009176	7.94E-08	2.99E-07
**CDC42BPG**	1.9309254	2.90E-06	7.69E-06
**ASPHD2**	1.93120035	9.68E-10	6.05E-09
**CSDC2**	1.93133425	2.28E-06	6.21E-06
**HAP1**	1.93161571	1.43E-06	4.06E-06
**NCS1**	1.93202914	9.61E-15	4.10E-13
**LOXL1**	1.93319045	4.16E-09	2.18E-08
**MN1**	1.93335702	1.82E-08	8.06E-08
**GCM1**	1.93400698	3.18E-04	5.81E-04
**HIST3H2A**	1.93631497	1.49E-10	1.20E-09
**SEL1L3**	1.93869964	3.30E-10	2.38E-09
**RBM11**	1.93873616	8.50E-07	2.54E-06
**GLI2**	1.94195288	6.77E-07	2.06E-06
**PKIA**	1.94291438	4.47E-13	8.91E-12
**VSIG8**	1.94296756	1.85E-09	1.07E-08
**ZNF215**	1.9436058	1.24E-10	1.02E-09
**FCGBP**	1.94387331	1.06E-09	6.54E-09
**SLC6A6**	1.94475219	5.98E-15	2.82E-13
**GREM1**	1.9468091	4.30E-08	1.72E-07
**NACAD**	1.94816137	8.50E-10	5.41E-09
**ELF4**	1.9482051	5.78E-15	2.75E-13
**NRSN1**	1.94861604	3.41E-04	6.19E-04
**MTHFD2**	1.94888385	5.00E-14	1.49E-12
**OSCAR**	1.94915296	3.47E-13	7.25E-12
**PLEKHH2**	1.94988428	1.67E-08	7.47E-08
**LPCAT4**	1.95249379	1.92E-16	1.90E-14
**PTGDS**	1.95404756	9.75E-06	2.34E-05
**LILRB4**	1.95466084	6.45E-10	4.25E-09
**SFXN3**	1.95596759	7.01E-17	8.81E-15
**FNDC1**	1.95607575	2.11E-08	9.11E-08
**ANKRD7**	1.95704363	1.05E-04	0.00020988
**CASC1**	1.9582869	2.24E-07	7.60E-07
**LSAMP**	1.96089994	2.74E-06	7.32E-06
**PRICKLE1**	1.96134178	5.17E-06	1.30E-05
**C10orf91**	1.96406965	8.91E-10	5.64E-09
**FAM27B**	1.96467864	1.09E-11	1.29E-10
**LOX**	1.96482442	3.20E-14	1.05E-12
**ZNF185**	1.96491567	2.23E-12	3.43E-11
**FAM57A**	1.96569648	9.96E-13	1.75E-11
**ADAM32**	1.96572624	7.98E-14	2.17E-12
**IL2RA**	1.96975688	5.12E-10	3.48E-09
**PCNXL2**	1.97136018	1.05E-09	6.47E-09
**ZNF695**	1.97232636	1.88E-08	8.26E-08
**AANAT**	1.97295933	1.03E-09	6.38E-09
**HAPLN3**	1.97355882	6.74E-12	8.53E-11
**RLN2**	1.97468343	6.68E-08	2.56E-07
**NHS**	1.975028	1.32E-07	4.73E-07
**DZIP1**	1.97691892	1.27E-07	4.58E-07
**LAYN**	1.97977968	4.17E-14	1.30E-12
**LPAR1**	1.9803684	1.47E-07	5.22E-07
**FLNA**	1.98096509	1.67E-14	6.21E-13

**PAPLN**	1.98328637	4.64E-09	2.40E-08
**FAP**	1.9836497	1.35E-10	1.10E-09
**TMED3**	1.98405312	1.25E-15	7.97E-14
**TBX1**	1.98686678	3.03E-07	1.00E-06
**CHI3L2**	1.9869954	1.57E-06	4.41E-06
**CDCA7**	1.99001518	2.15E-09	1.22E-08
**ARMCX2**	1.99025697	1.17E-10	9.74E-10
**CT45A5**	1.99050074	3.73E-06	9.69E-06
**SLITRK4**	1.99051457	6.13E-11	5.66E-10
**CHRNA5**	1.99094524	1.54E-06	4.32E-06
**ACTG2**	1.99197468	3.93E-07	1.26E-06
**BCL11A**	1.9923239	3.80E-07	1.23E-06
**DNAJB13**	1.99347319	5.42E-05	1.14E-04
**BEND6**	1.99422265	1.77E-08	7.84E-08
**FZD1**	1.99574339	6.93E-14	1.95E-12
**CEBPE**	1.99574729	9.41E-11	8.07E-10
**MORN3**	1.99648542	2.19E-10	1.66E-09
**PELI2**	1.99761892	3.59E-07	1.17E-06
**CYTL1**	1.99845575	5.78E-09	2.91E-08
**HOXC9**	1.99976024	7.43E-08	2.82E-07
**ADH7**	2.00283528	7.52E-05	1.54E-04
**GRID1**	2.00400241	2.68E-07	8.98E-07
**PBX4**	2.00406458	2.72E-10	2.01E-09
**FBLN1**	2.00429347	5.72E-05	1.20E-04
**DLG3**	2.00436396	3.63E-11	3.62E-10
**PTK7**	2.0055247	1.28E-09	7.72E-09
**BRSK2**	2.0071116	3.53E-06	9.22E-06
**TANC2**	2.00934325	7.39E-10	4.78E-09
**FAM129B**	2.01006338	1.80E-15	1.06E-13
**REG1A**	2.0102785	2.14E-04	4.05E-04
**ALDOA**	2.01065315	1.90E-18	6.00E-16
**LARP6**	2.01109148	3.30E-10	2.38E-09
**ALOX15B**	2.01324196	1.48E-06	4.19E-06
**FBLIM1**	2.01401597	1.22E-17	2.24E-15
**KCNH2**	2.01466231	1.48E-08	6.70E-08
**SNCA**	2.01626025	3.65E-11	3.64E-10
**SLITRK5**	2.01664293	1.65E-06	4.62E-06
**CCL18**	2.01683206	4.64E-04	8.23E-04
**SH3D21**	2.01743993	1.71E-17	2.89E-15
**C7orf61**	2.01815302	5.48E-10	3.69E-09
**WISP1**	2.01875245	1.18E-08	5.45E-08
**PLSCR2**	2.0202327	1.55E-04	3.00E-04
**NKPD1**	2.02187738	3.43E-08	1.41E-07
**FAM160A1**	2.02284866	6.75E-07	2.06E-06
**NRIP3**	2.02309801	1.13E-13	2.93E-12
**CPZ**	2.02350604	2.18E-07	7.43E-07
**EGR4**	2.02473539	1.10E-06	3.19E-06
**ZNF280B**	2.02557035	1.30E-07	4.67E-07
**MRC2**	2.02704383	5.21E-11	4.93E-10
**KCNF1**	2.02833515	6.95E-09	3.43E-08
**CLEC4E**	2.03039962	4.77E-08	1.89E-07
**TRIM46**	2.03083651	5.80E-12	7.51E-11
**DNM1**	2.03089892	4.02E-07	1.29E-06
**ZP1**	2.0330289	1.49E-06	4.21E-06
**KCNH3**	2.03365365	4.10E-08	1.65E-07
**VCAN**	2.03457177	6.27E-11	5.75E-10
**CD24**	2.035797	5.43E-11	5.09E-10
**FXYD5**	2.03584208	1.15E-12	1.97E-11
**CDH26**	2.03756398	5.47E-05	1.15E-04
**TREM2**	2.03855869	1.77E-13	4.19E-12
**WFDC3**	2.03990531	7.71E-10	4.97E-09
**RAB34**	2.039996	6.74E-12	8.53E-11
**GALNT7**	2.04010754	2.61E-10	1.94E-09
**CCDC8**	2.04023957	5.81E-09	2.93E-08
**SSPN**	2.04082156	8.47E-11	7.38E-10
**IL18**	2.04099718	1.34E-12	2.24E-11
**BEAN1**	2.04112022	2.80E-05	6.16E-05
**TUBB6**	2.04128506	4.70E-12	6.31E-11
**SDCBP2**	2.04235607	8.14E-08	3.05E-07
**MEIS3**	2.04281062	3.92E-12	5.44E-11

**ST6GALNAC5**	2.04442353	1.77E-11	1.95E-10
**IL17RD**	2.04518416	3.84E-10	2.71E-09
**MPP2**	2.04588532	4.44E-12	6.04E-11
**MYO10**	2.04806239	1.13E-04	2.24E-04
**WNT2**	2.05106074	4.76E-07	1.51E-06
**METRNL**	2.05140005	3.78E-14	1.20E-12
**KIAA1644**	2.05268641	1.71E-06	4.77E-06
**BEGAIN**	2.05273353	1.89E-04	3.60E-04
**FAM81A**	2.0593276	1.34E-12	2.24E-11
**COL6A3**	2.06057051	1.17E-09	7.11E-09
**PMAIP1**	2.06159106	4.11E-10	2.88E-09
**KIF3C**	2.06539253	3.05E-14	1.00E-12
**B3GNT8**	2.0676367	5.12E-10	3.48E-09
**COL3A1**	2.07221733	2.87E-09	1.58E-08
**NXPH2**	2.07431635	1.18E-04	2.33E-04
**TMEM61**	2.07447514	1.83E-05	4.16E-05
**MCOLN3**	2.07631849	2.89E-09	1.59E-08
**LDOC1**	2.07850065	2.09E-06	5.72E-06
**TYRO3**	2.08025151	1.03E-12	1.80E-11
**PDZRN3**	2.0809597	8.54E-07	2.55E-06
**MAPK13**	2.08300026	1.59E-15	9.60E-14
**SRRM3**	2.0845994	2.98E-05	6.52E-05
**OLFML2B**	2.08774143	4.60E-16	3.50E-14
**CCNJL**	2.08900685	4.62E-13	9.10E-12
**TMEM190**	2.0892034	3.80E-09	2.02E-08
**B3GALT2**	2.09022782	2.45E-05	5.45E-05
**SPEF1**	2.09219115	6.45E-08	2.48E-07
**PAQR8**	2.09660426	2.99E-10	2.19E-09
**FGR**	2.09726003	8.06E-09	3.90E-08
**IDO1**	2.09767396	1.05E-04	0.00021016
**NOV**	2.10123691	1.71E-08	7.63E-08
**TMSB10**	2.10226018	2.58E-18	7.68E-16
**MMP14**	2.10483465	4.06E-18	9.89E-16
**NCEH1**	2.10855418	1.76E-14	6.43E-13
**MCTP2**	2.10899842	5.75E-08	2.24E-07
**KRT7**	2.10921621	1.77E-05	4.04E-05
**SULF1**	2.10930314	4.84E-12	6.48E-11
**CADPS**	2.11029341	7.74E-06	1.89E-05
**RRAD**	2.11255649	2.11E-05	4.74E-05
**COL5A1**	2.11320913	1.59E-09	9.37E-09
**ARNT2**	2.11659629	1.51E-08	6.82E-08
**HUNK**	2.11680613	2.38E-05	5.31E-05
**RIN1**	2.1197059	1.25E-15	7.97E-14
**BAI1**	2.1197121	5.14E-07	1.61E-06
**FZD2**	2.12166941	3.07E-12	4.48E-11
**IL8**	2.12244713	4.72E-10	3.24E-09
**TMEM51**	2.1224591	2.65E-16	2.28E-14
**ARHGDIG**	2.12599602	9.93E-06	2.38E-05
**KCNG1**	2.12834039	2.10E-08	9.08E-08
**KCNK2**	2.12915758	1.23E-06	3.53E-06
**EFCAB4A**	2.13111506	6.08E-09	3.05E-08
**KIAA1377**	2.13340233	1.62E-14	6.08E-13
**C1orf186**	2.13429714	4.89E-06	1.24E-05
**LRP8**	2.1371883	2.52E-11	2.63E-10
**UBASH3B**	2.13769544	3.65E-14	1.17E-12
**ABR**	2.13775975	5.67E-13	1.09E-11
**PF4**	2.13919896	4.98E-04	8.79E-04
**AMPH**	2.13935782	1.09E-05	2.58E-05
**TPPP3**	2.13998195	1.54E-12	2.51E-11
**TGFB2**	2.14022203	3.84E-10	2.71E-09
**SEMA6A**	2.14348977	2.33E-09	1.31E-08
**CCDC78**	2.14538338	4.99E-11	4.77E-10
**C1orf65**	2.14618876	9.77E-05	1.96E-04
**NKX3-2**	2.14627113	2.40E-07	8.12E-07
**PTPRN2**	2.14782972	8.31E-06	2.02E-05
**NDRG4**	2.1481621	3.49E-09	1.87E-08
**HAS2**	2.15211	1.26E-05	2.96E-05
**C2orf81**	2.15261553	1.92E-16	1.90E-14
**GRID2IP**	2.16144801	3.23E-09	1.75E-08
**USH1C**	2.16569176	1.31E-07	4.69E-07

**ILDR1**	2.1663379	5.60E-10	3.76E-09
**GUCA2A**	2.16636755	3.22E-04	5.89E-04
**KCNQ3**	2.16649604	3.26E-12	4.71E-11
**TRNP1**	2.16704398	5.36E-11	5.04E-10
**HOXC6**	2.1685188	5.88E-08	2.28E-07
**ZNF560**	2.16980412	2.65E-06	7.11E-06
**CAPN13**	2.17228553	8.10E-05	1.65E-04
**DBN1**	2.17316171	1.61E-16	1.66E-14
**OSM**	2.17361353	3.76E-13	7.75E-12
**EPHB6**	2.17583255	3.68E-09	1.96E-08
**RGS17**	2.17785501	2.91E-10	2.13E-09
**C7orf57**	2.17819639	2.37E-05	5.28E-05
**PCDP1**	2.18322243	2.49E-04	4.64E-04
**BCAS1**	2.18574957	2.69E-05	5.92E-05
**SAMD12**	2.18645113	1.79E-10	1.39E-09
**FZD10**	2.1881093	3.13E-08	1.30E-07
**FOSL1**	2.18828018	9.86E-07	2.89E-06
**NXPH4**	2.18861592	1.66E-12	2.68E-11
**JSRP1**	2.18929534	1.22E-04	2.41E-04
**IGSF9B**	2.19005004	5.15E-04	9.06E-04
**CHRNA6**	2.19104813	4.36E-08	1.74E-07
**TLR10**	2.19864417	3.05E-05	6.67E-05
**FAM64A**	2.20078926	6.08E-14	1.75E-12
**CNKSR1**	2.20263814	5.90E-06	1.47E-05
**CD207**	2.20327745	3.89E-04	7.01E-04
**HIST1H1D**	2.2043498	1.80E-09	1.05E-08
**FKBP10**	2.20520547	2.73E-13	5.97E-12
**RAP1GAP2**	2.20781839	4.29E-12	5.89E-11
**TMEM151A**	2.20883321	5.35E-07	1.67E-06
**FUT4**	2.20900025	5.80E-12	7.51E-11
**SLMO1**	2.21008486	1.16E-15	7.56E-14
**SLC30A8**	2.21013168	1.36E-04	2.67E-04
**FLRT2**	2.21013556	8.74E-05	1.77E-04
**GPC4**	2.2146474	1.58E-10	1.25E-09
**SPAG17**	2.21695501	1.65E-07	5.77E-07
**LGALS12**	2.22014033	3.13E-07	1.03E-06
**LHB**	2.22018038	2.84E-09	1.56E-08
**VANGL2**	2.22236864	3.81E-08	1.55E-07
**RTDR1**	2.22439343	3.29E-08	1.35E-07
**PCDHB6**	2.22575856	5.56E-05	1.16E-04
**SLC1A5**	2.22740366	6.28E-17	8.09E-15
**MLLT3**	2.22750251	3.71E-14	1.19E-12
**KIF5A**	2.22953967	6.91E-10	4.52E-09
**MXRA5**	2.2303797	2.95E-08	1.23E-07
**SPECC1**	2.23187426	5.76E-11	5.36E-10
**LINGO1**	2.23295011	2.79E-11	2.88E-10
**LRRC36**	2.23369547	7.68E-10	4.95E-09
**NBPF4**	2.24312436	0.00056774	0.00099257
**SLITRK1**	2.24325678	2.43E-04	4.54E-04
**TNFRSF11A**	2.24343416	1.94E-14	6.98E-13
**ADORA2B**	2.24385768	8.23E-11	7.20E-10
**DUSP4**	2.2442449	6.91E-10	4.51E-09
**S100A14**	2.24500882	2.03E-06	5.57E-06
**PRSS12**	2.24603209	1.18E-04	2.33E-04
**4-Mar**	2.24615632	2.60E-05	5.74E-05
**POU5F1**	2.25076898	1.03E-05	2.46E-05
**TMEM200C**	2.25192614	6.58E-07	2.01E-06
**STMN3**	2.25207939	6.63E-10	4.36E-09
**LRFN4**	2.25213697	1.26E-11	1.46E-10
**AATK**	2.2535376	2.21E-11	2.35E-10
**PDGFD**	2.25794317	4.39E-04	7.81E-04
**FOXE1**	2.25874666	4.51E-05	9.57E-05
**TDRD1**	2.26003233	1.78E-04	3.41E-04
**AIRE**	2.26069865	1.47E-05	3.39E-05
**COL13A1**	2.26124643	2.00E-10	1.54E-09
**CDH11**	2.26260976	2.18E-11	2.32E-10
**GALNT3**	2.26546991	2.24E-04	4.22E-04
**FOLR3**	2.26624108	7.65E-06	1.87E-05
**PRSS8**	2.26670681	1.53E-05	3.53E-05
**NANOS3**	2.2679768	2.43E-10	1.83E-09

**ULBP2**	2.27162584	5.08E-07	1.60E-06
**TSPAN15**	2.2734771	6.97E-15	3.17E-13
**CRLF1**	2.27674543	5.13E-10	3.48E-09
**CTBP2**	2.27873229	7.14E-11	6.45E-10
**SFN**	2.27893607	8.84E-08	3.29E-07
**MARVELD1**	2.28074949	1.32E-14	5.23E-13
**ANXA1**	2.28075051	2.06E-10	1.57E-09
**CRIP1**	2.28184084	6.13E-13	1.16E-11
**RNF212**	2.28198998	0.00020332	0.00038601
**C1orf106**	2.28309197	8.84E-11	7.64E-10
**ACPP**	2.28338241	8.20E-11	7.20E-10
**NALCN**	2.28866551	4.47E-07	1.42E-06
**ADAM12**	2.28920153	4.84E-12	6.48E-11
**PIWIL4**	2.29023468	5.12E-09	2.62E-08
**MYOF**	2.29193647	3.26E-12	4.71E-11
**B3GNT4**	2.292376	4.71E-10	3.24E-09
**NTRK2**	2.29488239	9.46E-05	1.90E-04
**JPH2**	2.29546604	1.25E-10	1.03E-09
**SLC38A5**	2.29553158	4.47E-09	2.32E-08
**CTF1**	2.29608577	5.48E-08	2.15E-07
**MBOAT2**	2.29646853	8.16E-09	3.94E-08
**NFE2**	2.29753997	5.56E-05	1.17E-04
**MMP1**	2.29799013	6.18E-10	4.11E-09
**HAGHL**	2.29823316	3.47E-12	4.94E-11
**PLEKHG4**	2.29851656	3.58E-10	2.56E-09
**GFPT2**	2.2988899	6.83E-09	3.38E-08
**SV2A**	2.30111796	1.51E-10	1.21E-09
**GCNT3**	2.30402477	5.19E-07	1.63E-06
**KIAA1211**	2.3043446	1.84E-06	5.10E-06
**STK32C**	2.30708723	5.80E-12	7.51E-11
**NME5**	2.30750164	1.17E-04	2.31E-04
**DNAH3**	2.3088771	3.91E-08	1.58E-07
**SLC22A16**	2.31149698	4.44E-11	4.33E-10
**ISLR**	2.31612287	7.46E-09	3.65E-08
**APCDD1**	2.3170413	6.10E-11	5.63E-10
**BCL2A1**	2.31978621	1.74E-10	1.36E-09
**DYX1C1**	2.32062421	4.42E-12	6.02E-11
**MMP11**	2.32864966	1.99E-11	2.16E-10
**TGM1**	2.32887812	9.50E-09	4.51E-08
**GRIP1**	2.33012378	3.15E-09	1.71E-08
**OLR1**	2.33653376	6.79E-12	8.58E-11
**RASGEF1A**	2.33915312	2.79E-10	2.06E-09
**PTHLH**	2.3397734	1.51E-10	1.21E-09
**CAPG**	2.34124918	1.25E-16	1.36E-14
**MYOM3**	2.34267097	2.19E-08	9.41E-08
**ZNF321P**	2.34794724	2.68E-15	1.47E-13
**CADM3**	2.34842571	7.72E-05	1.57E-04
**NKX2-8**	2.34955116	2.77E-10	2.05E-09
**ARL9**	2.35058227	1.64E-08	7.34E-08
**DUOX1**	2.35115947	4.97E-07	1.57E-06
**C1QL4**	2.35866163	2.69E-05	5.94E-05
**GAL3ST1**	2.35918745	2.07E-08	8.97E-08
**HOXC8**	2.36077105	1.33E-08	6.06E-08
**TMEM35**	2.36722567	1.29E-08	5.91E-08
**SPHK1**	2.36878012	1.62E-14	6.08E-13
**SLC6A8**	2.37074145	3.07E-09	1.67E-08
**CCDC114**	2.37148356	1.26E-06	3.61E-06
**CHST3**	2.37287548	1.51E-10	1.21E-09
**ADAM28**	2.37557882	1.94E-11	2.11E-10
**XIRP1**	2.37919009	5.10E-09	2.61E-08
**SCG2**	2.37964493	6.90E-13	1.28E-11
**FGFR1**	2.37968687	8.57E-12	1.04E-10
**TEKT2**	2.37974208	1.44E-09	8.61E-09
**C2CD4C**	2.38079708	1.08E-05	2.56E-05
**PFKFB3**	2.38260259	2.56E-12	3.87E-11
**RNF150**	2.38283186	6.11E-07	1.88E-06
**IBSP**	2.38341575	1.48E-08	6.69E-08
**CTNND2**	2.38593118	5.16E-08	2.03E-07
**ATG9B**	2.3860102	3.50E-08	1.43E-07
**TMPRSS13**	2.38601911	1.66E-07	5.80E-07

**ADAP1**	2.38890563	4.44E-11	4.33E-10
**LPAR2**	2.39006296	4.75E-14	1.45E-12
**B3GNT3**	2.39287879	1.96E-08	8.57E-08
**FXYD6-FXYD2**	2.39533089	6.59E-06	1.63E-05
**TMSB15B**	2.39627317	8.45E-12	1.03E-10
**PLAC1**	2.39673131	6.84E-05	1.41E-04
**ARHGAP40**	2.39937705	2.51E-05	5.55E-05
**DZIP1L**	2.3998974	1.44E-13	3.56E-12
**SHISA2**	2.40527522	8.94E-13	1.60E-11
**NOG**	2.40567115	2.34E-06	6.35E-06
**HOXB7**	2.40829419	2.09E-08	9.04E-08
**PLBD1**	2.40980752	6.60E-14	1.88E-12
**EHF**	2.41019434	2.25E-08	9.66E-08
**IGFBP6**	2.41184034	1.57E-09	9.27E-09
**TGFA**	2.41267208	5.38E-12	7.06E-11
**GPR84**	2.41388528	5.72E-13	1.10E-11
**CST2**	2.41797447	1.23E-07	4.46E-07
**TFAP2A**	2.4195963	8.66E-05	1.75E-04
**PRRX1**	2.42228093	9.96E-13	1.75E-11
**AQP1**	2.42487944	5.49E-06	1.38E-05
**HTRA4**	2.42564446	6.23E-09	3.12E-08
**CTHRC1**	2.42583105	1.46E-15	9.04E-14
**C16orf74**	2.42697141	7.09E-10	4.62E-09
**SCRN1**	2.42698844	3.88E-13	7.97E-12
**ALOX5AP**	2.42883375	7.40E-14	2.05E-12
**ZNF860**	2.42965988	1.15E-06	3.33E-06
**ASPHD1**	2.43310947	7.45E-07	2.25E-06
**POSTN**	2.43331021	2.33E-13	5.24E-12
**EPHX4**	2.43549497	2.93E-13	6.32E-12
**FAM155A**	2.44073468	5.85E-12	7.57E-11
**PODNL1**	2.44274486	8.59E-11	7.46E-10
**MMP9**	2.44442859	3.91E-08	1.58E-07
**BAI2**	2.44571531	1.99E-10	1.53E-09
**IL11**	2.44628964	1.28E-09	7.69E-09
**ISM2**	2.44894101	1.49E-04	2.89E-04
**HOXC5**	2.449915	4.14E-05	8.84E-05
**TPSG1**	2.45122217	7.84E-07	2.36E-06
**SYT5**	2.45425311	5.82E-06	1.45E-05
**HMSD**	2.45677525	3.28E-10	2.37E-09
**GPR56**	2.45693527	7.16E-12	8.94E-11
**PCDH7**	2.45899498	1.87E-06	5.18E-06
**VGLL3**	2.46179089	6.64E-08	2.54E-07
**SLC9A2**	2.46394564	2.06E-05	4.65E-05
**QSOX1**	2.46434646	2.79E-11	2.88E-10
**SV2B**	2.46643279	8.19E-06	1.99E-05
**NTM**	2.46845152	1.35E-11	1.55E-10
**SPAG6**	2.47359156	2.96E-04	5.44E-04
**LETM2**	2.47544663	2.90E-14	9.63E-13
**ATP6V0D2**	2.47570907	2.92E-10	2.14E-09
**GRIN2D**	2.47572697	3.21E-13	6.79E-12
**RAB27B**	2.47794098	3.36E-08	1.38E-07
**CLDN4**	2.48129755	3.68E-08	1.50E-07
**RGS20**	2.48239449	7.84E-07	2.36E-06
**KCND2**	2.48464488	1.41E-09	8.43E-09
**HOXB6**	2.48690642	2.72E-09	1.51E-08
**PRSS35**	2.4873548	4.31E-08	1.72E-07
**DPF1**	2.49152054	1.07E-13	2.80E-12
**PTPN13**	2.49605256	1.03E-08	4.82E-08
**COL12A1**	2.49975208	5.12E-10	3.48E-09
**PCLO**	2.49984594	8.82E-13	1.59E-11
**TPRXL**	2.50108253	3.67E-08	1.49E-07
**COL1A1**	2.50215231	7.77E-11	6.91E-10
**CERCAM**	2.50332672	3.58E-15	1.84E-13
**KLHL30**	2.50360803	1.30E-09	7.81E-09
**DDR1**	2.50895682	5.22E-09	2.66E-08
**BRDT**	2.50960107	1.74E-04	3.34E-04
**PNCK**	2.51076815	5.95E-11	5.53E-10
**CRYAB**	2.51092588	1.18E-09	7.19E-09
**COL1A2**	2.51490553	3.68E-11	3.66E-10
**WDR63**	2.52779595	1.84E-12	2.92E-11

**OXTR**	2.52782902	1.77E-11	1.95E-10
**SCUBE3**	2.53313786	7.36E-09	3.61E-08
**FAM19A4**	2.54083434	6.77E-07	2.06E-06
**AQP10**	2.541114	4.75E-05	0.00010059
**HPDL**	2.54249581	0.00046392	0.00082293
**LAMB3**	2.54317094	2.37E-06	6.43E-06
**DMBT1**	2.54527833	3.64E-10	2.59E-09
**APLP1**	2.5460045	8.43E-08	3.15E-07
**TMPRSS3**	2.54822042	1.64E-06	4.57E-06
**SCRG1**	2.55041138	0.00030495	0.00055973
**KIRREL2**	2.56142051	5.74E-08	2.24E-07
**SCD5**	2.56269151	1.71E-08	7.63E-08
**C11orf70**	2.56405702	7.49E-13	1.38E-11
**HOPX**	2.56656999	3.86E-12	5.38E-11
**FAM27A**	2.56793763	7.69E-13	1.41E-11
**PLA2G4F**	2.5679858	1.69E-06	4.71E-06
**POF1B**	2.56805635	1.82E-09	1.06E-08
**TMEM158**	2.56934815	1.66E-16	1.71E-14
**BHLHE41**	2.57244762	4.63E-12	6.24E-11
**CLCNKB**	2.57257031	4.15E-09	2.18E-08
**HOXC10**	2.57301693	1.65E-05	3.78E-05
**LIF**	2.57326395	3.94E-13	8.07E-12
**OR2H2**	2.57412237	2.62E-06	7.03E-06
**FXYD2**	2.57552462	2.52E-07	8.48E-07
**SUN3**	2.57663673	5.93E-06	1.48E-05
**ARSI**	2.5790798	3.68E-06	9.58E-06
**LY6H**	2.58918121	2.48E-09	1.39E-08
**DRP2**	2.58965662	1.44E-13	3.56E-12
**TM4SF19**	2.59175419	4.47E-13	8.91E-12
**FRAS1**	2.59416336	1.79E-07	6.21E-07
**HOXB5**	2.59983417	1.79E-10	1.39E-09
**TNNI2**	2.60882307	3.90E-11	3.86E-10
**LEFTY1**	2.60883844	1.83E-07	6.34E-07
**PRB3**	2.60925457	2.05E-06	5.64E-06
**PLEKHG4B**	2.61043464	0.00010524	0.00021016
**ITPR3**	2.61154004	4.47E-13	8.91E-12
**KRTAP1-1**	2.6119021	2.54E-05	5.63E-05
**BMP7**	2.61209989	3.88E-06	1.00E-05
**FGF9**	2.61264299	5.75E-09	2.90E-08
**CXCL6**	2.61334354	3.01E-08	1.25E-07
**KIAA0319**	2.61384103	6.42E-07	1.97E-06
**FBXL2**	2.61728271	1.28E-14	5.11E-13
**SLC16A5**	2.61830867	2.03E-10	1.55E-09
**HAPLN1**	2.61929237	1.81E-06	5.02E-06
**PTPLA**	2.62114688	9.20E-09	4.39E-08
**HEPH**	2.62281183	5.41E-10	3.64E-09
**C12orf75**	2.62288953	3.26E-13	6.88E-12
**DIRAS1**	2.62658843	1.74E-10	1.36E-09
**CHRNA7**	2.62759976	3.68E-10	2.62E-09
**CDH6**	2.63109919	2.34E-11	2.47E-10
**CACNG8**	2.63333484	3.58E-08	1.46E-07
**B3GNT7**	2.63575819	2.48E-11	2.60E-10
**RASSF6**	2.63726549	3.73E-10	2.65E-09
**HK2**	2.64114276	3.78E-14	1.20E-12
**C15orf48**	2.64761333	6.64E-12	8.45E-11
**PRSS16**	2.65354152	2.06E-07	7.06E-07
**COL8A2**	2.65442592	4.62E-12	6.24E-11
**BACE2**	2.65661927	1.85E-11	2.03E-10
**RPRM**	2.65682841	2.94E-06	7.81E-06
**CCL13**	2.66025303	1.04E-06	3.03E-06
**PLAUR**	2.66157053	3.34E-16	2.70E-14
**PITX1**	2.66264018	4.73E-08	1.88E-07
**GAP43**	2.66617167	1.82E-12	2.90E-11
**KLF5**	2.66945091	1.05E-09	6.47E-09
**C5orf46**	2.67045172	2.96E-12	4.35E-11
**TMEM159**	2.67219148	4.71E-11	4.54E-10
**EGLN3**	2.67504367	1.45E-12	2.39E-11
**TMSB15A**	2.68125073	6.26E-08	2.41E-07
**BCL2L15**	2.6831575	0.00029866	0.000549
**FABP6**	2.68614034	2.63E-08	1.11E-07

**FUT2**	2.68838403	7.28E-09	3.57E-08
**RHBDL2**	2.68843592	1.16E-12	1.99E-11
**VGLL2**	2.68975408	7.91E-05	0.00016105
**ELOVL3**	2.69043039	2.12E-06	5.81E-06
**HOXC11**	2.69364714	5.04E-05	0.00010631
**ALOX5**	2.69855724	6.33E-13	1.19E-11
**HTRA3**	2.70323351	1.40E-11	1.60E-10
**GRM5**	2.7051252	1.00E-06	2.93E-06
**C20orf195**	2.70699636	8.59E-07	2.56E-06
**STK33**	2.71658578	4.91E-08	1.94E-07
**TNC**	2.71705711	2.15E-09	1.22E-08
**C6orf141**	2.71762331	3.49E-05	7.55E-05
**CDKL2**	2.71920302	4.29E-05	9.14E-05
**RIMKLA**	2.7244172	8.84E-07	2.62E-06
**PPFIA4**	2.72746023	4.00E-13	8.17E-12
**UCHL1**	2.72985089	1.51E-14	5.81E-13
**USH1G**	2.73036446	0.00023587	0.00044268
**CCDC19**	2.7375654	5.43E-11	5.09E-10
**ZFP57**	2.73864373	3.91E-06	1.01E-05
**PITPNM3**	2.740448	0.00017276	0.00033211
**CLIC6**	2.74338391	9.65E-08	3.56E-07
**EPCAM**	2.74339178	8.00E-11	7.05E-10
**EFNA5**	2.74426256	4.04E-09	2.13E-08
**PLXNB3**	2.75081761	2.67E-06	7.14E-06
**COL16A1**	2.75127472	7.40E-13	1.36E-11
**RAB38**	2.75696733	5.94E-13	1.14E-11
**SCN3B**	2.75798626	1.90E-09	1.10E-08
**SLCO4A1**	2.7613465	2.58E-08	1.09E-07
**MLF1**	2.76174018	1.22E-10	1.01E-09
**SLC24A3**	2.76237666	3.18E-10	2.31E-09
**UNC13A**	2.76551259	8.11E-11	7.13E-10
**HS3ST5**	2.766975	1.04E-06	3.04E-06
**TDRD9**	2.76923932	3.29E-10	2.38E-09
**PPP1R1B**	2.76962121	3.12E-06	8.23E-06
**WNT9A**	2.76969289	1.23E-06	3.54E-06
**CHRNA1**	2.77299641	5.85E-07	1.81E-06
**PLCD3**	2.77499659	8.40E-17	9.94E-15
**LRRC15**	2.77578829	0.00028553	0.00052684
**RCOR2**	2.78195644	2.36E-12	3.61E-11
**NAT8L**	2.78288756	2.80E-06	7.45E-06
**CATSPER1**	2.78307006	3.53E-14	1.14E-12
**GRM8**	2.78375974	4.28E-12	5.89E-11
**L1TD1**	2.78379908	0.00027354	0.00050628
**PDPN**	2.78424374	1.14E-10	9.49E-10
**COL9A2**	2.79310087	5.42E-14	1.59E-12
**ACTC1**	2.79429278	3.33E-07	1.09E-06
**NBL1**	2.7946505	7.14E-17	8.90E-15
**CXCL1**	2.79693435	3.31E-09	1.79E-08
**CTXN1**	2.79908402	1.54E-14	5.88E-13
**HHLA2**	2.80122826	4.34E-08	1.73E-07
**GALNT14**	2.80644055	0.00015651	0.00030303
**ARMC3**	2.80660387	1.06E-07	3.89E-07
**RNF182**	2.80748794	3.93E-10	2.77E-09
**ESRP1**	2.8144098	2.97E-08	1.24E-07
**PRAME**	2.81702255	4.60E-06	1.18E-05
**RARRES1**	2.82103308	1.48E-08	6.68E-08
**S100A8**	2.82412009	8.64E-07	2.57E-06
**ZNF469**	2.82704677	1.65E-09	9.70E-09
**KRT81**	2.82975456	3.71E-06	9.65E-06
**SIX2**	2.83037554	2.27E-07	7.71E-07
**TMC5**	2.83656919	2.99E-10	2.18E-09
**TMC4**	2.83706214	1.62E-10	1.28E-09
**KREMEN2**	2.83922492	7.65E-11	6.83E-10
**SH2D3A**	2.84471125	4.17E-10	2.91E-09
**CA12**	2.84589973	1.42E-06	4.04E-06
**PPAPDC1A**	2.84609235	1.68E-07	5.89E-07
**CLEC5A**	2.84878156	1.12E-16	1.24E-14
**STRA6**	2.84943129	3.62E-12	5.13E-11
**TPBG**	2.85171085	5.14E-12	6.78E-11
**RSPH1**	2.85193646	1.62E-09	9.54E-09

**FAM46B**	2.85501541	2.19E-13	5.01E-12
**KCNN4**	2.85538786	5.59E-11	5.22E-10
**VILL**	2.85588348	1.77E-11	1.95E-10
**DUOXA2**	2.8565433	3.34E-07	1.09E-06
**TREM1**	2.8567776	7.75E-14	2.14E-12
**FREM1**	2.85733557	4.92E-05	0.00010388
**CHGB**	2.86116101	4.56E-06	1.17E-05
**NRG3**	2.86264656	4.72E-06	1.20E-05
**FOXL1**	2.86816094	2.57E-14	8.80E-13
**SLC35F2**	2.86861939	8.07E-16	5.77E-14
**CYS1**	2.87205733	3.83E-07	1.24E-06
**CKMT1B**	2.8734936	1.66E-07	5.83E-07
**S100A11**	2.87809392	1.06E-16	1.21E-14
**KISS1R**	2.8822134	3.06E-08	1.27E-07
**ANO9**	2.8856763	4.23E-10	2.95E-09
**GGT6**	2.89604442	3.90E-07	1.26E-06
**LCNL1**	2.89742663	3.77E-06	9.78E-06
**SPATA12**	2.90100935	1.50E-14	5.79E-13
**ALOX12B**	2.90101477	2.62E-06	7.03E-06
**GPR1**	2.90614959	1.67E-13	3.97E-12
**DLX4**	2.90773258	3.90E-11	3.86E-10
**MDFI**	2.90807459	6.55E-11	5.98E-10
**CREB3L1**	2.91031949	2.68E-10	1.99E-09
**SOX8**	2.92092167	1.04E-06	3.05E-06
**PDZD3**	2.92483355	0.00032578	0.00059463
**S100A4**	2.93203055	2.56E-16	2.24E-14
**CKM**	2.9345483	1.94E-11	2.11E-10
**CCDC74A**	2.94042868	4.45E-14	1.38E-12
**KCNJ1**	2.94175651	1.12E-05	2.66E-05
**PPAP2C**	2.9496054	5.54E-12	7.25E-11
**SLC4A11**	2.9517921	2.22E-14	7.82E-13
**NCCRP1**	2.95834228	1.64E-07	5.74E-07
**GRAMD2**	2.9589662	1.05E-09	6.50E-09
**TESC**	2.96665594	3.84E-07	1.24E-06
**F3**	2.97131917	3.02E-12	4.42E-11
**IGSF5**	2.97908646	9.14E-07	2.71E-06
**PDLIM4**	2.98327485	1.16E-12	1.99E-11
**CSF2**	2.98668211	2.68E-06	7.18E-06
**NLRP2**	2.98893861	8.64E-06	2.09E-05
**DUSP27**	2.98958596	0.00050117	0.00088339
**PAEP**	2.99038193	2.23E-09	1.27E-08
**CDCP1**	2.99204438	7.49E-10	4.84E-09
**DAZL**	2.99970024	3.46E-05	7.50E-05
**CCL26**	2.9998206	3.42E-10	2.45E-09
**APCDD1L**	3.00190698	3.70E-07	1.20E-06
**C3orf55**	3.00271121	6.24E-08	2.41E-07
**CHST6**	3.00342412	6.79E-09	3.37E-08
**IL6**	3.01098253	5.12E-06	1.29E-05
**C21orf88**	3.01397719	1.62E-07	5.68E-07
**NKAIN1**	3.01565876	5.26E-08	2.07E-07
**CKMT1A**	3.02318062	2.55E-06	6.86E-06
**CD1A**	3.02731108	9.88E-07	2.90E-06
**CDR2L**	3.027528	3.71E-15	1.89E-13
**NLGN1**	3.02910272	0.00010551	0.00021055
**GSTP1**	3.0296666	7.90E-14	2.15E-12
**GLB1L2**	3.0311078	3.32E-09	1.79E-08
**MMP28**	3.03876747	8.22E-11	7.20E-10
**CACNA1G**	3.03880314	1.99E-10	1.53E-09
**TMEM163**	3.03892563	1.53E-10	1.23E-09
**TUBB3**	3.04106866	1.20E-13	3.08E-12
**NKAIN4**	3.04515723	4.28E-08	1.71E-07
**ZPLD1**	3.04596515	1.45E-11	1.66E-10
**EYA1**	3.04688272	5.70E-05	0.00011921
**GALNT12**	3.05646758	1.23E-11	1.43E-10
**EPHA10**	3.05916614	2.18E-08	9.37E-08
**SLC16A3**	3.06809302	5.37E-19	2.27E-16
**C19orf59**	3.06918793	5.07E-10	3.45E-09
**UNC5A**	3.07171384	4.25E-06	1.09E-05
**FANK1**	3.07937655	1.18E-13	3.04E-12
**MAGEA4**	3.08261207	0.00018297	0.00035022

**EREG**	3.087511	3.83E-12	5.37E-11
**FAT2**	3.09111109	1.79E-10	1.39E-09
**KCNJ16**	3.09441332	1.18E-07	4.27E-07
**DMKN**	3.09595655	1.15E-05	2.71E-05
**CRCT1**	3.10762677	0.00043108	0.00076954
**SH2D5**	3.11481576	7.10E-09	3.50E-08
**NIPAL4**	3.11888691	9.48E-05	0.00019068
**FOXF2**	3.12162883	8.79E-12	1.07E-10
**ANK1**	3.12329228	1.29E-07	4.63E-07
**HS3ST1**	3.12401149	3.16E-12	4.61E-11
**ENO2**	3.12416739	1.61E-13	3.87E-12
**LRRIQ1**	3.1327264	4.87E-09	2.50E-08
**MGAT3**	3.13458809	2.34E-11	2.47E-10
**GRM3**	3.13664094	4.79E-06	1.22E-05
**CCDC144NL**	3.14536753	9.40E-05	0.00018922
**CXCL3**	3.14621534	4.86E-08	1.92E-07
**PF4V1**	3.15286272	3.72E-08	1.51E-07
**SLC11A1**	3.1617458	3.53E-14	1.14E-12
**NXNL2**	3.16599569	5.84E-08	2.27E-07
**TUBAL3**	3.17347098	1.63E-06	4.55E-06
**RAB36**	3.18416243	3.05E-11	3.12E-10
**HOXC13**	3.18447335	4.35E-10	3.02E-09
**LYPD6**	3.18468947	1.02E-09	6.36E-09
**ACTL6B**	3.19113696	4.14E-06	1.07E-05
**S100B**	3.19525809	2.52E-09	1.41E-08
**MIA**	3.19813976	7.18E-06	1.77E-05
**CRABP1**	3.2056184	0.00010437	0.00020864
**ENTHD1**	3.20962426	7.56E-07	2.28E-06
**CCDC64B**	3.21406009	5.92E-10	3.95E-09
**SPEG**	3.22827901	4.35E-05	9.24E-05
**CCDC74B**	3.23096878	9.24E-14	2.46E-12
**PRSS21**	3.23199375	2.72E-05	5.98E-05
**EPHX3**	3.23407323	9.35E-12	1.13E-10
**EGFL6**	3.23517969	2.63E-14	8.93E-13
**PYDC1**	3.23537993	1.13E-05	2.67E-05
**SPINT1**	3.24422562	7.90E-14	2.15E-12
**MFAP2**	3.25727338	5.93E-15	2.81E-13
**EVPL**	3.27178808	4.00E-07	1.29E-06
**TNFAIP6**	3.27326319	4.05E-15	2.03E-13
**HR**	3.27358915	2.03E-12	3.16E-11
**TMEM130**	3.273829	3.95E-10	2.78E-09
**SEMA3C**	3.28228247	5.76E-13	1.11E-11
**CLIC3**	3.28256395	2.29E-13	5.17E-12
**AK5**	3.28526488	1.06E-09	6.53E-09
**FOXJ1**	3.28645186	5.17E-08	2.03E-07
**S100A3**	3.28744448	6.09E-21	1.22E-17
**TWIST1**	3.29224628	4.66E-10	3.21E-09
**EN1**	3.29398941	1.77E-05	4.04E-05
**SLC2A1**	3.30069326	6.13E-13	1.16E-11
**IFNE**	3.30505952	1.16E-08	5.38E-08
**SLC4A3**	3.3050782	1.76E-10	1.38E-09
**SLC6A20**	3.30966854	0.00018632	0.00035607
**B4GALNT3**	3.31645056	0.00052533	0.00092325
**AREG**	3.32026279	5.70E-08	2.22E-07
**MFI2**	3.324637	5.94E-13	1.14E-11
**HTR3A**	3.32680457	1.06E-07	3.89E-07
**ZNF365**	3.32706107	3.79E-12	5.32E-11
**FOLR1**	3.32742954	7.70E-09	3.76E-08
**CYP2S1**	3.33282811	4.63E-12	6.24E-11
**RETN**	3.33314435	6.72E-06	1.66E-05
**ACTBL2**	3.34009203	3.16E-09	1.71E-08
**FAM177B**	3.3464739	1.08E-07	3.96E-07
**ABCA12**	3.34995727	6.17E-07	1.90E-06
**IL20RA**	3.35977175	2.38E-09	1.34E-08
**ATP6V0A4**	3.36157331	3.64E-05	7.86E-05
**GRM1**	3.36824504	3.52E-07	1.15E-06
**IL22RA2**	3.37102885	2.71E-05	5.98E-05
**SGPP2**	3.37197298	9.78E-09	4.63E-08
**ARHGEF38**	3.37882967	1.79E-08	7.92E-08
**LYPD3**	3.3801912	2.69E-06	7.20E-06

**S100A5**	3.38035168	4.85E-12	6.49E-11
**GRIN2A**	3.39342477	1.22E-05	2.87E-05
**SEMA3E**	3.40106539	3.73E-07	1.21E-06
**ATP13A4**	3.41141177	0.00015882	0.00030717
**LEFTY2**	3.41255112	0.00020615	0.000391
**CASP14**	3.41546612	0.00022451	0.00042269
**SLN**	3.41929338	9.02E-10	5.69E-09
**RXFP4**	3.42755047	5.00E-08	1.98E-07
**GRHL2**	3.42959354	1.27E-05	2.98E-05
**NMNAT2**	3.43524852	4.76E-11	4.59E-10
**GAL**	3.43757125	1.42E-06	4.04E-06
**C6orf132**	3.43772044	1.73E-14	6.37E-13
**KRT80**	3.43773429	2.11E-13	4.86E-12
**SYNGR3**	3.44084335	2.23E-12	3.43E-11
**DMBX1**	3.44222275	0.00026479	0.00049178
**PPP1R14D**	3.44504831	1.16E-11	1.36E-10
**PPP2R2C**	3.44886989	6.15E-10	4.08E-09
**TMEM72**	3.45005811	0.00034729	0.0006301
**DUOXA1**	3.45127488	1.25E-06	3.60E-06
**TMEM132A**	3.45417431	5.80E-18	1.21E-15
**CRYBA2**	3.46328436	0.00026318	0.00048908
**TNNI3**	3.4673454	1.37E-08	6.23E-08
**C1orf116**	3.46767048	4.05E-08	1.63E-07
**SCN2B**	3.46953495	0.00010081	0.00020202
**EPHB3**	3.47174673	8.29E-11	7.25E-10
**GBX2**	3.47953959	1.41E-08	6.38E-08
**GAGE2C**	3.48009628	0.00033998	0.00061817
**MST1R**	3.48060249	2.99E-09	1.63E-08
**WNK2**	3.50247037	1.45E-07	5.16E-07
**TRPV6**	3.5088167	1.30E-06	3.71E-06
**WNT10A**	3.51441853	2.94E-08	1.22E-07
**PFKP**	3.51762544	3.00E-14	9.89E-13
**NUDT11**	3.52166837	4.78E-09	2.46E-08
**CCDC160**	3.52286749	3.05E-07	1.01E-06
**LGALS7B**	3.52953711	4.46E-05	9.47E-05
**VWA2**	3.5313206	3.44E-05	7.46E-05
**TMEM125**	3.53423277	2.27E-05	5.08E-05
**PMEPA1**	3.54327093	8.44E-12	1.03E-10
**COL10A1**	3.54491857	1.76E-10	1.38E-09
**FAM131C**	3.5558317	6.83E-13	1.27E-11
**SLITRK2**	3.56336838	0.00010958	0.00021775
**BIRC7**	3.56498906	5.01E-13	9.72E-12
**LAMA1**	3.57443495	1.64E-12	2.65E-11
**LMX1B**	3.57956453	1.23E-05	2.88E-05
**RASAL1**	3.59532085	1.09E-10	9.17E-10
**MNX1**	3.5956783	6.04E-06	1.50E-05
**MAPK15**	3.5972283	1.09E-06	3.16E-06
**HCAR1**	3.60265763	3.72E-05	8.02E-05
**FAM150A**	3.60729923	0.00028987	0.00053418
**PTPRR**	3.61123832	3.71E-06	9.65E-06
**INHA**	3.61751723	8.32E-07	2.49E-06
**PCYT1B**	3.61782576	1.13E-08	5.25E-08
**PLA2G4E**	3.62340805	0.00017064	0.00032829
**AP3B2**	3.62613955	6.85E-10	4.49E-09
**COMP**	3.63121245	2.16E-11	2.31E-10
**HSPB3**	3.64762595	0.00010502	0.00020989
**SCTR**	3.64906844	3.02E-05	6.61E-05
**TDRD5**	3.6495078	1.26E-07	4.53E-07
**FSCN2**	3.66674122	1.48E-10	1.20E-09
**KRT20**	3.66723927	3.55E-05	7.67E-05
**SPINT2**	3.67194879	3.00E-11	3.08E-10
**TFCP2L1**	3.67550067	5.05E-10	3.44E-09
**GGTLC1**	3.67834334	0.00032907	0.00059988
**DNER**	3.67997154	1.11E-08	5.17E-08
**MFSD6L**	3.68007538	4.86E-09	2.50E-08
**CCNA1**	3.6909611	3.22E-06	8.49E-06
**SPOCD1**	3.69601163	1.38E-15	8.66E-14
**NPTX2**	3.69849145	5.94E-06	1.48E-05
**COX6B2**	3.70233986	1.12E-10	9.32E-10
**COL11A1**	3.72237359	5.96E-12	7.68E-11

**S100A9**	3.74365565	5.59E-11	5.22E-10
**RAB25**	3.75012219	2.99E-06	7.92E-06
**SCIN**	3.75243758	6.64E-17	8.48E-15
**KLK1**	3.75790134	9.50E-09	4.51E-08
**CHST4**	3.75936657	9.16E-09	4.37E-08
**ANXA8L2**	3.75963288	1.60E-07	5.64E-07
**CCL7**	3.76352371	5.24E-11	4.96E-10
**GAST**	3.76387624	0.00021586	0.0004077
**SPIB**	3.76741745	1.83E-07	6.34E-07
**ATP10B**	3.76952053	1.30E-09	7.81E-09
**DES**	3.77732605	1.56E-05	3.59E-05
**GDF1**	3.78339122	5.44E-08	2.13E-07
**CDSN**	3.80412213	2.51E-08	1.06E-07
**C9orf129**	3.80660444	1.07E-07	3.93E-07
**ECEL1**	3.81280327	5.05E-08	1.99E-07
**GAGE2B**	3.81428278	0.00016141	0.00031159
**SLC28A3**	3.82177144	3.11E-10	2.26E-09
**MACC1**	3.84577764	1.26E-12	2.12E-11
**GJB5**	3.84810141	0.00018516	0.00035391
**VEPH1**	3.87252265	9.37E-08	3.47E-07
**IGFL2**	3.87741622	2.74E-09	1.51E-08
**SOX11**	3.87973234	1.15E-12	1.98E-11
**C3orf52**	3.88930239	1.98E-16	1.91E-14
**PSORS1C1**	3.89048144	2.04E-10	1.56E-09
**MMP8**	3.89179391	1.98E-06	5.45E-06
**GPA33**	3.91279627	8.12E-09	3.93E-08
**CILP2**	3.91400333	7.75E-11	6.91E-10
**HOXB8**	3.91715992	3.40E-06	8.91E-06
**PRRX2**	3.93145695	2.01E-11	2.17E-10
**GABRR1**	3.9380277	2.61E-05	5.77E-05
**AHNAK2**	3.9388992	2.09E-08	9.04E-08
**PSORS1C2**	3.94267162	3.26E-07	1.07E-06
**PTPRZ1**	3.94401513	3.25E-06	8.55E-06
**ITGB8**	3.95692663	8.27E-12	1.01E-10
**DUOX2**	3.96728308	2.32E-08	9.93E-08
**FBN2**	3.9749323	7.53E-07	2.27E-06
**SNAP91**	3.97767465	2.41E-06	6.52E-06
**SLC35F3**	3.97810714	1.78E-08	7.90E-08
**NDP**	3.99037832	6.38E-09	3.18E-08
**PROM2**	4.00394536	2.10E-07	7.18E-07
**ITGA3**	4.00539319	3.00E-16	2.48E-14
**S100A6**	4.00618754	4.09E-19	2.06E-16
**ITGB4**	4.01072209	1.67E-14	6.21E-13
**RGS7**	4.03556198	5.24E-07	1.64E-06
**LHFPL3**	4.03870443	5.35E-09	2.72E-08
**ANXA3**	4.03899724	6.32E-09	3.16E-08
**ZG16B**	4.04732739	5.02E-07	1.58E-06
**CAPN8**	4.05080171	0.00020311	0.00038568
**LRRN1**	4.05625739	1.46E-05	3.37E-05
**IGSF11**	4.05678337	2.43E-11	2.55E-10
**HES2**	4.06195266	3.07E-09	1.67E-08
**AGR2**	4.06737896	1.31E-08	6.00E-08
**MSX2**	4.06975854	4.28E-08	1.71E-07
**A4GNT**	4.07098499	6.22E-07	1.92E-06
**STYK1**	4.07443091	3.17E-07	1.04E-06
**AJAP1**	4.07462888	0.0002361	0.00044307
**WNT7A**	4.07717194	4.08E-05	8.73E-05
**FAM101A**	4.10814647	2.25E-09	1.27E-08
**ACAN**	4.11084432	1.36E-13	3.42E-12
**ANKRD1**	4.13064158	1.40E-05	3.26E-05
**TRIM72**	4.14288958	5.22E-06	1.32E-05
**PRKCG**	4.14851916	1.10E-10	9.19E-10
**GDPD2**	4.14969411	6.42E-11	5.88E-10
**NDST4**	4.15166742	8.00E-07	2.40E-06
**PLEKHB1**	4.154664	1.95E-13	4.54E-12
**CA9**	4.15826698	1.80E-09	1.05E-08
**FOXD1**	4.17345688	3.16E-08	1.31E-07
**OVOL2**	4.18387914	3.30E-09	1.78E-08
**KCNG3**	4.1841786	3.78E-08	1.53E-07
**PRSS1**	4.1883246	0.00027219	0.0005042

**FRMD5**	4.18982964	3.55E-11	3.56E-10
**PVRL4**	4.19632041	4.23E-08	1.70E-07
**C9orf135**	4.20865229	8.86E-05	0.00017904
**ATP1A3**	4.24618661	4.12E-06	1.06E-05
**MMP7**	4.25672907	6.48E-13	1.22E-11
**ITGB6**	4.28914052	1.93E-09	1.11E-08
**ATP2C2**	4.29122814	7.44E-08	2.82E-07
**MYBPC2**	4.2966002	3.85E-08	1.56E-07
**SIX3**	4.30919317	4.36E-05	9.27E-05
**EPN3**	4.3098648	8.84E-11	7.64E-10
**RUNDC3A**	4.31255315	3.05E-13	6.53E-12
**GJB4**	4.31867617	5.21E-06	1.31E-05
**LIPH**	4.32214298	5.22E-10	3.54E-09
**FIBCD1**	4.34603458	6.27E-10	4.15E-09
**CYP24A1**	4.35433441	3.11E-07	1.03E-06
**SLC26A9**	4.36198861	1.33E-11	1.53E-10
**MMP12**	4.364707	6.78E-11	6.17E-10
**KRT79**	4.36924506	3.71E-14	1.19E-12
**FAM71E2**	4.37519711	1.26E-07	4.53E-07
**C2orf70**	4.40336987	3.41E-07	1.11E-06
**PGLYRP4**	4.42130432	1.43E-06	4.06E-06
**IGFL3**	4.44233405	9.25E-05	0.00018638
**STAC2**	4.44564487	1.67E-05	3.83E-05
**B3GALT5**	4.45539823	3.44E-08	1.41E-07
**TFF1**	4.46081558	9.66E-11	8.25E-10
**MUC12**	4.46120823	7.16E-11	6.46E-10
**PLA2G10**	4.46170969	2.04E-09	1.17E-08
**CRABP2**	4.46390304	7.59E-10	4.90E-09
**ZNF750**	4.47676271	0.00021503	0.00040641
**GUCA1A**	4.49886049	6.03E-06	1.50E-05
**CHIT1**	4.51953986	1.51E-05	3.49E-05
**VSIG1**	4.526719	9.58E-08	3.55E-07
**LYPD6B**	4.53582879	3.84E-09	2.04E-08
**SFRP5**	4.55113589	3.56E-05	7.69E-05
**GJB3**	4.55967834	1.89E-09	1.09E-08
**ALOXE3**	4.58354955	3.47E-08	1.42E-07
**COL17A1**	4.58378786	8.60E-08	3.21E-07
**GPR110**	4.58928391	0.00021543	0.00040711
**SPDEF**	4.62347422	1.28E-09	7.72E-09
**SCGB1A1**	4.63397458	5.43E-05	0.00011394
**B4GALNT4**	4.64407636	4.61E-08	1.83E-07
**WFDC10B**	4.66308179	4.00E-07	1.29E-06
**VTCN1**	4.66737215	5.05E-06	1.28E-05
**ZBBX**	4.68839618	0.00046036	0.00081715
**ZNF488**	4.68924545	7.99E-09	3.88E-08
**GPRIN2**	4.7065155	2.77E-09	1.53E-08
**PKP1**	4.72104272	4.96E-08	1.96E-07
**FAM83B**	4.73872557	2.74E-09	1.51E-08
**GALNT5**	4.74557022	3.52E-11	3.53E-10
**SLC36A2**	4.77664304	0.0003176	0.00058109
**DRD2**	4.78600621	2.24E-05	5.03E-05
**CFTR**	4.79316911	5.03E-06	1.27E-05
**CLDN10**	4.81106317	1.69E-05	3.88E-05
**TNNT1**	4.81385861	3.12E-13	6.63E-12
**EPS8L1**	4.82188117	7.23E-13	1.34E-11
**SCG3**	4.82665968	2.19E-05	4.90E-05
**PRSS22**	4.82921815	4.38E-10	3.04E-09
**PROM1**	4.83829441	4.86E-11	4.67E-10
**HMX3**	4.86558109	3.46E-05	7.50E-05
**SLC5A1**	4.89049504	1.26E-07	4.55E-07
**IL1A**	4.90347408	1.26E-12	2.13E-11
**PAX9**	4.91625648	2.24E-07	7.60E-07
**TACSTD2**	4.92074912	2.58E-09	1.44E-08
**SFRP2**	4.92123303	9.15E-13	1.64E-11
**C6orf222**	4.92172869	1.48E-09	8.82E-09
**CDK5R2**	4.94796922	3.55E-07	1.15E-06
**ARL14**	4.95603386	8.03E-11	7.08E-10
**GP2**	4.98719782	0.00047297	0.00083689
**ART3**	5.00850402	5.99E-06	1.49E-05
**PI3**	5.01334727	1.31E-08	6.00E-08

**CST6**	5.03677276	2.50E-17	3.90E-15
**SPACA4**	5.05803059	6.28E-06	1.56E-05
**EPHA6**	5.08430174	9.13E-10	5.76E-09
**SLC6A14**	5.10995394	6.45E-11	5.90E-10
**APOBEC1**	5.13578236	0.00013843	0.00027057
**ITIH5**	5.13703778	1.01E-08	4.77E-08
**SYT8**	5.13989188	1.65E-07	5.80E-07
**FAM3D**	5.15027398	3.07E-05	6.72E-05
**ANXA8**	5.15940934	1.20E-07	4.34E-07
**HOXB9**	5.16715929	9.80E-06	2.35E-05
**KLK13**	5.17380676	3.12E-07	1.03E-06
**PIP**	5.1755294	1.07E-05	2.53E-05
**RHOV**	5.19485381	1.01E-15	6.85E-14
**SLC34A2**	5.19771199	2.24E-08	9.62E-08
**ANXA8L1**	5.21056417	1.20E-07	4.34E-07
**SYT13**	5.22833659	5.12E-10	3.48E-09
**CAPN6**	5.23166278	9.95E-08	3.67E-07
**NTF4**	5.24353502	0.00020263	0.00038488
**SPINK13**	5.25292055	4.39E-05	9.33E-05
**AGR3**	5.32897271	6.94E-06	1.71E-05
**MUC2**	5.33952214	5.17E-06	1.30E-05
**PPP1R14C**	5.34987843	1.54E-15	9.38E-14
**S100A7**	5.41276039	2.27E-06	6.17E-06
**OR10Q1**	5.42069773	1.91E-05	4.32E-05
**ST6GALNAC1**	5.45800165	1.25E-05	2.93E-05
**MYO3A**	5.48533778	1.22E-07	4.41E-07
**PTPRN**	5.50745756	9.16E-11	7.88E-10
**TTYH1**	5.50959526	1.05E-07	3.87E-07
**PSAPL1**	5.53279013	9.81E-09	4.64E-08
**NPTX1**	5.54130406	2.67E-09	1.48E-08
**SORCS1**	5.55011987	7.31E-10	4.74E-09
**GPR115**	5.55188756	3.20E-13	6.78E-12
**FXYD3**	5.56039151	8.56E-10	5.45E-09
**VGF**	5.58245965	6.16E-07	1.90E-06
**CGB8**	5.5878187	2.71E-09	1.50E-08
**L1CAM**	5.5923913	6.91E-12	8.70E-11
**GRP**	5.59451027	5.42E-14	1.59E-12
**CDH3**	5.61738936	1.77E-11	1.95E-10
**WNT7B**	5.72749256	7.47E-12	9.28E-11
**GABRP**	5.7618608	8.63E-06	2.09E-05
**SLC44A4**	5.77303768	4.07E-11	4.01E-10
**CXCL5**	5.7776617	4.86E-11	4.67E-10
**SERPINB4**	5.815383	1.51E-06	4.27E-06
**PKP3**	5.83255615	2.92E-14	9.67E-13
**C11orf53**	5.83568278	1.38E-12	2.30E-11
**IGFL1**	5.84191324	5.30E-05	0.00011133
**CLDN6**	5.86589717	8.63E-06	2.09E-05
**FAM83E**	5.89535182	3.33E-09	1.79E-08
**FA2H**	5.89569277	6.28E-14	1.80E-12
**ERN2**	5.92206964	1.73E-09	1.01E-08
**CD164L2**	5.92213478	3.86E-10	2.72E-09
**MUC1**	5.93923594	6.16E-09	3.09E-08
**CLLU1OS**	5.95279785	2.54E-14	8.72E-13
**CALB2**	5.95925045	1.83E-11	2.01E-10
**SBSN**	5.96823561	8.67E-05	0.00017561
**CSRP3**	5.97754593	5.43E-06	1.37E-05
**C12orf36**	5.98001117	2.93E-13	6.32E-12
**LAMC2**	6.02131903	1.39E-12	2.31E-11
**CDH17**	6.04132246	7.40E-14	2.05E-12
**ALDH3B2**	6.06181221	2.80E-07	9.34E-07
**WFDC2**	6.08346419	4.39E-11	4.29E-10
**KERA**	6.15151928	5.41E-05	0.00011348
**NPPB**	6.1589415	2.48E-08	1.05E-07
**UPK1B**	6.1881852	1.02E-05	2.44E-05
**C19orf33**	6.26364484	1.66E-11	1.86E-10
**CLLU1**	6.31608771	3.20E-08	1.32E-07
**GRM4**	6.31616084	7.08E-07	2.15E-06
**CTSE**	6.34505022	5.11E-11	4.88E-10
**KRT19**	6.50306243	2.08E-13	4.80E-12
**KRT6B**	6.50950799	3.53E-10	2.52E-09

**DSC3**	6.51784811	4.83E-08	1.91E-07
**PDCL2**	6.56284498	5.51E-07	1.72E-06
**SDR16C5**	6.57497048	1.60E-06	4.49E-06
**SCEL**	6.69653908	1.19E-08	5.51E-08
**GPRC5A**	6.82673877	1.03E-10	8.69E-10
**MMP13**	6.90067417	2.51E-08	1.07E-07
**DAPL1**	6.97217313	0.00012967	0.00025441
**MSLNL**	7.01264512	1.02E-08	4.79E-08
**TFF2**	7.09638984	8.35E-10	5.32E-09
**BARX2**	7.29440916	1.91E-11	2.08E-10
**SPINK4**	7.29510215	0.00014309	0.00027876
**RAET1L**	7.44217337	1.29E-07	4.63E-07
**SERPINB7**	7.44458474	1.81E-07	6.28E-07
**PSCA**	7.52336765	5.74E-12	7.46E-11
**S100A2**	7.54783729	1.07E-12	1.86E-11
**SERPINB3**	7.6661496	5.02E-08	1.98E-07
**CLCA2**	7.68557871	9.24E-06	2.23E-05
**NMU**	7.69386197	4.24E-08	1.70E-07
**KLK11**	7.71522957	6.98E-05	0.00014367
**KLK10**	7.76707837	4.13E-06	1.06E-05
**CACNG6**	7.85001073	3.11E-05	6.78E-05
**KLK6**	7.97771858	9.26E-09	4.41E-08
**TCN1**	8.05806832	8.07E-11	7.11E-10
**SERPINB2**	8.06584025	2.33E-09	1.31E-08
**C10orf99**	8.07575033	0.00014634	0.00028468
**KLK8**	8.10562532	6.29E-08	2.42E-07
**TMPRSS4**	8.13486894	3.71E-07	1.20E-06
**A2ML1**	8.1479748	1.79E-05	4.09E-05
**KRT17**	8.29428674	4.93E-16	3.70E-14
**CLDN18**	8.30547383	8.87E-15	3.88E-13
**PGLYRP3**	8.62146423	1.37E-06	3.89E-06
**LEMD1**	8.63025532	2.74E-06	7.32E-06
**C1orf110**	8.72078923	5.10E-05	0.00010734
**SERPINB5**	8.72176125	4.73E-06	1.20E-05
**KRT15**	8.94762347	4.14E-17	5.95E-15
**KLK5**	9.19549681	2.81E-08	1.18E-07
**CEACAM6**	9.20049136	4.51E-08	1.80E-07
**MSLN**	9.46250374	9.65E-12	1.16E-10
**KLK12**	9.55246709	4.68E-06	1.19E-05
**KRT4**	9.7954011	3.60E-07	1.17E-06
**KLK7**	9.98938612	1.31E-06	3.73E-06
**MUC17**	10.0819874	2.66E-06	7.13E-06
**FGFBP1**	10.1014214	6.38E-06	1.58E-05
**KRT16**	10.6561727	9.86E-11	8.40E-10
**KRT5**	10.830434	1.85E-09	1.08E-08
**CEACAM7**	10.99055	1.45E-12	2.39E-11
**SPRR1A**	11.4389243	0.00010435	0.00020864
**KRT14**	11.544532	7.64E-10	4.93E-09
**SPRR3**	12.1585806	1.16E-05	2.74E-05
**CEACAM5**	12.7006358	1.20E-10	9.99E-10
**LY6D**	13.0229035	2.56E-07	8.59E-07
**OLFM4**	13.1295306	6.47E-13	1.22E-11
**SPRR1B**	14.2689466	0.00011101	0.00022022
**KRT13**	14.5310523	8.37E-07	2.50E-06
**KRT6A**	14.6482114	4.81E-09	2.48E-08
